# Congrès de la SFMTSI - Santé en Méditerranée Pathologies - Migrations - Environnement 22-24 mai 2024 Saint-Raphaël, France

**DOI:** 10.48327/mtsi.v4i2.2024.529

**Published:** 2024-06-13

**Authors:** Jean JANNIN

**Affiliations:** SFMTSI Hôpital Pitié-Salpêtrière - Pavillon Laveran, 47-83 Boulevard de l’Hôpital, 75013 Paris, France

**Président :** Jean JANNIN

**Comité d’organisation :** Karim AOUN, Jean JANNIN, Pierre MARTY, Jean TESTA

**Comité scientifique :** Marco ALBONICO (Italie), Aida BOURATBINE (Tunisie), Eric CAUMES (président), Paul-Henry CONSIGNY (SMV), Badre Eddine LMIMOUNI (Maroc), Vincent LE MOING (SPILF), Eric PICHARD (SFMTSI), Jorge SEIXAS (Portugal), Nicolas VIGNIER (CMIT), Mohammed YOUSFI (Algérie)

## Introduction

L’objectif de la Société francophone de médecine tropicale et santé internationale (SFMTSI), en organisant le congrès « Santé en Méditerranée », est de réunir les principales sociétés savantes dédiées à la médecine tropicale et à la santé internationale du pourtour méditerranéen pour échanger sur les problématiques qui, chaque jour, mobilisent notre énergie et notre intelligence.

L’espace méditerranéen est remarquable à plus d’un titre : il recueille les héritages de l’Antiquité et constitue depuis des siècles un « espace singulier porteur d’une dynamique créatrice qui s’appelle la civilisation » comme le rappelle Paul Valéry.

Notre *Mare mediterraneum,* « mer au milieu des terres », espace de civilisations, espace de conflits, espace d’échanges, d’art, de science et de philosophie est avant tout un formidable espace de rencontres que ce congrès souhaite célébrer.

L’environnement de cet espace méditerranéen est tout à fait remarquable et les problèmes sanitaires qui s’y développent ne peuvent être caractérisés ni traités sans une compréhension plus intime des interrelations entre nos différentes cultures.

C’est donc, en partenariat avec neuf pays (Algérie, Espagne, Italie, France, Liban, Maroc, Monaco, Portugal, Tunisie), que ce congrès explore trois déterminants essentiels de la santé : les pathologies méditerranéennes et tropicales, les mouvements migratoires, leurs caractéristiques et conséquences sanitaires, et les problématiques environnementales du bassin méditerranéen.

Espérons que ce congrès ira largement au-delà des aspects médicaux et scientifiques et fera l’objet de nombreux débats entre participants et participantes. Espérons aussi que cette rencontre contribuera à resserrer les liens entre les différents acteurs de la « santé en Méditerranée » et augurera d’un renforcement des liens et des nécessaires coopérations.

