# Supplementary material for: SFMTSI Congress - Health in the Mediterranean Pathologies - Migrations - Environment 22-24 May 2024 Saint-Raphaël, France
Source: Med Trop Sante Int. 2024 Jun 13;4(2):mtsi.v4i2.2024.529. [Article in French] doi: 10.48327/mtsi.v4i2.2024.529 (PMC11292441; doi:10.48327/mtsi.v4i2.2024.529)
Supplement: Related texts

# Research article: La Méditerranée, éternel creuset de civilisations : les aspects médicaux

**Published:** 2024-06-13

**Authors:** Driss MOUSSAOUI, Association mondiale de psychiatrie sociale

**Affiliations:** Ancien président, Fédération mondiale de psychothérapie Membre associé étranger, Académie nationale de médecine

Sur un planisphère, la mer Méditerranée représente 0,5 % de la surface totale de la planète. Et pourtant, loin devant la vallée du Gange, l’entre deux fleuves chinois et l’Amérique centrale, elle a abrité un très grand nombre de civilisations tout au long des 10 derniers millénaires.

Au plan médical, des écoles se sont constituées en Mésopotamie/Perse, en Égypte pharaonique, en Grèce, en Irak, en Andalousie et dans l’Europe du sud. L’intéressant dans cette chaîne est que la transmission s’est faite d’une civilisation à une autre, la proximité géographique aidant. Nous retrouvons ainsi, dans les traditions culturelles ayant trait à la santé et la maladie de nombre de pays méditerranéens, des modèles explicatifs décrits il y a plusieurs milliers d’années, et encore vivaces.

Des exemples seront donnés durant cet exposé.

# Research article: Implication de l'Institut Pasteur et de Pasteur Network dans l'espace méditerranéen

**Published:** 2024-06-13

**Authors:** Ronan JAMBOU

**Affiliations:** Directeur de Recherche à l’Institut Pasteur

Résumé non parvenu.

# Research article: Décrypter l'émergence de la bilharziose urogénitale dans le sud de l'Europe : une approche « One Health »

**Published:** 2024-06-13

**Authors:** Jérôme BOISSIER

**Affiliations:** Université de Perpignan Via Domitia, CNRS, IFREMER, Université de Montpellier, 58 Avenue Paul Alduy, Bat. R, 66860 Perpignan, France

Avril 2014, les premiers cas de bilharziose uro-génitale autochtone apparaissent en Europe. Au cours de cette première année, ce sont 106 cas qui sont identifiés avec comme zone de transmission avérée, la rivière du Cavu en Corse du sud. Après fermeture de la rivière à la baignade en 2014, des cas réapparaissent les années suivantes. En 2020, un nouvel événement surprenant marque cette émergence avec de nouveaux cas ayant pour origine une nouvelle rivière, la Solenzara. Il semble que nous assistions à l’invétération de la bilharziose urogénitale dans le sud de l’Europe. Afin de comprendre cette émergence inédite, plusieurs volets ont été examinés selon une approche « One Health ». Sur le volet humain, les manifestations cliniques ont été décrites et les outils de diagnostic ont été améliorés. Sur le volet environnemental, des enquêtes malacologiques ont été menées et de nouveaux outils d’ADN environnemental ont été développés. Sur le volet animal, deux vastes campagnes de piégeage de rongeurs et de diagnostics sérologiques sur le bétail ont été menées. Enfin, concernant l’agent étiologique, celui-ci a été caractérisé par des approches expérimentales et génomiques. Malgré tout le savoir accumulé, il reste encore une grande part de mystère sur cette émergence en particulier sur sa capacité à persister ou se reproduire dans l’avenir.

# Research article: Leishmanioses. Épidémiologie, problématiques et risques potentiels

**Published:** 2024-06-13

**Authors:** Karim AOUN

**Affiliations:** Laboratoire d’épidémiologie et d’écologie parasitaires, Institut Pasteur de Tunis, Tunisie

Deux formes de leishmanioses sont endémiques dans quasiment tous les pays méditerranéens, la leishmaniose viscérale (LV) et la leishmaniose cutanée (LC).

La LV est une maladie sporadique. Les incidences moyennes varient, selon les pays et les années, de quelques dizaines à quelques centaines de cas. Les pays méditerranéens les plus concernés sont ceux du Maghreb, l’Albanie, l’Espagne, l’Italie et la France. *Leishmania* (L.) *infantum,* agent causal, est zoonotique et le chien en est le réservoir principal. C’est un parasite opportuniste induisant des symptômes essentiellement chez les enfants et les adultes immunodéprimés. Les problèmes posés par la LV concernent surtout la prise en charge (PEC) avec la nécessité d’un diagnostic rapide et d’une thérapeutique appropriée.

Le profil épidémiologique de la LC est très différent de part et d’autre de la Méditerranée. Ainsi, si la LC à *L. infantum* est sporadique, à l’instar de la LV sur les deux rives, la LC zoonotique (LCZ) à *L. major* est fortement endémique dans les pays du Maghreb avec des milliers de cas annuels. Elle y représente un problème majeur en termes de morbidité, de PEC et de coût. L’émergence de la LCZ serait liée au développement de l’agriculture irriguée favorable à la pullulation des phlébotomes vecteurs et des rongeurs réservoirs ainsi qu’à l’urbanisation non contrôlée qui augmente l’exposition des humains. La LC à *L. tropica* est par ailleurs surtout fréquente en Syrie et à un moindre degré au Maroc.

L’extension géographique des leishmanioses est un risque réel en Méditerranée. Le réchauffement climatique pourrait favoriser les phlébotomes dans les pays du Nord. L’introduction de cas à *L. tropica,* anthroponotique, voire des réservoirs de *L. major,* dans ces mêmes pays, est également à évaluer et à anticiper. Dans les pays du Sud, c’est l’adaptation du mérion aux biotopes semi urbains qui pourrait provoquer des poussées épidémiques de LCZ.

# Research article: Échinococcose kystique. Diagnostic et prise en charge

**Published:** 2024-06-13

**Authors:** Mustapha BENAZZOUZ

L’échinococcose kystique, est une zoonose parasitaire cosmopolite. Elle sévit à l’état endémique dans le pourtour méditerranéen, le nord et l’est de l’Afrique, en Europe de l’Est et en Asie centrale. L’humain est un hôte intermédiaire accidentel. Le foie est l’organe le plus touché. Le diagnostic repose essentiellement sur l’échographie qui distingue, selon la classification de l’OMS, des kystes fertiles représentés par le stade CEI, CE2, des kystes en phase de transition représentés par les stades CE3a et CE3b et, enfin, des kystes inactifs représentés par les stades CE4 et CE5. Cette classification a en plus l’avantage de guider les indications thérapeutiques. La sérologie est un examen de deuxième intention après l’échographie, car elle manque de sensibilité et elle peut être négative dans les kystes CE1 et dans plus de la moitié des cas des kystes inactifs CE4/CE5. Le test de confirmation Western-Blot est plus sensible et peut être demandé en cas de contexte évocateur et de sérologie négative.

Le traitement ne s’adresse qu’aux kystes fertiles, en phase de transition (CEI, CE2, CE3a et CE3b) et aux kystes compliqués. Les kystes inactifs CE4 et CE5 ne doivent pas être traités et une simple surveillance par échographie est suffisante. Le traitement peut être médical par albendazole percutané par ponction aspiration, injection, réaspiration (PAIR), drainage percutané ou chirurgical. Pour les kystes non compliqués de stade CEI, CE3a, le traitement percutané par PAIR ou drainage percutané est le traitement de choix. Pour les kystes CE2 et CE3b, le traitement reste chirurgical dans beaucoup de centres. Des techniques de drainage modifiées visant à vider le contenu du kyste ont montré leur efficacité dans ce type de kyste.

Cependant, d’autres études prospectives comparant la chirurgie avec ces nouvelles modalités sont nécessaires. Pour les kystes CE4 et CE5 inactifs, aucun traitement ne doit être préconisé. Le traitement médical par albendazole à la dose de 10 mg/kg/j s’adresse à des kystes de petite taille (moins de 5 cm), dans les cas d’hydatidose à localisations multiples ou dans le cadre d’un traitement prophylactique pour encadrer un traitement percutané ou chirurgical. Le kyste hydatique compliqué relève le plus souvent d’un traitement chirurgical, sauf pour le traitement du kyste compliqué par fistule biliaire où un traitement endoscopique associé ou non à un traitement percutané dans le cadre d’une stratégie séquentielle peut être une excellente alternative.

# Research article: Infections par les virus Toscana et West Nile. Situation épidémiologique et réflexions de l'Italie

**Published:** 2024-06-13

**Authors:** Federico GOBBI

**Affiliations:** Département des maladies infectieuses/tropicales et microbiologie, IRCCS SCDC, Via Sempreboni 4, 37024, Negrar, Verona, Italie

Le virus Toscana (TOSV) a été isolé pour la première fois en 1971. Au cours de la période 2016-2021, 331 cas d’infections confirmées du système nerveux central par le TOSV ont été signalés au système de surveillance en Italie, avec une médiane de 56 cas par an. Le pic de notifications a eu lieu en 2018 avec 89 cas, dont 36 (40 %) sont survenus au cours du seul mois d’août.

Le virus West Nile (WNV) a été isolé pour la première fois en Italie chez des équidés en Toscane en 1998, tandis que les premiers cas humains ont été enregistrés en 2008 dans le delta du Pô. La surveillance intégrée a débuté en 2008 et a révélé la circulation des lignées 1 et 2 du WNV. Depuis 2008, des cas de WNV ont été signalés chaque année en Italie, avec des pics pour la maladie neuro-invasive de WNV de 244 (2018), 330 (2002) et 193 (2023). L’augmentation du nombre de cas en 2018 et 2002 pourrait s’expliquer par les raisons suivantes :

- 2018 : augmentation précoce et rapide présumée de la densité de la population de moustiques et peut-être amplification précoce de la transmission virale dans le cycle enzootique.

- 2022 : limitation possible du nombre d’habitats favorables à la reproduction des moustiques et à la nidification des oiseaux, avec concentration géographique des vecteurs et des hôtes, amplification virale et, par la suite, propagation de la circulation du WNV une fois que l’eau est devenue plus abondante.

# Research article: Dengue, chikungunya et autres arboviroses d'intérêt sur le pourtour méditerranéen : actualités 2023 et anticipations pour 2024

**Published:** 2024-06-13

**Authors:** Rémi CHARREL

**Affiliations:** Unité des virus émergents, Aix Marseille univ-IRDINSERM-IRBA, Marseille, France

L’actualités des dernières années a montré que les virus transmis par des arthropodes (arbovirus) et particulièrement ceux transmis par les moustiques du genre *Aedes* sont en expansion en France tant en termes de nombre de cas que de surface des territoires où ils peuvent être observés. Nous centrerons la présentation sur deux flavivirus (dengue (DENV) et Zika (ZIKV)) et un alphavirus (chikungunya (CHIKV)). Ces trois virus sont surveillés au niveau national par Santé publique France. Les tableaux cliniques ne sont pas spécifiques et le diagnostic repose donc sur des tests virologiques basés sur la détection du génome viral ou des anticorps spécifiques. Ces tests sont de plus en plus fréquemment réalisés dans les laboratoires des CHU de proximité et non plus exclusivement par le Centre national de référence des Arbovirus.

En parallèle de ce qui est observé pour les virus transmis par les moustiques du genre *Culex,* West Nile and Usutu, la transmission et la circulation de virus transmis par les moustiques du genre *Aedes* (DENV, CHIKV, ZIKV) est en pleine expansion depuis quelques années. Les raisons sont multiples et seront abordées.

En France métropolitaine, entre 2017 et 2021, 15 foyers de cas cliniques de dengue autochtones ont été décrits, couvrant 6 départements et représentant un total de 36 cas, 2 foyers de cas cliniques de Zika (6 cas) et un foyer de chikungunya (17 cas). L’année 2022 a été marquée par la description de 11 foyers de dengue autochtones couvrant 8 départements et représentant un total de 66 cas.

# Research article: Fièvre hémorragique de Crimée-Congo. Risques pour l'Europe

**Published:** 2024-06-13

**Authors:** Francesca NORMAN

**Affiliations:** National Referral Center for Tropical Medicine, Infectious Diseases Department, Ramón y Cajal Hospital, CIBERINFEC, Madrid, Spain

La liste de l’Organisation mondiale de la Santé identifiant les agents pathogènes susceptibles de provoquer une pandémie contient le virus de la fièvre hémorragique de Crimée-Congo (CCHFV). Le CCHFV peut provoquer des épidémies graves de fièvre hémorragique virale, avec une mortalité pouvant atteindre 40 %. Ce virus est endémique de zones d’Afrique, des pays des Balkans, du Moyen-Orient et d’Asie, distribution géographique qui se superpose avec celle de son principal vecteur, la tique.

Des études récentes sur les tiques, les animaux et le diagnostic de cas autochtones dans de nouvelles zones en Europe suggèrent une circulation virale plus étendue. Le diagnostic rétrospectif de cas par la détection d’anticorps spécifiques chez des patients présentant une maladie fébrile d’étiologie précédemment non spécifiée et des études de séroprévalence chez des donneurs de sang asymptomatiques (environ 1 % de séropositivité dans une étude dans l’ouest de l’Espagne) mettent en évidence la faible reconnaissance de cette maladie.

Le changement climatique, les événements météorologiques extrêmes, les oiseaux migrateurs d’Afrique porteurs de tiques infectées par le CCHFV (liées à l’établissement du virus en Espagne) et le déplacement non contrôlé de bétail et d’animaux sauvages pouvant agir comme hôtes amplificateurs, peuvent contribuer à l’émergence de cette maladie en Europe. Les premiers cas de maladies émergentes sont difficiles à reconnaître dans des zones non endémiques, soulignant la nécessité de préparation, de systèmes de surveillance et de santé publique solides, de directives actualisées pour la gestion des cas suspects et confirmés, et d’installations de laboratoire prêtes à garantir le niveau de biosécurité adéquat.

# Research article: Épidémiologie de la tuberculose résistante dans le Maghreb

**Published:** 2024-06-13

**Authors:** Sofiane ALI HALASSA

**Affiliations:** National Tuberculosis Program Manager, Professeur en pneumologie, Chef de service, Centre hospitalier universitaire (CHU) de Blida, Algérie

La tuberculose résistante est devenue un problème majeur de santé publique dans un grand nombre de pays et constitue un obstacle aux efforts qui visent à mettre fin à la tuberculose. La tuberculose multirésistante est une forme particulièrement dangereuse de tuberculose car elle est due à des bacilles résistants à au moins l’isoniazide et la rifampicine, les deux antituberculeux majeurs les plus efficaces. Elle résulte le plus souvent d’une mauvaise observance ou des traitements inadéquats. La situation de la tuberculose résistante reste un enjeu important de santé publique au Maghreb, bien que la prévalence de la résistance du *Mycobactrium tuberculosis* y soit relativement faible. Que ce soit au Maroc, en Algérie ou en Tunisie, le problème de la résistance semble contenu. Au Maroc, on a enregistré plus de 600 cas de tuberculose résistante en 2022 avec une prévalence de 1,2 % (0,43-2,6) parmi les nouveaux cas de tuberculose et 8,7 % (7,8-9,6) parmi les cas déjà traités. En Algérie, on estime à plus de 105 cas de tuberculose résistante en 2022 avec une prévalence de 2,1 % (0,45-6,2) parmi les nouveaux cas de tuberculose et 20 % (1,2-67) parmi les cas déjà traités. Quant à la Tunisie, elle a enregistré plus de 58 cas de tuberculose résistante dans la même année et une prévalence de 1,1 % (0,57-1,8) parmi les nouveaux cas, et de 34 % (20-48) parmi les cas déjà traités. La tuberculose résistante dans les pays du Maghreb, demeure un problème de santé publique malgré la baisse constante durant ces dernières décennies. Les efforts de lutte contre cette forme de la maladie doivent d’une part, viser le dépistage précoce afin de maîtriser son extension et d’autre part, assurer des schémas thérapeutiques efficaces et un approvisionnement continu en médicaments antituberculeux de qualité.

# Research article: Brucellose des ruminants : épidémiologie et stratégies de contrôle

**Published:** 2024-06-13

**Authors:** Claire PONSART

**Affiliations:** Université Paris-Est, Agence nationale de sécurité sanitaire de l’alimentation, de l’environnement et du travail (Anses), Laboratoire national de référence (LNR), Laboratoire de référence de l’Union européenne Laboratoire de référence de l’Organisation mondiale de la santé animale (OMSA) pour les brucelloses animales, Maisons-Alfort, France.

La brucellose des ruminants reste l’une des zoonoses majeures dans le bassin méditerranéen. Répandue chez les animaux domestiques et sauvages, la brucellose cause des pertes significatives dans les cheptels d’élevage et reste une préoccupation sanitaire dans de nombreuses régions du monde. Elle crée d’importants problèmes de santé publique, avec des impacts sociaux et économiques. *Brucella melitensis* représente l’espèce associée au plus grand nombre de cas humains dans la plupart des pays, en raison de contacts avec le bétail malade ou de l’ingestion de lait cru, de crème et de fromage provenant de ces animaux. Les autorités sanitaires sont appelées à mettre en place des programmes de contrôle, voire d’éradication, basés sur l’utilisation de tests et la vaccination des ruminants. Cette présentation vise à décrire les points clés permettant la mise en œuvre efficace de ces programmes. Ces programmes incluent de nombreux facteurs, à la fois sociologiques, politiques, techniques et scientifiques : sensibilisation et éducation des populations exposées et des professionnels de santé; capacité de diagnostic et de vaccination; infrastructures permettant une identification des cas; stratégies de contrôle et programmes de vaccination; coopération entre les acteurs de la santé et mise en place de systèmes de surveillance (incluant les systèmes d’information et d’identification). Les principaux schémas ayant permis de lutter contre cette maladie depuis 60 ans seront expliqués, afin de partager les expériences et de décrire les étapes nécessaires pour mettre en œuvre un contrôle efficace de cette maladie.

# Research article: Résistance aux antibiotiques dans les pays du Maghreb

**Published:** 2024-06-13

**Authors:** Adnene TOUMI

**Affiliations:** Centre hospitalier (CHU) Monastir, Tunisie

L’antibiorésistance est un problème majeur de santé publique. En effet, l’usage massif des antibiotiques a conduit à l’apparition de souches bactériennes de plus en plus résistantes. De plus, certaines bactéries multirésistantes sont isolées dans des infections communautaires, en particulier les entérobactéries sécrétrices de bêtalactamases à spectre étendu. Les experts estiment qu’en 2050, 10 millions de décès seront attribuables à l’antibiorésistance.

Les données nationales dans les pays du Maghreb montrent une augmentation croissante de la consommation des antibiotiques corrélée à l’émergence de souches bactériennes résistantes. Les taux de résistance *d’Escherichia coli* aux céphalosporines de 3^e^ génération varient entre 20 % en Tunisie et près de 45 % en Algérie. Alors que le taux de résistance de *Klebsiella pneumoniae* aux carbapénèmes ne dépassait pas 5 % au début des années 2000, il varie entre 12 et 20 % en 2019. Pour *Acinetobacter baumanii,* le taux de souches *A. baumanii* résistant à l’imipénème (ABRI) varie de 75 à 85 %. Concernant les cocci à Gram positif, le taux de pneumocoques de sensibilité diminuée à la pénicilline (PSDP) varie de 35 % au Maroc à près de 70 % en Tunisie. Enfin, la résistance du staphylocoque doré à la méticilline ne dépasse pas les 20 %. Face à cette situation alarmante, l’OMS, les sociétés savantes et les gouvernements se sont investis dans des programmes d’éducation sanitaire et d’information de la population sur les risques de mésusage des antibiotiques. Par ailleurs, une politique de bon usage des antibiotiques a été élaborée pour essayer de renverser la tendance.

# Research article: Profil épidémiologique des cas de rage humaine en Tunisie entre 2000 et 2022

**Published:** 2024-06-13

**Authors:** Amal AYACHI, Rym BEN ABDALLAH, Mariem HANDOUS

**Affiliations:** Laboratoire de la rage, Institut Pasteur de Tunis, Tunis Belvédère, Tunisie

## Introduction

En Tunisie, la rage sévit sous forme endémique et constitue un véritable problème de santé publique. Les objectifs de notre travail étaient de décrire le profil épidémio-clinique des cas de rage humaine et de rapporter les facteurs de risque liés à leur survenue.

## Patients et méthodes

Nous avons mené une étude rétrospective, descriptive et analytique des cas de rage humaine confirmés au laboratoire de la Rage de l’institut Pasteur de Tunis sur la période entre janvier 2000 et novembre 2022. Des variables temporo-spatiales, sociodémographiques, cliniques et en relation avec le contexte de l’exposition, de la post-exposition et de la riposte ont été collectées pour chaque patient.

## Résultats

Nous avons colligé 58 cas de rage humaine. Kairouan et Nabeul étaient les gouvernorats les plus touchés, avec une prédominance des zones rurales (77 %). Le maximum de cas a été recensé entre mai et novembre (74 %). La prédominance était masculine et la tranche d’âge la plus touchée était celle des patients âgés de 31 à 59 ans (30 %). La rage était principalement transmise par l’espèce canine (86 %) et par une morsure unique (55 %). Après une durée d’incubation moyenne de 60,3 jours, l’hydrophobie et les troubles du comportement étaient les signes les plus observés. Seulement 28 % des patients ont reçu une prophylaxie post-exposition et 62 % d’entre eux n’ont pas achevé la totalité du protocole.

## Conclusion

Cette étude a montré que le risque de la rage humaine est toujours présent en Tunisie et que la sensibilisation pour la prophylaxie en post-exposition est à améliorer.

# Research article: Épidémiologie de l'hydatidose au Maroc

**Published:** 2024-06-13

**Authors:** Kenza BENYOUSSEF, Souad BOUHOUT, Hafida NAOUI, Maryem IKEN, Laila BOUMHIL, Badre Eddine LMIMOUNI

**Affiliations:** Service de parasitologie et mycologie médicale; Service des maladies parasitaires, Direction de l’épidémiologie et de lutte contre les maladies; Hôpital militaire d’instruction Mohammed V, Rabat, Maroc

## Introduction

Les échinococcoses résultent du développement tissulaire de la larve d’un ténia échinocoque, parasite à l’état adulte de l›intestin grêle des canidés. Deux entités cliniques se différencient : l’hydatidose due au développement larvaire d’*Echinococcus granulosus,* et l’échinococcose alvéolaire, due à celui de la larve *d Echinococcus multilocularis.*

## Patients et méthodes

Il s’agit d’une étude rétrospective et descriptive étalée sur une période de deux ans (2021-2022) comportant 826 cas d’hydatidose. Les données ont été recueillies par le ministère de la Santé du Maroc.

## Résultats

Sur 826 cas, une prédominance des cas de sexe féminin est constatée (sex-ratio H/F = 0,92) et l’âge médian est de 42 ans. On observe une répartition inégale d’une région à l’autre avec, pour la région de Rabat-Salé-Kenitra, une prévalence de 42,7 % du total des cas (n = 352), Fès-Meknès de 24 % (n = 200), Tanger-Tétouan-Hoceima de 12 % (n = 100), Marrakech-Safi de 10 % (n = 84), Casablanca-Settat de 4,4 %(n = 37), Béni-Mellal-Khenifra de 4,4 % (n = 37) et Souss-Massa de 2 % (n = 16). Un tiers (n = 275) des patients a bénéficié d’une échographie ainsi que d’un scanner. Seulement sept patients ont fait une IRM. Dans aucun cas il n’a été réalisé une sérologie.

## Conclusion

Le foie est l’organe le plus touché (n = 503), suivi par la localisation pulmonaire (n = 155). On observe une atteinte concomitante de ces deux localisations dans 28 cas. Ces résultats montrent que l’hydatidose demeure un problème majeur de santé publique au Maroc et qu’une surveillance continue de la situation épidémiologique est essentielle afin d’espérer en réduire l’incidence.

# Research article: Potentiel danger pour la santé des espèces envahissantes de plathelminthes terrestres, en particulier comme hôtes paraténiques des Angiostrongylus

**Published:** 2024-06-13

**Authors:** Jean-Lou JUSTINE

**Affiliations:** Institut de systématique, évolution, biodiversité (ISYEB), UMR7205 CNRS, EPHE, MNHN, UPMC, Muséum national d’Histoire naturelle, 55 rue Buffon, 75005 Paris, France

## Contexte

Les plathelminthes terrestres envahissants (famille Geoplanidae) sont un nouveau composant de la faune terrestre en France et dans les pays méditerranéens (Espagne, Italie, Grèce). Plus de vingt espèces ont été recensées. L’invasion de ces espèces est spectaculaire par son étendue et son intensité : pour l’espèce *obama nungara* par exemple, les 3/4 du territoire français sont envahis et un seul jardin peut héberger des milliers d’individus. Cette invasion est récente (moins de 20 ans) et crée donc des situations nouvelles de rencontre entre ces animaux exotiques, d’une part, et les humains et les animaux domestiques, d’autre part.

## Résultats

Les plathelminthes terrestres peuvent être toxiques en cas d’ingestion du fait des composés chimiques sécrétés, en particulier leur mucus et la tétrodotoxine, un neurotoxique. Toutefois le danger potentiel le plus élevé est probablement leur rôle comme hôtes paraténiques des nématodes du genre *Angiostrongylus,* agents des angiostrongyloses humaines (méningite à éosinophiles) et animales. Plusieurs espèces sont hôtes paraténiques en région tropicale. L’espèce *obama nungara* consomme des mollusques et pourrait donc jouer un rôle similaire d’hôte paraténique, dans le contexte de l’émergence de l’angiostrongylose dans les pays méditerranéens.

## Conclusion

Ces animaux nouveaux de la faune des jardins présentent un danger potentiel pour la santé humaine et animale. Une enquête parasitologique détaillée est nécessaire pour clarifier leur rôle possible comme hôtes paraténiques dans la transmission de l’angiostrongylose.

# Research article: Pneumopathie à Covid-19 : impact pronostique à l'Unité de soins intensifs de Sfax, Tunisie

**Published:** 2024-06-13

**Authors:** Sana KHARRAT, Karama BOUCHAALA, Sabrine BRADAI, Abir DAMMAK, Rania AMMAR, Chokri BEN HAMIDA, Mabrouk BAHLOUL, Mounir BOUAZIZ

**Affiliations:** Réanimation médicale, CHU Habib Bourguiba, Sfax, Tunisie

## Introduction

Le SARS-CoV-2 est à ce jour responsable d’une mortalité importante. Plusieurs études dans la littérature se sont intéressées à la recherche des facteurs de risque de mortalité.

## Objectif

s facteurs de risque associés à la mortalité chez les patients graves atteints de Covid-19 nécessitant une hospitalisation dans les unités de soins intensifs.

## Méthode

Il s’agit d’une étude rétrospective étalée sur neuf mois. Le diagnostic d’infection à SARS COV2 a été établi par PCR.

## Résultat

Nous avons colligé 530 patients durant la période d’étude. L’âge moyen était de 58,9 ans ± 14,2. Le rapport gazométrique à l’admission était de 104,8 ± 72,3. Une atteinte pulmonaire scanographique >50 % a été observée chez 68,7 % des cas. Tous les patients hospitalisés ont bénéficié d’une oxygénothérapie dont 157 (29,6 %) ont été mis sous optiflow; 338 patients (63,8 %) ont eu des séances de ventilation non invasive et la ventilation mécanique a été nécessaire chez 234 patients (44,2 %). Le taux de mortalité a été de 48,3 %. L’analyse multivariée n’a retenu que l’âge > 65 ans (OR = 2), le taux de CRP >75 mg/l (OR = 2), le recours à la ventilation invasive (OR = 13), l’infection bactérienne nosocomiale (OR = 3) et l’hyperkaliémie (OR = 5) comme facteurs indépendants de mauvais pronostic.

## Conclusion

L’âge > 65 ans, le taux de CRP > 75 mg/l, le recours à la ventilation mécanique, la survenue d’une infection et l’hyperkaliémie étaient associés à un risque plus élevé de décès par la Covid-19. Ces résultats pourraient aider les cliniciens à identifier les patients avec un mauvais pronostic à un stade précoce.

# Research article: Le flux migratoire subsaharien en transit au Maroc vers la Méditerranée et les maladies parasitaires persistantes chez les migrants

**Published:** 2024-06-13

**Authors:** Kholoud MAAFI, Maryem IKEN, Laila BOUMHIL, Badre Eddine LMIMOUNI, Hafida NAOUI

**Affiliations:** Service parasitologie et mycologie médicale, Hôpital militaire d’instruction Mohammed V, Rabat, Maroc Faculté de médecine et de pharmacie, Université Mohamed V, Rabat, Maroc

## Introduction

De nombreux migrants en provenance d’Afrique subsaharienne traversent le Maroc avant de rejoindre la Méditerranée. L’objectif de notre étude est d’analyser les aspects épidémiologiques des pathologies parasitaires au sein de cette population.

## Patients et méthodes

Il s’agit d’une étude rétrospective descriptive sur une population de migrants subsahariens menée sur une période d’une année (2023) au sein du Laboratoire de parasitologie-mycologie de l’Hôpital militaire Mohammed V de Rabat, Maroc.

Tous les patients ont bénéficié de trois examens parasitologiques des selles plus un scotch-test anal et, pour ceux présentant une fièvre, une goutte épaisse et un frottis mince ont été réalisés.

## Résultats

Notre échantillon comprenait 124 patients (âge moyen de 28,61 ans) avec une prédominance masculine (52,8 %). Ces patients étaient principalement de nationalité sénégalaise (30,6 % des cas), suivie de la nationalité camerounaise, burkinabè et congolaise.

Le paludisme était la parasitose la plus fréquente (41 cas). *P. ovale* était l’espèce la plus retrouvée (19 cas), suivie de *P. falciparum* (17 cas) et de *P. malariae* (2 cas). Trois patients présentaient une co-infection.

Les parasitoses intestinales les plus rencontrées sont l’amœbose (4,08 %), la giardiose (4,08 %), plus rarement l’ascaridiose (2,04 %) et la strongyloïdose (un seul cas) pour laquelle la méthode de Baermann a été réalisée. Par ailleurs, nous avons recensé deux cas de loaose pour lesquels le signe d’appel était un œdème de Calabar et quatre cas de gale.

## Conclusion

Les infections demeurent les principales pathologies chez les migrants.

Leur diagnostic est devenu complexe en raison de la présentation clinique des parasitoses émergentes, ainsi que de l’impact des co-infections virales.

# Research article: Catastrophes naturelles et risque d'épidémies de maladies infectieuses transmissibles

**Published:** 2024-06-13

**Authors:** Christophe RAPP

**Affiliations:** Pôle médecine, Hôpital américain de Paris, 69 Bd Victor Hugo 92200, Neuilly sur Seine, France

## Introduction

Les catastrophes naturelles sont responsables d’une augmentation de la morbi-mortalité secondaire aux maladies infectieuses transmissibles. Le risque potentiel d’épidémies de maladies infectieuses est réel mais il est surestimé par les autorités sanitaires et les médias.

## Méthodes

Revue des principales maladies infectieuses transmissibles répertoriées au cours des catastrophes naturelles et étude de leurs déterminants

## Résultats

Les catastrophes naturelles sont responsables d’une augmentation transitoire de la morbidité et de la mortalité dans les zones sinistrées. La majorité des victimes et des décès sont en relation avec des traumatismes, fractures, écrasement de membres, brûlures ou noyades qui sont des événements de santé survenant à la phase précoce de la catastrophe.

Les principales infections sont associées à la phase tardive de la catastrophe naturelle et se classent en quatre catégories : les maladies transmises par l’eau et les aliments (majoritairement les diarrhées), les infections transmises par voie aérienne, les infections transmises par des vecteurs comme le paludisme ou la dengue et les infections de plaies et blessures. Le risque de transmission de maladies par les cadavres est négligeable. Les épidémies associées aux catastrophes naturelles sont liées aux déplacements des populations et aux effets synergiques de nombreux facteurs favorisants, tels que la promiscuité, la pénurie en eau et en assainissement.

## Conclusion

Le risque épidémique est réel mais faible lors des catastrophes naturelles. Dans l’urgence, la réponse rapide repose sur l’apport des besoins essentiels (abris, eau, assainissement, alimentation), la vaccination de masse, les soins primaires, la lutte antivectorielle et la mise en place d’un système de surveillance et d’alerte épidémiologique.

# Research article: La Méditerranée, porte de l'espoir vers l'Europe

**Published:** 2024-06-13

**Authors:** Francesco CASTELLI

**Affiliations:** Professeur de maladies infectieuses, chaire UNESCO « Training and empowering human resources for health development in resource-limited countries », Université de Brescia, Italie

Bien que la migration soit un phénomène ancien qui a accompagné l’humanité depuis ses débuts, ces dernières décennies, la Méditerranée est devenue l’une des routes les plus périlleuses pour les migrants cherchant à entrer en Europe, et l’un des itinéraires migratoires les plus meurtriers à l’échelle mondiale. Depuis 2014, 28 806 migrants ont perdu la vie en Méditerranée (source : Organisation internationale pour les migrations, OIM). L’interaction complexe de facteurs socio-économiques, d’instabilité politique et de conflits dans les régions entourant la Méditerranée (déterminants de la migration) constitue une force motrice derrière cette tendance migratoire. La quête de meilleures opportunités, de sécurité et d’espoir pousse les individus à entreprendre ces voyages périlleux et à affronter les dangers de la mer. La migration non sécurisée expose les migrants à des risques considérables pour leur santé, notamment l’exposition à des conditions difficiles, à la violence et à un manque d’accès aux soins de santé de base. Les disparités de santé parmi les migrants, résultant des dynamiques d’inégalité sociale et de marginalisation dans les pays de destination et de transit, impactent non seulement le bien-être individuel, mais entravent également les progrès vers les objectifs mondiaux de santé d’un point de vue plus large de la santé publique. Un accès limité aux services essentiels de santé publique mine l’autonomie, l’intégration et l’autonomisation des individus, conduisant à une augmentation de la stigmatisation et de l’exclusion. Les conséquences sur la santé mentale, l’exposition aux maladies infectieuses, les préoccupations liées à la santé maternelle et la manque de continuité des soins de santé chroniques font partie des défis de santé pressants auxquels sont confrontés les migrants lors de ces voyages périlleux.

Pour relever ces défis, il est essentiel d’intégrer les besoins sanitaires des populations migrantes dans les politiques sanitaires internationales et nationales. L’urgence d’inclure les migrants et leurs besoins spécifiques en matière de santé dans les politiques de santé se reflète dans l’Agenda 2030 des Nations Unies, qui stipule clairement que «personne ne devrait être laissé pour compte» et appelle les États à répondre aux besoins et aux vulnérabilités des migrants. Cela implique le renforcement de l’infrastructure sanitaire, la fourniture de services accessibles et culturellement sensibles, et la mise en œuvre de politiques qui protègent le bien-être des migrants. Cela souligne l’importance de reconnaître les défis de santé uniques auxquels sont confrontés les migrants et souligne l’engagement envers des politiques de santé inclusives et équitables à l’échelle mondiale et nationale.

# Research article: État de santé des personnes migrantes originaires d'Afrique

**Published:** 2024-06-13

**Authors:** Nicolas VIGNIER

**Affiliations:** Service de maladies infectieuses et tropicales, Hôpital Avicenne, AP-HP, Bobigny, France

Les personnes migrantes originaires d’Afrique représentent la population immigrée en France chez laquelle sont le plus souvent diagnostiquées des pathologies infectieuses et tropicales, en lien avec l’épidémiologie de leur pays d’origine. En effet, la plus grande partie de l’Afrique subsaharienne est située en zone intertropicale où sévissent encore des maladies transmissibles historiques (tuberculose maladie, maladies diarrhéiques, pyodermites, paludisme, infection par le VIH-1 et 2, etc.) et/ou (ré)émergentes (fièvres hémorragiques virales, Mpox, poliomyélite dérivée d’une souche vaccinale, etc.). Les personnes migrantes d’Afrique ont emprunté des parcours migratoires différents, parfois difficiles quand le départ est lié à des menaces pesant sur la personne et quand le trajet passait par des pays n’assurant pas la sécurité des personnes migrantes. Ces traumatismes multiples ne sont pas sans conséquences sur la santé mentale et physique des personnes présentant souvent des troubles psychiatriques complexes (syndromes anxiodépressifs et syndrome de stress post traumatique) susceptibles de s’aggraver quand les conditions d’accueil sont mauvaises, ce qui est malheureusement fréquent.

La migration d’Afrique concerne aussi des personnes plus aisées chez qui ces pathologies sont moins fréquentes mais chez qui des enjeux de santé publique existent cependant. Leurs pathologies est le reflet de la transition sanitaire en marche en Afrique avec un accroissement des pathologies non transmissibles telles que les maladies cardiovasculaires, le diabète et les cancers. D’autres spécificités liées à la présence d’allèles particuliers sont à connaître, en particulier le risque d’hémoglobinopathies.

La proposition d’un bilan de santé systématique après l’arrivée est ainsi recommandée pour dépister précocement les pathologies non connues.

# Research article: État de santé des personnes migrantes originaires d'Amérique latine

**Published:** 2024-06-13

**Authors:** Marta GONZALEZ-SANZ

On estime que plus de 4,6 millions de migrants latino-américains résident en Europe. La majorité (2,2 millions) vit en Espagne, l’autre moitié dans la région méditerranéenne de l’Italie et de la France.

Les migrants d’Amérique latine peuvent souffrir de maladies endémiques dans leur pays, peu connues dans notre milieu, qui représentent pourtant un défi diagnostic.

La maladie de Chagas, une maladie parasitaire négligée provoquée par *Trypanosoma cruzi.* La transmission est principalement vectorielle dans les pays endémiques, tandis qu’en Europe, la seule forme de transmission est verticale, de la mère à l’enfant durant la grossesse ou lors de l’accouchement. La plupart des patients atteints de la maladie de Chagas sont asymptomatiques; s’ils ne reçoivent pas de traitement, la maladie peut progresser, entraînant une atteinte cardiaque ou gastro-intestinale. Il faut développer une meilleure connaissance de la maladie de Chagas dans les zones non endémiques afin de garantir que la population à risque ait accès aux tests diagnostiques et aux traitements pour ainsi prévenir une transmission ultérieure et éviter la morbidité causée par le parasite.

Une autre maladie tropicale négligée à prendre en compte est la strongyloïdose, présente dans le monde entier mais assez fréquente en Amérique latine, souvent en cas de co-infection par le virus HTLV (Human T Lymphotropic virus). À l’instar de la maladie de Chagas, l’infection produite par Strongyloides stercoralis est souvent asymptomatique mais peut provoquer une maladie très grave appelée syndrome d’hyperinfestation si le patient devient immunodéprimé et qu’aucun traitement n’est fourni.

# Research article: Les maladies infectieuses parmi les réfugiés syriens au Liban

**Published:** 2024-06-13

**Authors:** Jacques CHOUCAIR

Le déplacement d’environ un million et demi de réfugiés syriens dans des camps de réfugiés ou en dehors sur le territoire libanais depuis le début de la guerre civile syrienne a eu des conséquences sur différents plans dont le médical.

D’abord, le niveau socio-économique de ces réfugiés est bas : 90 % vivent dans la pauvreté et 58 % des enfants sont sans éducation. Les aisés ont émigré en Europe ou Amérique du Nord.

Parmi les maladies transmissibles des réfugiés, on compte les infections virales, bactériennes, mycosiques et parasitaires. Parmi les affections virales, on cite le VIH, les virus des hépatites A, B et C, le SarsCov2 et la grippe, les oreillons, la poliomyélite ainsi que les diarrhées liquides aiguës. Dans les affections bactériennes, on compte la tuberculose (et surtout la tuberculose résistante), le choléra (du fait de la naissance d’une épidémie nationale à partir d’un cas syrien soigné au Nord Liban), les infections respiratoires (surtout les pneumonies bactériennes) et les infections cutanées (essentiellement à streptocoques, staphylocoques et pseudomonas souvent résistants suite à un abus d’antibiothérapie) ainsi que des mycoses cutanées. Enfin, les affections parasitaires avec des « mini épidémies » de gale (16 243 cas), d’infection par les poux (19 696 cas) et des cas de leishmaniose (2 787 cas dont 99,8 % de syriens) traités en 2015.

La situation actuellement empire avec l’arrivée illégale d’un plus grand nombre de réfugiés et le déclin continu de l’économie libanaise, qui se traduit par la diminution d’aide financière aux réfugiés et la corruption parmi les instances qui en sont responsables.

# Research article: Violences sexuelles, précarité et migration

**Published:** 2024-06-13

**Authors:** Claire TANTET

**Affiliations:** Dispositif Parcours, Service des maladies infectieuses et tropicales de l’hôpital Avicenne, Bobigny, France; Hôpital Bichat, paris, France

La France, pays d’immigration de longue date, compte 7 millions d’immigrés en 2022, soit 10,3 % de l’ensemble de la population. Un immigré est défini comme une personne née étrangère à l’étranger et résidant en France. Répartis de manière inégale sur le territoire, plus d’un tiers des immigrés (37 %) réside en Île-de-France, tandis qu’ils représentent près d’un tiers de la population de Seine-Saint-Denis.

Un grand nombre d’immigrés cumule les vulnérabilités sociales. Dans le pays d’origine, sur le trajet migratoire et dans le pays d’accueil, ils sont exposés à des violences institutionnelles, physiques, sexuelles et psychologiques. À l’arrivée dans le pays hôte, les difficultés socio-économiques s’ajoutent : précarités alimentaire, financière, de l’hébergement, isolement social et relationnel. L’accès au système de santé devient alors une problématique cruciale. Les difficultés sont liées à un défaut de couverture médicale, une méconnaissance des droits et des procédures, ainsi qu’à des discriminations de la part des professionnels de santé. À ces barrières s’ajoutent la complexité des démarches administratives et le durcissement des politiques d’accueil.

Les femmes immigrées, en particulier celles en situation irrégulière, se retrouvent particulièrement vulnérables, subissant des discriminations et des violences liées au genre qui dégradent leur état de santé et entravent leur accès aux soins. Les mutilations génitales, ainsi que l’ensemble des violences liées au genre, sont des motifs fréquemment évoqués par les femmes comme cause d’exil et de demande d’asile. Également documentés lors du trajet migratoire, les viols sont 18 fois plus fréquents en France chez les demandeuses d’asile que dans la population générale (4,8 % *vs.* 0,26 %) et le risque de violences sexuelles est neuf fois plus élevé (26,3 % *vs.* 2,9 %). Par ailleurs, 35 % des personnes exilées infectées par le VIH ont été contaminées en France, en lien avec la précarité et l’exposition au risque. Ce cumul des fragilités, liées à l’exil, aux violences et aux conditions d’accueil, justifie la nécessité d’un accueil spécifique pour accompagner les exilés. C’est dans ce contexte qu’a été créé le dispositif Parcours en 2017 au sein de deux services d’infectiologie du nord de Paris, en France. Il est inspiré du modèle « One-stop Centre », qui propose aux victimes de violences sexuelles et de genre des soins médicaux, un soutien psychosocial et une assistance juridique au sein d’un lieu unique.

# Research article: Troubles psychiatriques et experience migratoire

**Published:** 2024-06-13

**Authors:** Andrea TORTELLI

**Affiliations:** Consultation d’accompagnement psychiatrique et Social pour migrants en Île-de-France (CAPSYS), Pôle Psychiatrie précarité, Groupe hospitalier universitaire Paris psychiatrie & neurosciences (GHU Paris), INSERM Paris, France

Le risque augmenté des troubles psychiatriques en population migrante est observé depuis les grandes vagues migratoires de la fin du XIX^e^ siècle vers les Amériques. Une première hypothèse s’est portée sur la possibilité que les personnes étaient déjà atteintes de troubles psychiatriques avant la migration. Des études ultérieures montrent plutôt une association à un cumul d’expositions à des facteurs de risque de troubles psychiatriques avant, pendant et après la migration. Cette surexposition nous aide à mieux comprendre les déterminants sociaux de la santé : des personnes ayant subi une migration forcée et non programmée (guerre, persécution) présentent un risque plus important que des migrants venus pour des raisons de travail ou de regroupement familial. Enfin, la nouvelle vague migratoire à la suite des conflits au Moyen-Orient et en Afrique mettent en lumière une population migrante particulièrement vulnérable sur le plan psychique, mettant à l’épreuve les systèmes de santé des pays d’accueil. Nous allons discuter de la prévalence et de l’incidence de troubles psychiatriques chez cette population, et des facteurs de risques associés, notamment en France.

# Research article: Épidémiologie et prise en charge des mutilations génitales féminines

**Published:** 2024-06-13

**Authors:** Sébastien MADZOU

**Affiliations:** Centre hospitalier universitaire (CHU) d’Angers, Pôle femme-mère-enfant, 4 rue de Larrey, 49 933 Angers Cedex 9, France

Les mutilations génitales féminines (MGF) sont définies par l’Organisation mondiale de la santé (OMS) comme « toutes les interventions incluant l’ablation partielle ou totale des organes génitaux externes de la femme ou toute autre lésion des organes génitaux féminins, qui sont pratiquées pour des raisons non médicales ». Le taux de MGF dans le monde concerne 200 millions de filles et de femmes, qui sont autant de victimes en vie actuellement. De plus, trois millions de jeunes filles seraient menacées par ces pratiques tous les ans, selon le Fonds des Nations unies pour les activités en matière de population (UNFPA).

Ces pratiques sont répandues dans environ 31 pays répartis sur les trois continents.

La prise en charge chirurgicale permet : la réhabilitation de la femme sur les plans juridique, anatomique, sensoriel, sexuel et psychologique; la réduction de la douleur; la prévention des complications obstétricales; la lutte contre les mutilations.

# Research article: Nuisances entomologiques en consultation. Identifier le responsable des piqûres en 15 mn

**Published:** 2024-06-13

**Authors:** Pascal DELAUNAY

**Affiliations:** Service de parasitologie-mycologie. Centre hospitalier universitaire (CHU) de Nice, Hôpital de l’Archet. 151, route Saint Antoine de Ginestière. CS 23079. 06202 Nice cedex 3, France

## Introduction

L’entomologie médicale ne s’apprend que peu en santé humaine. Elle est enseignée certes pour le « risque vectoriel » : paludisme, lyme, dengue … mais l’insecte n’est pas le cœur du problème lors d’une consultation d’infectiologie. Pour une problématique de « nuisance », hormis la gale, les pédiculoses et les punaises de lit, les autres insectes sont quasi inconnus par le monde des soignants. Or ne pas identifier l’insecte responsable, c’est ne pas poser de diagnostic, dispenser des conseils inefficaces, couteux et toxiques qui augmentent l’infestation du logement, et qui peut aboutir à des intoxications gravissimes ou des suicides.

## Matériel et méthode

Dans un cadre de consultation « 15 mn », il est possible, d’identifier les six insectes majeurs des nuisances (sarcoptes-poux-punaises-puces-pyemotesdermanyssus) et de dispenser des conseils de lutte sans insecticide.

Quatre points sont à apprendre :

1. Connaître le cycle biologique de ces six arthropodes.

2. Il existe six types ou objectifs de consultations pour le patient et/ou le soignant.

3. L’aspect de la « piqûre » ne permet pas d’identifier un insecte.

4. L’identification de l’insecte se fait en posant trois questions clés :

4a Date de l’apparition des signes et évolution depuis cette celle-ci 4b Type d’environnement dans lequel vit le patient : ville, campagne, chat, chiens pigeons.

4c Localisation des « piqûres » : partie couverte-découverte, haut-bas du corps …

## Résultat

En associant ces quatre points et leurs résultats, vous identifierez les six arthropodes majeurs.

## Conclusion

Vous pourrez proposer des conseils de lutte, efficaces, sécurisés et apaisants pour le logement du patient.

# Research article: La malnutrition, un fléau mondial à plusieurs facettes

**Published:** 2024-06-13

**Authors:** Jalila EL ATI

**Affiliations:** Service études et planification. Institut national de nutrition et de technologie alimentaire, 11, rue Jebel Lakhdar Bab Saadoun, 1007, Tunis, Tunisie.

## Introduction

La malnutrition, définie comme un état pathologique résultant d’un déséquilibre nutritionnel, se présente sous diverses formes, allant de la sous-nutrition à la surnutrition, affectant tant les pays développés que ceux en développement. La malnutrition a des conséquences dévastatrices sur la santé à l’échelle mondiale.

## Méthodes

Une recherche de la littérature et des bases de données pertinentes a été effectuée dans les bases de données PubMed et Google Scholar.

## Résultats

La malnutrition touche diverses populations de manière différente et peutêtre influencée négativement par des normes culturelles, des habitudes, des déterminants structurels, des expositions différentielles et des lacunes dans le système de santé. Les facteurs socio-économiques, tels que la pauvreté et l’accès limité à une alimentation nutritive, jouent un rôle central dans la prévalence de la sous-nutrition. Parallèlement, la surnutrition est souvent associée à des habitudes alimentaires malsaines, à une urbanisation rapide et à des changements de mode de vie. Il est de plus en plus important de considérer la sousnutrition et la surnutrition non pas comme deux problèmes distincts traités en vase clos, mais comme des questions interconnectées partageant des processus physiopathologiques communs qui, ensemble, contribuent au double fardeau de la malnutrition.

## Conclusion

Les efforts pour atténuer la malnutrition nécessitent une approche holistique. Des programmes de sensibilisation, des politiques nutritionnelles efficaces, l’accès à une alimentation équilibrée et des soins de santé adaptés constituent des éléments clés pour lutter contre ce fléau. La recherche sur les mécanismes sous-jacents de la malnutrition contribue à élaborer des stratégies ciblées et efficaces.

# Research article: Impact des pollutions marines sur la santé humaine

**Published:** 2024-06-13

**Authors:** Patrick RAMPAL

**Affiliations:** Centre hospitalier Princesse Grace, 1 Av. Pasteur, 98000 Monaco, Principauté de Monaco

La pollution marine est définie comme l’introduction directe ou indirecte de déchets, de substances, ou d’énergies, qui entraîne des effets nuisibles pour les écosystèmes marins, avec pour conséquence, une altération de la qualité des eaux, un appauvrissement de la biodiversité et des risques pour la santé humaine.

Quatre-vingts pour cent de la pollution marine est d’origine terrestre, les polluants pouvant être transportés par le ruissellement des eaux, mais aussi par le vent.

On distingue la pollution générée par les déchets aquatiques de celle produite par les polluants organiques et l’eutrophication.

La pollution par les déchets aquatiques est dominée par la pollution par le plastique. Chaque année, 10 millions de tonnes de plastique entrent dans les océans. Une partie est ingérée par les animaux marins et peut avoir des effets mécaniques sur la survie des espèces. La majeure partie du plastique sera dégradée en microplastiques par l’effet des rayons ultra-violets et sera absorbée puis concentrée dans la chaîne alimentaire marine.

Les effets délétères des microplastiques sur la santé humaine sont bien établis sur le plan microbiologique où les plastiques dégradés assurent le transport d’agents bactériens dans le milieu communiquant océanique.

Par ailleurs, les plastiques participent à la pollution organique des océans. Dans la composition du plastique, interviennent de nombreux « polluants organiques persistants » à la toxicité avérée qui résistent aux dégradations biologiques, se déplacent sur de très longues distances, se bioaccumulent dans la chaine alimentaire et sont des perturbateurs endocriniens et des agents carcinogènes avérés.

Parmi les polluants organiques, il faut aussi mentionner les hydrocarbures, et les métaux lourds comme le mercure, source de retard du développement lors des expositions *in utero* et de troubles neurologiques chez les adultes.

Enfin, il faut mentionner l’eutrophisation : l’activité humaine (agriculture intensive, rejet des eaux usées) en libérant des nutriments (essentiellement azote et phosphore) en zones côtières, associée au réchauffement des océans, entraine un déficit de biodiversité et la prolifération d’espèces animales, végétales et microbiennes pouvant être source de la prolifération d’algues toxiques ou d’épidémies bactériennes.

Un être humain pourrait ingérer environ 5 grammes de plastique chaque semaine, soit l’équivalent de la quantité de microplastiques contenue dans une carte de crédit.

# Research article: L'envenimation scorpionique en Afrique du Nord. Épidémiologie, physiopathologie et prise en charge.

**Published:** 2024-06-13

**Authors:** Mabrouk BAHLOUL, Karama BOUCHAALA, Mounir BOUAZIZ

**Affiliations:** Réanimation médicale, Centre hospitalier universitaire (CHU) Habib Bourguiba Sfax Rte El Ain km 0.5 3029 Sfax, Tunisie

L’envenimation scorpionique (ES) est un accident relativement fréquent dans les zones tropicales et subtropicales des cinq continents, en particulier en Amérique latine, en Afrique du Nord, au Moyen Orient et en Inde. Particulièrement fréquentes en Afrique du Nord, les piqûres de scorpion peuvent constituer une urgence médicale grave, notamment chez le jeune enfant.

La plupart des ES (80 % à 90 %) se caractérisent par une douleur locale intense et isolée (stade 1) qui persiste jusqu’à 24 heures. Bien que très rarement observées, (moins de 1 % de l’ensemble des piqûres par scorpion), les formes sévères d’envenimation scorpionique (stade 3) restent redoutables du fait qu’elles peuvent être responsables d’une défaillance cardio-respiratoire et peuvent mettre en jeu le pronostic vital. Ces formes graves correspondent à deux manifestations d’insuffisances cardiaques aiguës : choc et/ou œdème pulmonaire cardiogénique; et/ou à des manifestations neurologiques : coma ou convulsions. L’atteinte du système cardio-vasculaire est la plus étudiée. Trois hypothèses sont évoquées pour expliquer la dysfonction cardiaque. Il s’agit de la myocardite adrénergique (décharge massive de catécholamines), la myocardite toxinique (effet direct du venin sur les membranes des cellules myocardiques) et l’ischémie myocardique. La corrélation entre le jeune âge et la sévérité des manifestations cliniques après l’ES a été bien établie. De plus, plusieurs études ont démontré que l’envenimation scorpionique grave est associée à une réaction inflammatoire systémique (SIRS) pouvant expliquer les manifestations systémiques souvent observées chez les patients graves (stade 2 ou 3). Sur le plan thérapeutique, l’indication de l’antivenin et le traitement symptomatique dépendent de la gravité clinique de l’envenimation. Le traitement des formes graves comporte, en plus de de l’antivenin spécifique, des moyens symptomatiques : oxygénothérapie, assistance ventilatoire pour l’œdème aigu du poumon et perfusion de dobutamine dans l’état de choc cardiogénique.

# Research article: Faire face à la crise humanitaire : la réponse médicale de SOS Méditerranée

**Published:** 2024-06-13

**Authors:** Marylène BIQUEZ

**Affiliations:** SOS Méditerranée, Marseille, France

## Introduction

SOS Méditerranée (SOS) est une organisation humanitaire maritime qui se consacre à la crise qui se déroule en mer Méditerranée. Elle s’efforce de fournir une assistance immédiate aux personnes en détresse en mer, en exploitant le navire de recherche et de sauvetage, Océan Viking.

## Dispositif médical

L’équipe est composée d’un chef d’équipe médicale, d’un médecin, d’un infirmier et d’une sage-femme. Ils disposent d’un espace pour les soins d’urgence et les consultations médicales, et d’une zone d’observation de trois lits. L’équipement médical est complet. Si nécessaire, les patients sont référés aux autorités sanitaires à terre. L’équipe anticipe un niveau élevé d’acuité, se tenant prête à faire face à des urgences telles que la réanimation après une noyade, la gestion des traumatismes et d’autres crises médicales imprévues.

## Résultats

Les problèmes de santé les plus courants chez les survivants sont liés à leurs expériences en mer, notamment les brûlures de carburant, l’hypothermie, l’hypoglycémie, la déshydratation et l’épuisement. Le manque d’hygiène et les conditions insalubres pendant leur voyage ou dans les centres de détention en Libye entraînent des infections cutanées, des plaies chroniques, des maladies diarrhéiques, des infections des voies respiratoires et l’exacerbation de maladies chroniques. Au-delà des affections physiques, les personnes présentent souvent des blessures liées à la violence et des problèmes de santé mentale tels que l’anxiété, la dépression, la psychose et le syndrome de stress post-traumatique.

## Conclusion

Cette présentation présente les multiples facettes des besoins de santé des personnes dans le contexte de la migration méditerranéenne et la manière dont l’organisation y répond.

# Research article: Les facteurs prédictifs d'insuffisance rénale aiguës suite à l'intoxication par la paraphénylène-diamine

**Published:** 2024-06-13

**Authors:** Sabrine BRADAI, Sana KHARRAT, Karama BOUCHAALA, Rihab BOUBTANE, Najeh BACCOUCHE, Rania AMMAR, Olfa TURKI, Mabrouk BAHLOUL, Chokri BENHAMIDA, Mounir BOUAZIZ

**Affiliations:** Service de réanimation polyvalente Centre hospitalier universitaire (CHU) Habib Bourguiba, Sfax, Tunisie Sabrine

## Introduction

L’intoxication systémique par la paraphénylène-diamine (PPD), un produit utilisé pour teindre les cheveux, peut entraîner une insuffisance rénale aiguë (IRA). L’objectif de notre travail est d’analyser le profil clinique et paraclinique de cette complication et de déterminer les facteurs prédictifs de sa survenue.

## Matériel et méthode

Étude rétrospective, descriptive et analytique, menée dans notre service de réanimation polyvalente au CHU Habib Bourguiba de Sfax, sur une période de 17 ans (2006-2023). Nous avons inclus tous les patients victimes d’intoxication au PPD.

## Résultats

Nous avons retenu 16 patients. À l’admission, une rhabdomyolyse a été constatée chez 15 patients (94 %) avec un taux moyen de CPK à 30 985 ± 53 412 UI/l et un taux moyen d’ASAT à 873,5 ± 1 247,3 UI/l. Une IRA a été constatée chez sept patients (44 %), chez qui le taux moyen d’urée était à 10,2 ± 4,9 mmol/l et le taux moyen de créatinine était à 121 ± 36,9 pmol/l. L’aspect des urines étaient porto chez 14 patients (81 %). Deux patients ont bénéficié d’une dialyse. Les facteurs prédictifs de survenue d’IRA étaient les scores SAPSII (p = 0,001), APACHEII (p = 0,015), SOFA (p = 0,001), la fréquence cardiaque (p=0,007), le taux d’urée (p = 0,017), le taux de créatinine (p = 0,001), le pH (p = 0,009), le taux de HCO3-(p = 0,037). L’IRA est un facteur de mauvais pronostic (p = 0,009), avec un taux de mortalité de 86 %.

## Conclusion

L’IRA au cours de l’intoxication par la PPD est fréquente. Il s’agit d’une néphropathie tubulaire par précipitation de myoglobine. Elle est de mauvais pronostic. Le meilleur traitement est préventif, se basant sur le remplissage vasculaire et l’alcalinisation précoces.

# Research article: Envenimation scorpionique chez les enfants : manifestations cliniques et facteurs pronostiques

**Published:** 2024-06-13

**Authors:** Karama BOUCHAALA, Sabrine BRADAI, Sana KHARRAT, Chokri BEN HAMIDA, Mabrouk BAHLOUL, Mounir BOUAZIZ

**Affiliations:** Unité de soins intensifs, Centre hospitalier universitaire (CHU) Habib Bourguiba, Sfax, Tunisie

## Introduction et objectif

Étudier les caractéristiques épidémiologiques et cliniques et préciser les facteurs pronostiques des envenimations scorpioniques sévères chez l’enfant.

## Méthodologies

Étude rétrospective sur 13 ans. Les patients ont été classés en deux stades : grade II sans défaillances vitales et grade III avec défaillances vitales (choc cardiogénique et/ou œdème pulmonaire ou manifestation neurologique sévère : coma et/ou convulsions).

## Résultats

Durant la période de l’étude, 685 patients ont été inclus. Il y avait 558 patients (81,5 %) dans le groupe grade III et 127 patients (18,5 %) dans le groupe grade II. Dans cette étude, 434 patients (63,4 %) avaient un œdème pulmonaire et 80 patients avaient un choc cardiogénique; des manifestations neurologiques ont été observées chez 580 patients (84,7 %), 555 patients (81 %) ont développé un syndrome de réponse inflammatoire systémique (SIRS) et 552 patients (80,6 %) ont développé une défaillance multiviscérale. À la fin du séjour en réanimation, l’évolution a été marquée par le décès de 61 patients (8,9 %). Une analyse multivariée a révélé que les facteurs suivants étaient corrélés à un mauvais pronostic : coma avec un score de Glasgow < 8/15 (odds ratio [OR] = 1,3), œdème pulmonaire (OR = 2,3) et choc cardiogénique (OR = 1,7).

## Conclusion

L’envenimation scorpionique présente un sérieux problème de santé publique. La présence d’une défaillance cardiocirculatoire ou neurologique est associée à un mauvais pronostic.

# Research article: De la médecine tropicale (coloniale) à la décolonisation de la santé globale

**Published:** 2024-06-13

**Authors:** Marco ALBONICO, Beatrice BARDA, Manuela PETINO, Maria José CALDÉS PINILLA

**Affiliations:** PhD, President FESTMIH, CD SIMET, Via San Domenico 3, 10122 Torino, Italie

## Introduction

Le concept de décolonisation de la santé globale découle des disparités de pouvoir persistantes dans la relation qui existe entre les praticiens et les chercheurs en santé globale dans ce que nous appelons le « Nord » et le « Sud ». Le concept de décolonisation de la santé globale est intrinsèquement associé à la coopération et à la recherche en matière de santé, toutes deux financées par l’aide publique au développement et créées à l’origine dans le cadre des relations coloniales. Bien qu’elle ait pris différentes formes au cours de l’histoire, telles que la médecine coloniale, la médecine tropicale et la santé internationale, la santé globale reste intrinsèquement liée aux formes actuelles de colonialisme.

## Méthodes

Nous avons effectué une analyse de la littérature actuelle sur le sujet en examinant les aspects de la situation actuelle d’inégalité d’accès aux connaissances et aux programmes de recherche entre les professionnels du Nord et du Sud, ainsi que dans la mise en œuvre des projets de coopération sanitaire pour le développement. Pour mieux comprendre les perceptions de l’aide et des projets de santé internationaux, nous avons élaboré un questionnaire qui a été soumis à différents acteurs locaux opérant sur le terrain au Kenya, en Tanzanie, au Mozambique et au Sénégal.

## Résultats et conclusion

Les résultats du questionnaire montrent que les projets de coopération dépendent de l’aide extérieure mais que les priorités sont principalement définies par les communautés et les acteurs locaux. La plupart d’entre eux sont bien intégrés à la culture et aux traditions des bénéficiaires. Cependant, une discrimination raciale et financière importante demeure. La transparence, la prise de décisions indépendantes sur les politiques de santé, l’inclusion, le respect, l’équité, des incitations financières équitables sont autant de solutions possibles pour décoloniser l’aide humanitaire au développement.

# Research article: N° 1 La tuberculose vertébrale dans l'est algérien : quelle particularité ?

**Published:** 2024-06-13

**Authors:** Nabila KALLA, Ouanassa HAMOUDA, Asma ABDESSAMAD, Achouak BELHADI, Amina AYADI, Ibtihal AOUADJ, Zohra MERZOUGUI, Souhila AOUIDANE, Nora RIGHI

**Affiliations:** Établissement public hospitalier (EPH) de Batna, Faculté de médecine, Université Batna 2, Algérie

## Introduction

En Algérie, La tuberculose est l’une des pathologies infectieuses les plus endémiques. La localisation vertébrale est la plus répandue. Le but de cette étude est de décrire les particularités épidémio-cliniques et radiologiques des spondylodiscites tuberculeuses.

## Patients et méthodes

Il s’agit d’une étude descriptive, rétrospective, portée sur les dossiers des patients présentant une spondylodiscite tuberculeuse, hospitalisés à l’établissement public hospitalier de Batna. Les dossiers ont été colligés sur une période de deux ans. Le diagnostic a été affirmé par l’isolement des *Mycobacterium tuberculosis* (BK) et/ou par l’examen anatomopathologique.

## Résultat

Nous avons colligé 10 patients. Il s’agit de cinq femmes et de cinq hommes. Le délai moyen du diagnostic était de 134,22 jours. Le principal signe révélateur était les rachialgies. La fièvre était constatée chez 70 %. L’étage lombaire était le plus touché. Les explorations radiologiques avaient noté un aspect en miroir chez 40 % des patients, une lyse de l’angle vertébral antérieur chez 50 %, une image de reconstruction osseuse chez 30 % et une ostéolyse de l’arc postérieur dans 20 % des cas. Les principales complications étaient : une épidurite (30 %), un abcès des parties molles paravertébrales (30 %), un abcès de psoas (50 %). Le BK a été isolé seulement chez quatre patients. L’évolution était favorable chez huit cas. Les séquelles étaient constatées chez deux malades.

## Conclusion

La spondylodiscite tuberculeuse est fréquente. Son évolution est généralement favorable en cas de diagnostic précoce, néanmoins des complications neurologiques ont été rapportées.

# Research article: N° 2 Quel profil de la tuberculose avant, pendant, et après la pandémie Covid-19 ?

**Published:** 2024-06-13

**Authors:** Hajar EL FOUAR, Mohammed ER-REJRRAGI, Khadija DANAOUI, Rania EL FARGANI, Wiam AIT DRISS, Malika IDALENE, Noura TASSI

**Affiliations:** Service des maladies infectieuses, Centre hospitalier universitaire (CHU) Mohammed VI, Marrakech, Maroc

## Introduction

La pandémie Covid-19 a perturbé énormément la prise en charge des autres maladies lors du confinement total, parmi lesquelles la tuberculose.

## Matériels et méthodes

Étude rétrospective de janvier 2017 à janvier 2023 au Service des maladies infectieuses CHU Mohamed VI de Marrakech, en incluant tous les cas de tuberculose confirmés ou probables. Ces cas ont été classés en période pré-Covid 2017-2019, per-Covid 2020-2021 et post-Covid 2022-Mars 2023

## Résultats (voir tableau)

 

## Conclusion

Dans notre étude, l’incidence de la tuberculose a nettement augmenté au cours de la période post-Covid par rapport à la période pré-Covid et per-Covid. On a noté également un retard considérable du diagnostic de la tuberculose au cours de la période post-Covid expliquant la prédominance des formes multifocales et graves et l’augmentation du taux de décès. Notre étude montre ainsi l’impact négatif de la pandémie sur le diagnostic et le pronostic de la tuberculose, qui est dû essentiellement à la difficulté d’accès aux soins.

# Research article: N° 3 La tuberculose neuroméningée à propos d'une série à Batna

**Published:** 2024-06-13

**Authors:** Nora RIGHI, Loubna RAHMOUNE, Hanene BENALDJIA, Ooum Kelthoum MANSOURI, Bruno LINA

**Affiliations:** Service des maladies infectieuses, Établissement public hospitalier de Batna, Algérie

## Introduction

La tuberculose neuro-méningée est rare : elle concerne environ 1 à 2 % de l’ensemble des cas de tuberculose et 5 % des cas de tuberculose extra pulmonaire. Son diagnostic est rendu difficile par le manque de spécificité des éléments cliniques et para cliniques.

## Matériels et méthodes

Une étude prospective, longitudinale et exhaustive avait porté sur des patients âgés de plus de 28 jours ayant des symptômes évocateurs d’encéphalite répondant aux critères d’inclusion et d’exclusion établies par SPILF durant la période du 1^er^ avril 2012 au 31 août 2015.

## Résultats

Nous avons pu colliger 141 patients, parmi eux 65/141 cas (46,1 %) était d’origine bactérienne, dont 30 cas de tuberculose a été détecté; 21 confirmé / probable. La médiane d’âge était de 30,5 (0-83), prédominance féminine 12/18, 23,3 % de comorbidités, fréquence de déficits moteurs (86,7 %) et des troubles psychiatriques (26,8 %), l’atteinte respiratoire était présente dans 60 % des cas, la pleiocytose était présente chez 22/25 (73,4 %). L’hyperalbuminorachie pour 66,7 %, et l’hypoglycorachie pour (46,7 %); 17,6 % était initialement admis dans un service de soins intensif, le décès a été identifié chez 46,6 % avec 10 % des séquelles.

## Conclusion

Dans notre étude outre les virus, des bactéries (surtout intracellulaires), la plus fréquente reste la tuberculose. Le pronostic est conditionné par la précocité du traitement.

**Table:** 

Période	Pré-Covid (2017-2019)	Per-Covid (2020-2021)	Post-Covid (2022-mars 2023)
**Âge moyen**	38,5	40	37
**Sex-ratio (H/F)**	1,46	2,52	1,22
**Localisation %**			
pulmonaire	38,5	26,8	21,4
extra-pulmonaire	32,2	28,3	20,4
pulmonaire et extra-pulmonaire	29,1	44,7	58,1
**Tb Confirmé (GeneXpert-Culture) %**	64,8	65,6	65,3
**Tb Probable (arguments cliniques) %**	34,9	34,3	34,6
**Délai diagnostique (semaines)**	11,9	10,6	14,6
**Statut VIH %**	51	47,7	63,2
**Évolution (décès) %**	6,2	16,4	18,3
**Total %**	96	67	98
**Incidence cas/an %**	33	33,5	78,4

# Research article: N° 4 La tuberculose à l'ère de la Covid-19 : à propos de 82 cas

**Published:** 2024-06-13

**Authors:** Hajar EL FOUAR, Khadija DANAOUI, Rania EL FARGANI, Wiam AIT DRISS, Malika IDALENE, Noura TASSI

**Affiliations:** Service des maladies infectieuses, Centre hospitalier universitaire (CHU) Mohammed VI, Marrakech, Maroc

## Introduction

La Covid-19 a eu d’importantes répercussions sur la prise en charge d’autres pathologies dont la tuberculose (TB) qui existent encore au Maroc à l’état endémique.

## Matériels et méthodes

Étude menée au sein du Service des maladies infectieuses du CHU Mohamed VI entre mars 2020 et mai 2022, en étudiant les caractéristiques des tuberculeux hospitalisés dans notre service pendant cette période.

## Résultats

L’étude a porté sur 82 patients tuberculeux. Leur âge moyen était de 33,5 ans, avec une prédominance masculine et un sexe ratio de 2,03. Le délai moyen entre le début des symptômes et la consultation était de 17 semaines. Les différentes localisations étaient : tuberculose multifocale pour 44 %; tuberculose miliaire pour 14 %; tuberculose pulmonaire pour 14 %; tuberculose neuroméningée pour 11 %; spondylodiscite tuberculeuse pour 9 %; tuberculose abdominale pour 6 %; et un cas de tuberculose ganglionnaire. La tuberculose était confirmée par gène expert chez 49 patients, par culture chez trois patients, mais elle a été retenue devant un faisceau d’arguments chez 30 patients. Une diminution du nombre des tuberculeux hospitalisés a été constatée durant la période du confinement à raison de cinq patients soit une prévalence 6,09 %, en raison peut être des mesures de confinement face à la Covid-19.

## Conclusion

Les défis posés par la pandémie de Covid-19 sont nombreux, notamment en ce qui concerne le maintien des actions de lutte contre la tuberculose. Nous espérons que ce travail contribuera à soutenir de nouvelles études et la mise en place de politiques publiques visant à faire face aux deux maladies.

# Research article: N° 5 Dépistage de la tuberculose chez les migrants mineurs non accompagnés au Centre de lutte antituberculeuse des Alpes Maritimes en 2023

**Published:** 2024-06-13

**Authors:** Frédéric VANDENBOS, Flore PETIT, Victoria BUNGER, Julie BERNARDESCHI, Christelle PIERRAT, Marie Laure RAYNAL, Karine RISSO

**Affiliations:** Centre de lutte antituberculeuse (CLAT 06), 30 voie Romaine, 06001 Nice Cedex 1, France

## Introduction

Nous rapportons l’expérience du dépistage de la tuberculose chez les migrants mineurs non accompagnés (MNA) au Centre de lutte antituberculeuse (CLAT) des Alpes-Maritimes en 2023.

## Patients et méthodes

Le dépistage de la tuberculose a consisté en un interrogatoire infirmier, une radiographie du thorax et la réalisation d’un QuantiFERON^®^.

## Résultats

En 2023, 625 MNA ont été dépistés au CLAT 06. Ils venaient tous d’Afrique. Parmi ceux-ci, 6 MNA avaient une tuberculose maladie (4 formes pulmonaires, une forme ganglionnaire et une forme osseuse) et 143 une infection tuberculeuse latente (ITL). L’incidence de la tuberculose maladie (TM) était de 960/100 000 et la prévalence de l’ITL de 23 % chez ces MNA en 2023. Parmi ces 149 MNA infectés (TM et ITL), il y avait 139 hommes pour 10 femmes et l’âge moyen était de 15,2 ans. Le pays d’origine était majoritairement la Guinée (43,5 %) et la Côte d’Ivoire (36,5 %). Deux des MNA avec TM ainsi que 27 des MNA avec ITL ont fugué et/ou ont été déclarés majeurs entrainant une rupture de prise en charge.

## Conclusion

L’incidence de la tuberculose chez les MNA dans les Alpes-Maritimes est très élevée. Le dépistage de la tuberculose chez les migrants reste donc un enjeu majeur de santé publique.

# Research article: N° 6 Tuberculose ostéoarticulaire : aspects épidémiocliniques à Monastir (Tunisie)

**Published:** 2024-06-13

**Authors:** Amina SKHIRI, Meriem ABDELJELIL, Wafa MARRAKCHI, Foued BEN ROMDHANE, Abir AOUAM, Chawki LOUSSAIEF, Adnene TOUMI, Mohamed CHAKROUN

**Affiliations:** Service des maladies infectieuses, Centre hospitalier universitaire (CHU) Fattouma Bourguiba, Monastir, Tunisie

## Introduction

La tuberculose ostéoarticulaire (TOA) constitue 2 à 5 % de l’ensemble des localisations tuberculeuses. Les signes cliniques et radiographiques de la TOA sont polymorphes. L’objectif de notre étude était d’étudier les caractéristiques épidémiologiques, cliniques et thérapeutiques de la TOA.

## Patients et méthodes

Étude rétrospective portant sur les patients hospitalisés pour TOA dans le service des maladies infectieuses du CHU Fattouma Bourguiba de Monastir-Tunisie entre 2010 et 2022. Les données étaient analysées à l’aide du logiciel SPSS.

## Résultats

Vingt cas de TOA étaient colligés avec un sex-ratio (H/F = 0,3). L’âge moyen était de 49 ans ± 16,3. La localisation discovertébrale était la plus fréquente (n = 16,8 %), suivie par des localisations strictement osseuses ou articulaires dans deux cas (10 %) chacune. Une altération de l’état général et des sueurs nocturnes ont été notées dans cinq cas (25 %) chacune. Les autres signes cliniques associés étaient principalement représentés par : les douleurs rachidiennes (n = 13,7 %), une impotence fonctionnelle (n = 6,3 %) et des manifestations neurologiques (n = 5,3 %). Une biopsie osseuse ou disco-vertébrale a été réalisée dans la majorité des cas (n = 16,8 %). Le diagnostic était retenu sur les données de l’examen anatomo-pathologique dans 14 cas (70 %) et sur le résultat de la PCR BK dans un seul cas (5 %). Une quadrithérapie antituberculeuse a été démarrée chez tous les patients.

## Conclusion

Dans notre série, la localisation disco-vertébrale représente la localisation la plus fréquente au cours de la TOA. Le diagnostic repose sur un faisceau d’arguments épidémiocliniques, bactériologiques et anatomo-pathologiques.

# Research article: N° 7 Effets secondaires des antituberculeux lors de la tuberculose extra pulmonaire (TEP) à Mostaganem, en l'absence d'alternative. Quelles solutions ?

**Published:** 2024-06-13

**Authors:** Samira BESTAOUI

**Affiliations:** Centre hospitalier universitaire (CHU) de Mostaganem, Algérie

## Introduction

Malgré la large couverture vaccinale par le BCG, la tuberculose reste un problème de santé publique. La TEP devient de plus en plus fréquente, elle peut devancer la forme pulmonaire à l’heure actuelle. De nombreuses localisations sont possibles, même les plus anecdotiques. L’évolution insidieuse et le polymorphisme clinique sont à l’origine du retard de diagnostic et associés à des durées de traitement plus longues induisant des effets secondaires difficiles à juguler.

## Objectif

Évaluer les différentes localisations de la TEP, apprécier la durée du traitement spécifique et énumérer ses effets secondaires.

## Patients et méthodes

Une étude rétrospective a été menée à Mostaganem sur une période de 24 mois allant de janvier 2022 à décembre 2023, colligeant 302 cas de TEP.

## Résultats

Cinquante-neuf patients ont été inclus, dont une majorité de femmes (52,6 %). L’âge médian était de 43,37 ans; 67 malades avaient des antécédents de tuberculose. Cinq patients étaient coinfectés par le VIH. Les localisations retrouvées étaient les suivantes : ganglionnaires (28,2 %), osseuses (17,9 %), neuroméningées (10,6 %), oculaires (uvéite, choriorétinite : 4,3 %), collections abcédées (9 %); 25,5 % des patients avaient plus de deux localisations. La durée du traitement antituberculeux était variable, de 9 à 18 mois. Ce traitement était interrompu chez 10 % des patients pour une intolérance clinique : éruption, fièvre, trouble digestifs, encéphalopathies hépatiques et biologique : cytolyse, TP effondré. Dans deux cas, une contreindication absolue à la réinitialisation des antituberculeux était signalée. Des schémas alternatifs ont été proposés. Cinq adénites tuberculeuses ont récidivé après un traitement court de six mois. Deux décès ont été constatés.

## Conclusion

Cette étude reflète que la prise en charge thérapeutique constitue un obstacle pour le clinicien en raison de la longue durée et des effets secondaires du traitement ainsi que du manque de consensus nationaux pour la prise en charge d’une pathologie qui devient de plus en plus fréquente dans nos zones.

# Research article: N° 8 Étude pilote du traitement de courte durée de la tuberculose multirésistante au Centre hospitalier régional Georges Rawiri de Lambaréné, Gabon

**Published:** 2024-06-13

**Authors:** Madiou DIALLO, Dieudonné EYAMAME, Élie Gide ROSSATANGA, Désiré MBA OBIANG, Éric YONGONGO

**Affiliations:** Service des maladies tropicales, Centre hospitalier régional Georges RAWIRI de Lambaréné, Gabon

## Introduction

Au Gabon, une étude pilote de l’évaluation du régime court du traitement de tuberculose multirésistante connu sous le nom « schéma thérapeutique de Bengladesh », a été menée au CHR de Lambaréné en collaboration avec le Centre de recherche médical Lambaréné, avant son approbation par l’OMS en 2016.

## Méthodes

Étude observationnelle menée de septembre 2015 à septembre 2016. Une formation des agents de santé ainsi qu’une information et éducation communautaires ont été menées. Les patients atteints de tuberculose multirésistante confirmée par Gene Xpert ont reçu les médicaments antituberculeux de 2^e^ intention durant neuf mois. Des frottis et des cultures d’expectorations ont été effectués mensuellement. Les événements indésirables ont été surveillés quotidiennement.

## Résultats

Onze patients ont été traités pour une tuberculose multirésistante dans le cadre du programme pilote de traitement court. Tous étaient séronégatifs et présentaient une altération de l’état général, des lésions pulmonaires étendues. Le taux de conversion des cultures d’expectorations était de 64 % après quatre mois de traitement. Trois patients ont développé une perte auditive et une éruption cutanée passagère. Sur 11 patients, 7 (63,6 %) se sont améliorés sur le plan clinique et bactériologique; un échec thérapeutique (9,1 %),deux (18,2 %) décédés et un perdu de vue (9,1 %).

## Conclusion

Nos données pionnières sur le traitement de tuberculose multirésistante au Gabon, avaient montré qu’un régime de neuf mois peut aboutir à une négativité précoce des cultures et une amélioration clinique. Une surveillance des événements indésirables et des soins continus sont essentiels au succès.

# Research article: N° 9 Les nouveaux aspects cliniques de la brucellose dans l'est algérien

**Published:** 2024-06-13

**Authors:** Nabila KALLA, Amel BARKAT, Aicha BELAGOUNE, Nora RIGHI

**Affiliations:** Etablissement public hospitalier (EPH) de Batna, Faculté de médecine, Université Batna 2, Algérie

## Introduction

Dans notre pays, la brucellose est considérée comme l’une des zoonoses les plus répandues. La brucellose s’individualise par son polymorphisme clinique. Les formes focalisées en font toute la gravité, mettant parfois en jeu le pronostic vital. L’objectif de cette étude est de décrire le profil actuel des formes cliniques de la brucellose dans notre région.

## Patients et méthodes

Il s’agit d’une étude transversale menée au Service des maladies infectieuses (unité d’hospitalisation homme) incluant tous les patients hospitalisés pour une brucellose. Les dossiers ont été colligés sur une période de deux ans. Le diagnostic de la brucellose est retenu par l’isolement des brucelles et/ou par le sérodiagnostic de Wright.

## Résultats

Durant ces deux dernières années, 27 patients ont été hospitalisés pour une brucellose. L’âge moyen est de 47 ans. 20 cas (74,07 %) ont une habitation en milieu rural. 64 % des cas ont une notion de contact avec les animaux infectés et 73 % des patients ont rapporté la notion de consommation de produits laitiers non pasteurisés. Cliniquement, huit (29,6 %) patients avaient présenté une brucellose aiguë septicémique. Les formes focalisées ont été : une neurobrucellose (3,7 %), une orchite subaiguë (3,7 %), une endocardite subaiguë (3,7 %), une spondylodiscite (48,1 %), une sacro-iliite (7,4 %). L’association de ces deux dernières localisations a été remarquée chez un seul malade. Un cas de brucellose compliquée d’un syndrome d’activation macrophagique a été noté chez un patient immunocompétent.

## Conclusion

La brucellose vertébrale continue d’être le principal foyer lésionnel de brucellose dans notre région. D’où l’intérêt d’y penser devant toute rachialgie fébrile.

# Research article: N° 10 Neurobrucellose : aspects cliniques et radiologiques

**Published:** 2024-06-13

**Authors:** Meryem DJEMA, Fatima Zahra CHABANI, Rachida BELHADJ AISSA, Meryem MEBARKI, Fadila DJANI, Anissa TEBANI, Karima SAIGHI, Nora AIT KACI, Hanane TAICHA, Youssef SELLAMI, Mohamed YOUSFI

**Affiliations:** Service des maladies infectieuses, Établissement public hospitalier (EPH) de Boufarik, Blida, Algérie

## Introduction

La brucellose est une zoonose ubiquitaire touchant essentiellement le bassin méditerranéen et le Moyen-Orient où elle pose encore un véritable problème de santé publique, due à des bactéries du genre Brucella. L’atteinte neurologique reste rare (<10 %), mais peut s’observer au cours de la phase aiguë ou chronique de la maladie.

## Patients et méthodes

Étude rétrospective descriptive portant sur neuf dossiers de patients hospitalisés pour brucellose neurologique au service des maladies infectieuses de l’EPH Boufarik du 12 septembre 2018 au 30 décembre 2023.

## Résultats

Pour 9 cas de neurobrucellose (NB), un sex-ratio de 2, un âge moyen de 41,44 ans. 75 % de nos patients sont des agriculteurs, qui consomment des produits laitiers non pasteurisés (PLNP) dans 100 % des cas. L’atteinte neurologique était au premier plan dans 95 % des cas (méningoencéphalite; 78 %; méningite : 12,5 %; atteinte radiculaire : 11 %). Les signes cliniques prédominants étaient : céphalées (89 %), vertiges et fièvre (55 %), diplopie (22 %), surdité de perception (22 %), troubles de l’équilibre et troubles psychiques (11 %). Le sérodiagnostic de Wright est positif dans (sang 100 %/LCS 87,5 %), l’atteinte méningée est retrouvée dans 89 % des cas. Les aspects radiologiques par résonance magnétique (IRM) allaient d’un aspect normal, méningo-encéphalite abc voire compression médullaire.

## Traitement

Rifampicine, sulfamethoxazole-trimethoprime, gentamycine; durée moyenne neuf mois, une seule l’intervention chirurgicale (compression médullaire). Évolution favorable dans 90 % des cas. Normalisation du LCS dans 100 % des cas dès le 3^e^ mois, disparition des lésions radiologiques entre 3 et 12 mois de traitement.

## Conclusion

Le polymorphisme clinique de la neurobrucellose fait de cette maladie un diagnostic différentiel de nombreuses affections neurologiques.

# Research article: N° 12 Profil épidémiologique et clinique des dermohypodermites aiguës au Service des maladies infectieuses de Batna Algérie

**Published:** 2024-06-13

**Authors:** Nora RIGHI, Asma ARAMI, Shoumayssa OUNIS, Ilhem MERBOUHI, Loubna RAHMOUNE

**Affiliations:** Service des maladies infectieuses, Établissement public hospitalier (EPH) de Batna, Algérie

## Introduction

Les dermohypodermites aiguës bactériennes sont classées anatomiquement selon la profondeur de l’atteinte en dermohypodermite non nécrosante (DHDNN) et nécrosante. Elle constitue un motif fréquent de consultation en infectiologie.

## Patients et méthodes

Il s’agit d’une étude rétrospective et descriptive, qui avait porté sur les dossiers des patients présentant une dermohypodermite hospitalisés au Service des maladies infectieuses de Batna, durant une période de 15 mois entre janvier 2022 et mars 2023.

## Résultats

Durant la période d’étude, 21 cas de dermohypodermite ont été collectés. L’âge moyen des patients était de 56,81 avec un sexratio de 1,6; 57,1 % des patients avaient un IMC supérieur à 30 kg/m^2^. 76,2 % des patients avaient au moins un facteur de risque à savoir une artériopathie (19 %), une insuffisance veineuse (4,8 %), un lymphœdème (9,2 %), et du diabète (33,2 %). La notion de prise d’antiinflamatoires non stéroïdiens (AINS) a été rapportée chez 38,1 % des patients et 23,8 % avaient un antécédent de DHDNN. L’atteinte était unilatérale dans 90,5 % des cas, avec un siège situé surtout au niveau du membre inferieur (85,7 %). Les signes de gravité ont été manifestes dans 23,8 % des cas. La porte d’entrée est retrouvée pour 80,9 %. La fasciite nécrosante, l’abcédation et l’atteinte osseuse ont été les principales complications et ont été observées respectivement chez 14 %, 23 %, 9 %des patients.

# Research article: N° 14 Évaluation du profil actuel de la résistance aux antibiotiques des souches *d'Escherichia coli* au Centre universitaire hospitalier (CHU) de Batna

**Published:** 2024-06-13

**Authors:** Nabila KALLA, Ouanassa HAMOUDA, Lina NOUI, Amani MELIZI, Souhila AOUIDANE, Zohra MERZOUGUI, Sana BELKHALFA, Fatima BOUZIANE

**Affiliations:** Faculté de médecine, Université Batna 2, Batna, Algérie

## Introduction-objectif

L’émergence des bactéries multirésistantes aux antibiotiques représente un enjeu majeur de santé publique. *Escherichia coli* est l’une des entérobactéries les plus répandues. L’objectif de cette étude est d’évaluer le profil actuel de la résistance aux antibiotiques des souches d’E. *coli.*

## Patients et méthodes

Étude descriptive prospective incluant les dossiers des patients hospitalisés au CHU de Batna durant la période allant de janvier 2023 à mars 2023 qui présentaient une infection documentée avec isolement des souches d’E. *coli* dans les différents prélèvements. Les données épidémiologiques et microbiologiques ont été récupérées à travers les dossiers des patients.

## Résultats

Nous avons colligé 85 patients ayant une infection documentée à *E. coli*. Il s’agit de 53 hommes et 32 femmes avec un âge moyen de 49 ans. Les prélèvements de pus et des sérosités représentent 62,4 % des sites d’isolement des bactéries, alors que les prélèvements urinaires, sanguins (hémocultures) représentent respectivement 27,1 % et 5,9 % des prélèvements.

Sur 85 souches d’E. *coli* qui ont été identifiées, 20 (23,5 %) isolats étaient des bactéries multirésistantes (BMR) dont la majorité des souches étaient productrices de bêtalactamases à spectre élargi (BLSE). Parmi ces *Escherichia coli* BLSE, 40 % sont résistantes à la ciprofloxacine et 50 % au bactrim, mais restent sensible en particulier à l’amikacine, gentamicine, fosfomycine et à l’imipenème dans 100 %.

## Conclusion

Au cours de ces dernières années, nos hôpitaux ont été confrontés à une situation inquiétante caractérisée par la présence croissante de bactéries résistantes aux antibiotiques. Ces données soulignent la gravité de la situation et l’importance de réagir de manière adéquate

# Research article: N° 15 La description des infections urinaires à bactéries multirésistantes à Batna Algérie

**Published:** 2024-06-13

**Authors:** Nora RIGHI, Sara BOUBKER, Soumia BOUHLASSA, Aïcha BELAGOUN

**Affiliations:** Service des maladies infectieuses, Établissement public hospitalier (EPH), Batna, Algérie

## Introduction

Les voies urinaires sont le 2^e^ site d’infections bactériennes après le poumon. La résistance aux antibiotiques des bactéries responsables des infections urinaires communautaires est en nette augmentation.

## Matériel et méthode

Il s’agit d’une étude rétrospective et descriptive portant sur les dossiers des patients hospitalisés dans le Service des maladies infectieuses de Batna et présentant une infection urinaire documentée à bactérie multirésistante (BMR). Les dossiers ont été colligés sur une période allant du trois mars 2022 au 15 décembre 2022.

## Résultat

Nous avons pu colliger 11 cas d’infections urinaires à BMR. L’âge moyen de nos patients était de 59,09 ± 24,7. L’origine communautaire a été notée dans 90,9 % des cas et l’origine nosocomiale dans 9,10 %. La pyélonéphrite aiguë, la cystite, la prostatite ont été les principales formes cliniques et ont été observées respectivement chez 27,3 %, 54,54 %, 18,2 % des patients. Les germes responsables sont en majorité des entérobactéries dont les souches étaient productrices de BLSE.

*E. coli* a été le germe le plus fréquemment retrouvé à l’ECBU 55,6 %. Deux isolats étaient des bactéries hautement résistantes (BHR) de type EPC (entérobactéries productrices de carbapénèmases) dans 100 % des cas. Le traitement était basé sur imipinem+/gentamicine ou amikacine chez 81,8 % des patients, et sur colistine chez 18,19 % des malades. L’évolution était favorable chez 100 % des cas.

## Conclusion

À travers cette étude nous avons constaté que l’origine communautaire des infections urinaires à bactéries multirésistantes est en nette augmentation.

# Research article: N° 17 La comparaison de l'encéphalite et méningoencéphalite entre le sujet âgé et le sujet jeune

**Published:** 2024-06-13

**Authors:** Nora RIGHI, Warda BOUCETTA, L. BENSAKHRIA, Daniel FLORET, Bruno LINA

**Affiliations:** Service des maladies infectieuses, Établissement public hospitalier (EPH), Batna, Algérie

## Introduction

Les personnes âgées constituent un groupe de sujets à risque vis-à-vis de la plupart des pathologies infectieuses qui représentent la 3^e^ cause de mortalité primaire après 65 ans et la première cause de mortalité accessoire.

## Méthodes

Sur une période de trois ans, une étude prospective longitudinale et exhaustive avait porté sur des cas d’encéphalite répondant aux critères d’inclusion et d’exclusion établie par la Société de pathologie infectieuse de langue française (SPILF). Deux groupes étaient constitués (groupe A, moins de 65 ans et groupe B, 65 ans et plus) et comparés sur plan épidémiologique, clinique et évolutif.

## Résultats

Sur 141 dossiers qui répondaient aux critères : 38 étaient exclus (âge < 15 ans); 92 (65,2 %) dans le groupe A, avec 44 (47,8 %) cas d’étiologies virales confirmées et/ou probables, 31 (33,7 %) d’étiologies bactériennes confirmées et/ou probables, 3 (3,3 %) d’étiologies parasitaires (3,3 %). Onze (7,8 %) dans le groupe B, avec huit cas d’étiologies virales confirmées et/ou probables, deux cas d’étiologies bactériennes et un cas d’étiologie parasitaire. 72,7 % présentaient une comorbidité dans le groupe B contre 27,2 % dans le groupe A. Le cytomégalovirus (CMV), l’herpès simplex (HSV), et l’Epstein Barr virus (EBV) étaient les trois agents pathogènes les plus fréquents dans le groupe B *vs* le BK, EBV, HSV … Listeria dans le groupe A. La mortalité est plus importante dans le groupe B (72,7 %) contre 28,3 % pour la population jeune.

## Conclusion

Cette étude nous a permis de souligner que le diagnostic reste difficile puisque la confusion, les signes de localisation ou les crises comitiales sont des manifestations fréquentes et peu spécifiques.

# Research article: N° 18 Donovanose contractée au Maroc : à propos d'un cas

**Published:** 2024-06-13

**Authors:** Wiam AIT DRISS, Malika IDALENE, Noura TASSI

**Affiliations:** Service des maladies infectieuses, Centre hospitalier universitaire (CHU) Mohammed VI, Marrakech, Maroc

## Introduction

La donovanose est une infection bactérienne à *Klebsiella granulomatis,* de localisation principalement génitale et de transmission essentiellement sexuelle, prédominant en zone tropicale. Nous en rapportons une observation où l’infection a été contractée au Maroc.

## Observation

Un homme de 73 ans s’est présenté pour une masse ulcérée géante inguinale étendue à la cuisse droite, associée à un nodule du bras droit évoluant depuis six ans dans un contexte de conservation de l’état général et d’apyrexie, sans notion de voyage récent à l’étranger. Une biopsie cutanée a été réalisée montrant un aspect d’inflammation chronique granulomateuse ulcérée faisant évoquer un granulome inguinal (donovanose). Un traitement par azithromycine orale, à raison de 1 g le premier jour, puis 500 mg par jour, a été mis en place en prise continue jusqu’à résolution clinique. L’évolution clinique a été spectaculaire, avec une régression presque totale des ulcérations inguinales et une régression partielle du nodule au niveau du bras.

## Conclusion

Cette observation rappelle que la donovanose peut survenir au Maroc, d’où l’intérêt des mesures préventives efficaces contre les infections sexuellement transmissibles (IST).

# Research article: N° 19 Résurgence de la diphtérie au sud de l'Algérie en 2023

**Published:** 2024-06-13

**Authors:** Benyoucef CHACHOU, Nassima ACHOUR, Hichem GHAOUI, Abderrezak BOUAMRA, Abdelmalek MATARI, Hassiba TALI MAAMAR, Faicel HAOUES, Nabila BENAMROUCHE, Ali KEHAL, Mohamed ZIDAN

**Affiliations:** Service des maladies infectieuses, Hôpital universitaire de Blida, Blida, Algérie

## Introduction

Le début de l’année 2023 a été marqué par l’apparition d’un foyer épidémique de diphtérie au sud de l’Algérie, et cela malgré un contrôle par la vaccination obligatoire et généralisée. L’objectif de notre étude a été de décrire les aspects épidémiocliniques des cas de diphtérie.

## Patients et méthodes

Une étude rétrospective et descriptive a été réalisée à l’hôpital de Bordj Badji Mokhtar durant une période de trois mois (Février- Avril 2023). Le diagnostic a été établi selon les données épidemio-cliniques et les résultats bactériologiques du laboratoire de bactériologie médicale de l’Institut Pasteur d’Alger, Algérie.

## Résultats

Quarante-six cas ont été inclus. L’âge médian était de 15,5 ans (2-39 ans), le sexe ratio de 1,4. Le pic épidémique a été recensé à la 2^e^ et la 3^e^ semaine de l’épidémie. Le statut vaccinal était défaillant chez la totalité des malades. Les pseudomembranes grisblanc étaient présentes dans 100 % des cas. La présence du gène *tox* a été retrouvée par PCR dans 30 % des prélèvements; la culture a été positive au biotype *C. diphteriae mitis* dans 20 % des prélèvements. Aucun décès n’a été enregistré.

## Conclusion

La résurgence de la diphtérie au sud de l’Algérie en 2023 est probablement liée à l’importance des mouvements des populations migrantes des pays du Sahel et à la défaillance de la vaccination. La vaccination urgente de masse chez les ressortissants étrangers des pays voisins et le renforcement de la surveillance épidémiologique sont les meilleurs garants de la riposte antiépidémique.

# Research article: N° 21 Tétanos : aspects épidémiologiques et facteurs pronostiques dans le Service des maladies infectieuses du Centre hospitalier universitaire (CHU) de Brazzaville de 2018-2023

**Published:** 2024-06-13

**Authors:** Bienvenu Rolland OSSIBI IBARA, Bobo MPASSY MOHEMA, Tatia ADOUA DOUKAGA, Martin EKAT, Ella ANGONGA PABOTA, Princesse BENDETT LEBAHO, Alain Maxime MOUANGA

**Affiliations:** 1 Faculté des sciences de la santé, Université Marien Ngouabi, 1 Avenue des premiers jeux africains, Diata, Brazzaville, République du Congo; 2 Service des maladies infectieuses (SMI), CHU de Brazzaville, Congo; 3 Service de psychiatrie, CHU de Brazzaville, République du Congo

## Objectif

Contribuer à l’amélioration de la prise en charge du tétanos au CHU de Brazzaville.

## Patients et méthode

Il s’agissait d’une étude transversale à visée descriptive et analytique des cas de tétanos admis dans le SMI entre janvier 2018 et août 2023, soit 68 mois.

## Résultats

Cent-deux patients étaient inclus (5,5 % des admissions), d’âge moyen de 31 ±18,5 (2-78) ans, de sexe masculin prédominant (n = 86; 84,3 %, sex-ratio de 5,4). Ils étaient élèves/étudiants (n = 41; 40,2 %), ouvriers/artisans (n = 27; 5,8 %), célibataires (83,3 %), résidaient surtout en ville (98 %) et avaient un niveau d’instruction de niveau secondaire pour la plupart (n = 64). Le statut vaccinal était absent dans 61,7 % des cas et incomplet dans 32,4 % des cas. Une infection par le VIH était retrouvée chez 6,9 % des patients. Le délai moyen de consultation était de 94,57 ±5 (48-336) heures. Les principaux signes d’admission étaient le trismus (n = 62; 60,8 %) et la dysphagie (n = 13; 12,7 %). Les portes d’entrées étaient tégumentaires (n = 75; 73,5 %) et intramusculaires (n = 8; 7,8 %). Le score de Dakar était compris entre 4 et 6 chez 19 patients (18,6 %) et celui de Mollaret IIIB chez 16 patients (15,7 %). Le sérum antitétanique était administré par voie intramusculaire chez 38 patients (39,6 %) ou sous occipitale chez trois patients (2,9 %). La durée moyenne d’hospitalisation était supérieure ou égale à 15 jours pour 39 patients (38,2 %) et la létalité était de 42,2 % (n = 43). Les facteurs pronostiques étaient l’âge (IC : 1,3-12,9; p = 0,009), la profession (p = 0,03) le diabète (p = 0,007), le score de Dakar de 4-6 (IC : 4-86,6; p = 0,00003) et celui de Mollaret de IIIA (IC : 3,6-78,7; p = 0,0009).

## Conclusion

Le tétanos reste une maladie fréquente au CHU de Brazzaville, en lien avec l’insuffisance de vaccination au sein de la population juvénile. Les facteurs pronostiques sont identiques à ceux retrouvés dans la littérature. La létalité reste élevée. La prévention reste la seule mesure efficace et passe par la vaccination.

# Research article: N° 22 Facteurs associés à l'infection néonatale précoce d'origine bactérienne au Centre hospitalier universitaire (CHU) de Brazzaville

**Published:** 2024-06-13

**Authors:** Nanikaly MOYEN, Jeanne GAMBOMI, Nelly NGAKENGNI, Bienvenu Gladmich BITSINDOU, Bienvenu Rolland OSSIBI IBARA, Gaston EKOUYA BOWASSA

**Affiliations:** 1 Université Marien Ngouabi, Faculté des sciences de la Santé, République du Congo; 2 Service bactériologie-immunologie-virologie CHU, Brazzaville, République du Congo; 3 Service de néonatologie, CHU, Brazzaville, République du Congo; 4 Service des maladies infectieuses, CHU, Brazzaville, République du Congo

## Introduction

L’infection néonatale (IN) bactérienne résulte de la colonisation de l’organisme du nouveau-né par un germe. On classe les IN en infections précoces ou infections materno-fœtales (IMF) diagnostiquées (<3 jours) et les infections néonatales tardives (INT) entre J4 et J28. Dans les pays en voie de développement, les infections néonatales restent un problème de santé publique. L’objectif de l’étude était de déterminer les facteurs associés à la survenue de l’infection néonatale bactérienne au CHU de Brazzaville.

## Patients et méthode

Il s’agit d’une étude transversale analytique (avril à septembre 2023). Les nouveau-nés suspects d’infection néonatale hospitalisés étaient inclus. Les données cliniques, socio-démographiques (nouveau-nés et mères) et obstétricales étaient recueillies. Les analyses statistiques étaient faites à l’aide du logiciel IBM SPSS statistique 25.0.

Le seuil de significativité (p) était de 0,05. Les variables avec p à 20 % était mises dans le modèle univarié. L’identification bactérienne était faite par hémoculture.

## Résultats

Un total de 118 nouveau-nés était recueilli. L’IN bactérienne était confirmée chez 79 (66,9 %) des nouveau-nés, avec une IMF dans 59,5 % des cas et une INT dans 40,5 % des cas. Les variables avec un p de seuil de significativité étaient : le niveau d’instruction secondaire (p = 0,03), le nombre de CPN (consultation prénatale) « <4 » (p = 0,01), le type de lait « maternisé » (p = 0,16), le mode d’allaitement « à la sonde » (p = 0,02). Le fait de faire moins de CPN était associé à la survenue de l’IN bactérienne chez le nouveau (p = 0,02). Plusieurs études en Afrique rapportent des taux élevés d’IMF similaires à ceux trouvés dans notre étude.

## Conclusion

L’IN reste encore un problème de santé publique dans les pays en développement.

# Research article: N° 23 Les patients vivant avec le VIH perdus de vue à l'ère de la Covid 19. Expérience du Service des maladies infectieuses du Centre hospitalier universitaire (CHU) Mohammed VI de Marrakech

**Published:** 2024-06-13

**Authors:** Wiam AIT DRISS, Malika IDALENE, Noura TASSI

**Affiliations:** Service des maladies infectieuses, CHU Mohammed VI, Marrakech, Maroc

## Introduction

La pandémie de Covid 19 ainsi que les mesures de confinement ont entraîné une crise sanitaire exceptionnelle pouvant potentiellement impacter le suivi des patients ayant une pathologie chronique, dont les patients vivant avec le VIH (PvVIH).

## Patients et méthodes

Étude rétrospective descriptive et analytique, portant sur 30 Pv-VIH ayant arrêté leur suivi (perdus de vue) au Service des maladies infectieuses du CHU Mohammed VI, à Marrakech entre juin 2020 et décembre 2021.

## Résultats

Le nombre de patients perdus de vue était de 30. L’âge moyen était de 39,25 ans. Une prédominance masculine a été notée avec 65 % d’hommes. La durée moyenne de perte de vue était de 15,35 mois, avec des extrêmes allant de 6 mois à 2 ans. La moyenne des CD4 était de 467 cellules/mm^3^. La charge virale était disponible seulement chez 60 % des patients avec une moyenne de 129 605 copies/ml. Sur le plan thérapeutique, 85 % des patients étaient sous trithérapie antirétrovirale alors que 15 % des cas n’étaient pas encore sous traitement. Les causes d’arrêt de suivi identifiées étaient sociales dans 40 % des cas, psychologiques dans 17 % des cas, liées à la pandémie Covid 19 dans 36 % des cas, ou autres chez 7 % des cas. L’évolution était marquée par la reprise du suivi par 54 % des patients avec l’enregistrement de complications dans 35 % des cas.

## Conclusion

Notre étude a mis en évidence la grande ampleur du problème des perdus de vue chez les PvVIH durant la pandémie de Covid 19 et en a évalué les déterminants.

# Research article: N° 24 Contribution des communautaires dans l'amélioration de l'observance des enfants et adolescents suivis au Centre de traitement ambulatoire (CTA) Donka : expérience de la fondation Espoir de Guinée

**Published:** 2024-06-13

**Authors:** Aïssatou Lamarana BAH, Thierno Mamadou TOUNKARA, Aïssatou Lamarana Bailo DIALLO, Joseph BALATH, Brigitte Singa WAKOU, Thierno Souleymane BAH, Mamadou Dioudé KANTE, Mariama Sadio DIALLO, Mohamed CISSE

**Affiliations:** Fondation Espoir de Guinée, CHU de DONKA, Conakry, Guinée

## Introduction

L’annonce du statut sérologique d’un enfant constitue une situation particulière qui ne peut obéir aux recommandations générales pour le *counseling* défini pour des adultes. L’objectif était de déterminer le motif de partage du statut sérologique aux enfants.

## Matériel et méthodes

Nous avons mené une étude transversale descriptive au CTA de Donka sur une période de 36 mois allant du 1^er^ janvier 2020 au 31 décembre 2022. Nous avons inclus tous les enfants et adolescents infectés par le VIH sous traitement antirétroviral (ARV) et avons recherché le motif de leur prise de médicaments de façon quotidienne.

## Résultats

Sur un total de 367 clients, 44 ont été recensés soit une prévalence de 11,9 %. L’âge moyen de nos clients était de 14 ans avec des extrêmes de 11 et 18 ans. Une prédominance féminine a été retrouvée (56,8 %). Le partage du statut sérologique VIH avec les clients était motivé par l’incompréhension du tuteur après le décès des parents dans 36,4 % des cas, le début de la sexualité active dans 13,6 % des cas, la négligence du traitement ARV dans 18,2 % des cas, le refus catégorique de prendre le traitement ARV dans 22,7 % des cas, et enfin les enfants ignorés par la famille après décès des deux parents dans 9,1 % des cas. Au total, tous les clients avaient adhéré et respecté les rendezvous prescrit pour la poursuite de la prise en charge.

## Conclusion

Notre étude retrouve une parfaite adhésion des clients après le partage du statut. Des stratégies d’intervention adaptées au contexte pédiatrique doivent être mise en place afin d’améliorer le devenir des enfants.

# Research article: N° 25 Les atteintes pulmonaires chez les personnes vivant avec le VIH (PVVIH) au Centre hospitalier universitaire (CHU) de Sidi Bel Abbés

**Published:** 2024-06-13

**Authors:** Samira BESTAOUI, Warda BOUCETTA, Nardjiss Feryel TABET-DERRAZ

**Affiliations:** CHU de Mostaganem, Algérie

## Introduction

Les atteintes pulmonaires chez le PVVIH ont un spectre très étendu et très varié. Ainsi, les infections opportunistes et les néoplasies à expression pulmonaire restent très préoccupantes chez cette population de nos jours. Leur diagnostic étiologique demeure lent et difficile et leur pronostic sévère.

## Objectif

Établir l’incidence de ces atteintes chez les PVVIH et un listing des différentes atteintes.

## Matériel et méthodes

Étude rétrospective sur dossier de PVVIH hospitalisés durant la période de janvier 2020 à décembre 2022, recensant les malades atteints de pneumopathies avec ou sans preuves étiologiques.

## Résultats

Sur 580 nouveaux PVVIH diagnostiqués, 120 avaient une atteinte pulmonaire soit 20,68 %. La tranche d’âge la plus touchée était celle des 31 et 55ans (46,7 %), avec une moyenne de 40,4 ans. On observait une légère prédominance masculine de 53 % avec un tabagisme actif dans presque la totalité des cas. Une atteinte pulmonaire était révélatrice de la séropositivité dans 60 % des cas. La pneumocystose s’observait dans 40 % des cas, une pneumopathie à pyogènes dans 25 % des cas, une tuberculose pulmonaire dans 30 % des cas, une néoplasie dans 5 % des cas et une infection à mycobactérie atypique dans 2 % des cas. La restauration immune sous forme d’une atteinte pulmonaire représentait 2 % des cas. L’atteinte des enfants représentait 3 % des malades avec une prédominance de la pneumocystose. La mortalité atteignait 10 %.

## Conclusion

L’atteinte pulmonaire chez le PVVIH reste réputée par sa fréquence, sa gravité et sa diversité. Souvent révélatrice de la maladie, elle doit être dépistée car elle peut rester insidieuse et être à l’origine de graves tableaux de restauration immune.

# Research article: N° 26 Comparaison de deux kits diagnostiques pour le dépistage sérologique de l'infection au virus de l'immunodéficience humaine (VIH)

**Published:** 2024-06-13

**Authors:** Amel CHTOUROU, Saba GARGOURI, Mouna MÂALOUL, Lamia FEKI-BERRAJAH, Héla KARRAYHAKIM

**Affiliations:** Unité de Virologie, Centre hospitalier universiataire (CHU) Habib Bourguiba, Sfax, Tunisie

## Introduction

Les kits ELISA de 4^e^ génération utilisés pour le dépistage sérologique de l’infection au VIH diffèrent en termes de performances analytiques.

## Objectif

Comparer l’apport de deux kits ELISA combinés de marque « Abia » et « Wantai » dans le diagnostic de l’infection au VIH.

## Matériel et méthodes

Étude rétrospective menée à l’Unité de virologie du Laboratoire de microbiologie du CHU Habib Bourguiba de Sfax, portant sur 88 sérums reçus durant 2020-2022 et testés à la fois par les kits Abia et Wantai. Les prélèvements positifs par l’un des kits ou les deux à la fois ont été confirmés soit par RT-PCR en temps réel à la recherche de l’ARN viral, soit par Western blot.

## Résultats

Parmi 88 prélèvements testés, 20 (22,7 %) étaient positifs par le kit Wantai *versus* 48 (54,4 %) par le kit Abia. Le taux de concordance globale entre les deux kits était de 68,2 %. La confirmation par RT-PCR et/ou Western blot a été réalisée pour 29 échantillons, dont 17 positifs par les deux kits et 12 positifs uniquement par le kit Abia. L’infection au VIH a pu être confirmée dans les 17 échantillons positifs par les deux kits et dans aucun cas parmi les 12 positifs uniquement par Abia. Les taux de concordance entre la sérologie par ELISA utilisant les kits Wantai et Abia et la technique de confirmation étaient respectivement de 100 % et 58,6 %.

## Conclusion

Cette étude démontre que le kit Wantai a une spécificité meilleure dans le dépistage de l’infection au VIH.

# Research article: N° 30 Prophylaxies post-exposition contre la rage chez les voyageurs : à propos de 2 916 patients vus dans un centre antirabique (CAR) à Paris en 2018-2022

**Published:** 2024-06-13

**Authors:** Patrick HOCHEDEZ, Kaoutar JIDAR, Fabien TAIEB, Oula ITANI, Ghania BENABDELMOUMEN, Perrine PARIZE, Hervé BOURHY, Paul-Henri CONSIGNY, Philippe POUJOL

**Affiliations:** Centre médical de l’Institut Pasteur, 211 rue de Vaugirard, 75015 PARIS, France

## Introduction

La rage cause environ 60 000 décès par an, principalement en Asie et en Afrique et les chiens sont responsables de 99 % des transmissions. Nous avons cherché à mieux connaître les caractéristiques des personnes exposées afin de renforcer les messages de prévention.

## Matériels et méthodes

Analyse rétrospective réalisée sur des patients pris en charge au CAR pendant la période 2018-2022, après exposition en zone d’endémie.

## Résultats

Au total, 2 916 patients ont consulté au CAR (femmes 50,5 %); l’âge médian était de 29,5 ans avec 462 (15,8 %) expositions avant l’âge de15 ans. Les trois animaux les plus souvent impliqués étaient le chien (47 %), le chat (28 %) et le singe (21 %). Une vaccination antirabique antérieure complète n’était rapportée que dans 9 % des expositions. Les régions d’exposition le plus fréquentes étaient l’Asie du Sud-Est (33,7 %) et l’Afrique du Nord (26 %). Comparée à l’Asie du Sud-Est, la proportion de femmes était plus faible parmi les personnes exposées en Afrique du Nord (45,2 % *vs* 52,4 %) et la proportion d’enfant de moins de 15 ans plus importante (27,5 % *vs* 10,5 %). L’animal le plus souvent impliqué différait selon la région : le singe en Asie du Sud-Est (48,4 %) et le chat en Afrique du Nord (54,7 %).

## Conclusion

Les voyageurs en région d’endémie pourraient bénéficier d’une information plus spécifique en fonction de la zone visitée, mais aussi de l’âge. Chez les voyageurs en Asie du Sud-Est ou en Afrique du Nord, les mesures de protection contre les risques liés aux animaux autres que les chiens (singe et chat, respectivement) devraient être renforcées.

# Research article: N° 31 Réponse post-vaccinale chez les sujets âgés après une prophylaxie post-exposition antirabique

**Published:** 2024-06-13

**Authors:** Oula ITANI, Patrick HOCHEDEZ, Ghania BENABDELMOUMEN, Kaoutar JIDAR, Fabien TAIEB, Perrine PARIZE, Paul Henri CONSIGNY, Philippe POUJOL

**Affiliations:** Institut Pasteur, Centre médical, Paris, France

## Introduction

La réponse vaccinale chez les sujets âgés est parfois modifiée, et de multiples facteurs (immunosénescence, comorbidités multiples etc.) peuvent entrainer une réponse immunologique insuffisante, comme en témoigne le décès récent de la rage d’un sujet âgé de 84 ans aux États-Unis malgré une prophylaxie post-exposition (PEP) bien conduite.

## Patients et méthodes

Nous avons évalué les patients de ≥ 70 ans ayant consulté dans un centre antirabique entre le 1^er^ mars 2016 et le 31 décembre 2022, ayant réalisé un contrôle sérologique (Platelia Rabies II kit, enzymelinked immunosorbent assay) au moins 15 jours après une PEP contre la rage.

## Résultats

179/5 339 patients ayant reçu une PEP avaient 70 ans et plus et un résultat sérologique était disponible pour 77/179. Le titre médian d’anticorps rabiques était de 3,26 EU/ml (interquartile, 1,9-4); 72 (93,5 %) avaient un titre considéré comme protecteur (>0,5 EU/ml) et cinq (6,5 %) insuffisant. Seul un patient parmi les cinq avec un titrage insuffisant était connu pour être immunodéprimé (traité par méthotrexate pour une spondylarthropathie ankylosante). La réponse immune post-vaccinale était satisfaisante après administration d’une dose supplémentaire chez quatre patients sur cinq (le dernier n’ayant pas poursuivi la prophylaxie en raison de la vitalité de l’animal).

## Conclusion

La réalisation d’une sérologie après une prophylaxie post-exposition contre la rage est souhaitable chez les sujets de plus de 70 ans, pour éviter de méconnaître une réponse immune insuffisante qui nécessiterait une ou plusieurs doses supplémentaires.

# Research article: N° 32 Évaluation de l'impact économique de la prophylaxie antirabique en post exposition sur la base des données de l'Institut Pasteur de Tunis (2018-2022)

**Published:** 2024-06-13

**Authors:** Sonia KECHAOU-CHERIF, Mariem HANDOUS, Mourad SELMI, Imen MANNAI, Anissa KHELIFI, Hanène DABBOUSSI, Ayoub RADDADI, Maroua BEN ALI, Anis RIAHI, Fathia SEDKI, Rahma JEMAI, Samia KADRI, Rim TAABOURI, Karim AOUN, Samy KHOUFI, Hechmi LOUZIR

**Affiliations:** Service des vaccinations internationales et antirabiques et Laboratoire de bioinformatiques, biomathématiques et biostatistiques, Institut Pasteur de Tunis, Tunis, Tunisie

## Introduction

La rage est une maladie négligée par excellence. Grâce à un programme national de lutte antirabique, la Tunisie a réussi à contrôler partiellement cette maladie sur le plan humain. Néanmoins les coûts engendrés par la prophylaxie antirabique en post exposition continuent à augmenter au fil des ans sans atteindre le zéro cas de rage humaine.

## Objectif

Évaluer ces coûts entre 2018 et 2022 et démontrer l’impact économique de cette maladie sur le pays à travers l’analyse des dossiers des personnes prises en charge à l’Institut Pasteur de Tunis durant cette période.

## Méthodes

Nous avons relevé les rapports d’activité et les rapports financiers du Service des vaccinations internationales et antirabiques entre 2018 et 2022.

## Résultats

Nous avons constaté que, durant cette période, le nombre de personnes prises en charge en post exposition a augmenté de 49,5 %. Le nombre de doses de vaccins utilisées est passé de 16 376 à 28 888, soit une hausse de 55,28 %. Le nombre de doses de sérum antirabique consommées est passé de 1 987 à 6 851. Les coûts liés à l’achat du vaccin et du sérum sont passés de 409 846 DT à 841 922 DT.

## Conclusion

L’impact économique de la lutte contre la rage liée seulement à la prophylaxie antirabique en post exposition est élevé et en hausse, sans compter les dépenses supplémentaires liée à la prise en charge des cas humains. Il faut, ainsi, réduire l’exposition des humains en repensant et renforçant les mesures de lutte et de contrôle de la rage animale.

# Research article: N° 34 La rage en Égypte en 2022 : situation et perspectives. Une analyse à travers l'approche « One Health »

**Published:** 2024-06-13

**Authors:** Odile CLAUSSE, Patrick THONNEAU

**Affiliations:** Centre médical des armées (CMA), 1^re^ antenne médicale de Paris, 60, boulevard du général Martial Valin, 75509 Paris Cedex 15 France

## Introduction

Dans le monde, la rage représente 60 000 décès annuels, essentiellement en Asie et en Afrique (95 %). L’Égypte, pays charnière entre ces deux continents n’est pas épargnée. Dans le cadre de l’objectif de développement durable (ODD) 3.3 visant notamment l’élimination des maladies tropicales négligées, la communauté internationale a décidé d’adopter l’approche « One Health » pour atteindre son objectif « zéro décès humain lié à la rage transmise par les chiens d’ici 2030 ». La stratégie mondiale coordonnée par l’Organisation mondiale de la santé, l’Organisation des Nations unies pour l’alimentation et l’agriculture (FAO) et l’Organisation mondiale de la santé animale, s’appuie sur trois axes : la prévention des morsures de chien, la prophylaxie postexposition et la vaccination animale. Cette stratégie est-elle effective en Égypte ?

## Méthode

Une recherche par mots-clés sur Pubmed et Google en 2022 a permis de collecter plusieurs sources qui ont fait l’objet d’une synthèse réalisée à l’Université Senghor d’Alexandrie.

## Résultats

L’Égypte fait face à une population urbaine et rurale de chiens errants d’environ 15 millions en 2019. La couverture vaccinale des chiens est estimée à 23 % pour une cible minimale de 70 %. En 2017, le ministère de l’agriculture égyptien annonçait 400 000 victimes de morsures de chiens. Plus de la moitié d’entre elles reçoivent une vaccination curative. Enfin plusieurs sources s’accordent sur un nombre annuel de décès humains de la rage de quelques dizaines.

## Conclusion

Si l’Égypte dispose d’atouts indéniables comme la gratuité de la prophylaxie post-exposition, des études sur l’écologie canine et auprès des populations victimes de morsures permettraient de mieux cibler les programmes de lutte contre la rage pour atteindre « zéro décès en 2030 ».

# Research article: N° 36 Accès au test Covid-19 et positivité chez des enfants immigrés et natifs âgés de 2 à 6 ans dans la région de Lisbonne entre 2020-2022

**Published:** 2024-06-13

**Authors:** Maria Rosàrio OLIVEIRA MARTINS, Iolanda ALVES, Sofia Gonçalves SEABRA

**Affiliations:** Global Health and Tropical Medicine, Instituto de Higiene e Medicina Tropical, Universidade Nova de Lisboa, Lisboa, Portugal

## Introduction

Les enfants immigrés ou fils d'immigrés, par rapport aux enfants natifs, ont tendance à montrer une susceptibilité, une exposition et une vulnérabilité accrues au Covid-19. L’objectif de cette étude a été d’analyser l’association entre le statut de migrant, l’accès au test et la positivité du Covid-19 chez les enfants résidant dans la région de Lisbonne, Portugal.

## Méthodes

On a retenu 989 enfants nés entre 2015 et 2020 (48,5 % de natifs; 51,5 % d’immigrés). Les données ont été collectées à partir de questionnaires et sources administratives, notamment le système de surveillance national Covid-19 entre mars 2020 et juin 2022. On a calculé les rapports de prévalence (PR) et les risques relatifs (RR) ainsi que les intervalles de confiance à 95 % par l’estimation des modèles de Poisson et Poisson robustes.

## Résultats

La proportion d’enfants d’immigrés ayant effectué au moins un test Covid-19 était inférieure (75 % contre 78 %), comme le nombre total de tests, inférieur de 16 % comparé à celui des enfants natifs (RR = 0,839, IC à 95 % : 0,770-0,915). Cependant, la proportion d’enfants immigrés testés positifs au moins une fois est inférieure (21 % contre 49 %), avec une probabilité d’être testé positif au moins une fois inférieure de 53 % (PR = 0,473, IC à 95 % : 0,381-0,587) et un nombre total de tests positifs inférieur de 52 % (RR = 0,478, IC à 95 % : 0,375-0,608) comparés aux natifs.

## Conclusion

Ce résultat demande des investigations plus approfondies sur le statut immunologique des populations et facteurs associés.

# Research article: N° 37 Oxygénothérapie à haut débit nasal lors de la pneumopathie à Covid-19, à l'Unité de soins intensifs de Sfax

**Published:** 2024-06-13

**Authors:** Sana KHARRAT, Sabrine BRADAI, Karama BOUCHAALA, Emna ZOUARI, Najeh BACCOUCHE, Chokri BEN HAMIDA, Mabrouk BAHLOUL, Mounir BOUAZIZ

**Affiliations:** Service de réanimation médicale, Centre hospitalier universitaire (CHU) Habib Bourguiba, Sfax, Tunisie

## Introduction

La pandémie Covid-19 est responsable d’insuffisance respiratoire aiguë hypoxémique. Afin de diminuer le recours à la ventilation invasive, l’oxygénothérapie haut débit par canule nasale (OHD) a été largement recommandée.

## Objectif

Le but de cette étude est de décrire le profil épidémiologique, clinique, paraclinique et évolutif des patients ayant bénéficié de l’OHD.

## Méthode

C’est une étude rétrospective, descriptive et analytique incluant tous les patients hospitalisés pour pneumopathie à Covid-19 et ayant l’OHD comme moyen d’oxygénothérapie sur une période de neuf mois.

## Résultat

Durant la période de l’étude, 530 patients ont été admis, dont 157 ont été mis sous OHD (29,8 %). L’âge moyen était 59,5 ± 14,5 ans avec un sex-ratio de 1,37. Soixante-huit patients (43,3 %) avaient une hypertension, 50 patients (31,8 %) étaient diabétiques et 12 patients (7,6 %) avaient une insuffisance respiratoire chronique. Le score SAPS II moyen était de 29,5 ± 10. Un syndrome de détresse respiratoire aiguë (SDRA) sévère était observé chez 140 patients (89,2 %). Quatre-vingt-huit patients (56 %) avaient une atteinte parenchymateuse supérieure à 50 %. Chez tous les patients, le débit était à 60 l/min et la FiO2 moyenne était de 88 % ± 12. Le pH moyen était à 7,42 ± 0,06, la PaCO2 en moyenne était à 36,13 mm Hg ± 7,1, la PaO2 moyenne sous OHD était de 68,10 mm Hg ± 29. Le rapport PaO2/FiO2 moyen était à 83 mm Hg ± 35. Soixante-dix patients (70,7 %) ont eu recours à la ventilation mécanique invasive. Cent quatre patients (66,2 %) sont décédés. En analyse univariée, les facteurs associés à la mortalité ont été l’âge avancé (0,032) et le recours à la ventilation mécanique (p = 0,000) et un rapport PaO2/FiO2 <70 à l’admission (p = 0,003).

## Conclusion

Dans notre série les patients qui ont bénéficié du haut débit nasal étaient d’emblée grave et très hypoxémique expliquant le pronostic péjoratif de nos patients.

# Research article: N° 39 Covid-19 : connaissances, attitudes et pratiques de prévention des immigrés angolais au Portugal

**Published:** 2024-06-13

**Authors:** Licinia ARAGÄO, Ricardo PEREIRA IGREJA, Rosa TEODÓSIO

**Affiliations:** Faculdade de Medicina, Universidade Federal do Rio de Janeiro, Rio de Janeiro, Brésil

## Introduction

La pandémie de Covid-19 a été responsable de millions de cas dans le monde. Certaines communautés de migrants ont été identifiées comme étant plus vulnérables à l’infection, en raison, entre autres facteurs, d’un manque d’informations sur les mesures de prévention. Les objectifs de ce travail sont de décrire les connaissances et les pratiques de prévention du Covid-19 chez les immigrants angolais au Portugal.

## Méthodes

Questionnaire *online* utilisant la plateforme Survey Monkey^®^. Population : immigrants angolais âgés ≥18 ans, résidant au Portugal ≥6 mois. Le questionnaire a été administré de décembre 2021 à février 2022, en personne et *via* les réseaux sociaux.

## Résultats

Sept cent vingt personnes ont participé, dont 64,7 % de femmes.

### Connaissances

*Transmission* : éternuements/toux (54,4 %), surfaces contaminées (46,5 %), aliments contaminés (3,9 %); *Prévention* : éviter les rassemblements (54,6 %), porter un masque (51,7 %), se désinfecter les mains (3,8 %), manger des aliments bien cuisinés (2,6 %). *Signes/symptômes* : toux (53,1 %), fièvre (50 %), diarrhée (8,2 %). Dans un sousgroupe de 412 personnes interrogées, 10 % ne savaient pas si la vaccination prévenait ou non la maladie.

### Attitudes

seuls 5,3 % ne considéraient pas la vaccination comme importante.

### Pratiques de prévention

masque dans les transports en commun (54,3 %), éviter les rassemblements (50,3 %), lavage des mains (47,9 %), masque dans les établissements commerciaux (49,2 %).

## Conclusions

Les participants à l’étude montrent de sérieuses lacunes dans leurs connaissances sur la transmission et la prévention du Covid-19. Seule la moitié utilisait des formes de prévention adéquates. Cependant, la majorité a montré une attitude favorable à la vaccination.

# Research article: N° 40 Point d'avancement du développement clinique d'un candidat vaccin vivant atténué contre le chikungunya, administré en injection unique

**Published:** 2024-06-13

**Authors:** Martina SCHNEIDER, Marivic NARCISO-ABRAHAM, Romana HOCHREITER, Karin KOSULIN, Robert MADER, Vera BUERGER, Olivier ZOIHSL, Katrin DUBISCHAR, Nina WRESSNIGG, Susanne EDERLINGELBACH, Juan-Carlos JARAMILLO

**Affiliations:** Valneva Îlot Saint-Joseph Bureaux Convergence, 12 ter Quai Perrache Bâtiment A, 69002 Lyon, France

VLA1553 est un candidat vaccin vivant atténué pour l’immunisation active contre la maladie causée par le virus du chikungunya (CHIKV). VLA1553 est basé sur un clone de la souche de La Réunion (CHIKV LR2006-OPY1) et est atténué par la suppression d’une partie du génome du virus. Les principaux résultats cliniques du candidat-vaccin VLA1553 sont présentés dans ce résumé. À la suite d’un plan d’études pré-clinique, VLA1553 a été l’objet d’études cliniques. Un essai de phase 1 randomisé et en aveugle, a révélé des titres d’anticorps neutralisants spécifiques du CHIKV élevés et maintenus dans tous les groupes de doses, ce qui a conduit à la sélection de la dose moyenne pour la suite du développement. Un essai pivot de phase 3 impliquant 4 115 adultes a atteint son objectif principal, le vaccin candidat VLA1553 induisant une séro-réponse (µPRNT50 ≥150) chez 98,9 % des participants 28 jours après une seule vaccination, qui s’est maintenue pendant au moins un an. Le taux élevé de séroréponses a été observé chez les participants âgés de 18 à 64 ans et chez les adultes âgés de 65 ans et plus. VLA1553 a été généralement bien toléré avec un profil d’innocuité acceptable dans les deux groupes d’âge.

# Research article: N° 41 Données de tolérance préliminaires d'un candidat vaccin vivant atténué contre le virus du chikungunya chez les adolescents au Brésil : étude pivot^1^1Cette étude est menée par l’Instituto Butantan en tant que sponsor local et Valneva en tant que responsable du développement, financée par le CEPI (Coalition pour les innovations en matière de préparations aux épidémies), avec le soutien du programme de l’Union européenne EU Horizon 2020.

**Published:** 2024-06-13

**Authors:** Vera BÜRGER, Sandra HADL, Martina SCHNEIDER, Michaela SCHADEN, Jutta SEEBACHER, Romana HOCHREITER, Annegret BITZER, Karin KOSULIN, Robert MADER, Olivier ZOIHSL, Andrea PFEIFFER, Ana Paula LOCH, Eolo MORANDI, Katrin DUBISCHAR, Nina WRESSNIGG, Susanne EDER-LINGELBACH, Juan-Carlos JARAMILLO

**Affiliations:** 1 Valneva Austria GmbH, Campus-Vienna-Biocenter 3, 1030 Wien, Autriche; 2 Instituto Butantan, Av. Vital Brasil, 1500 - Butantä, Säo Paulo - SP, 05585-000, Brésil

VLA 1553 est un candidat vaccin vivant atténué contre le virus du chikungunya (CHIKV).

L’objectif de cet essai pivot randomisé, en double aveugle, contrôlé versus placebo (NCT04650399)* est d’évaluer l’immunogénicité et la tolérance de VLA1553 chez les adolescents de 12 à < 18 ans, en prenant en compte leur statut sérologique initial vis-àvis du chikungunya. Parmi les 754 participants, ~20 % avaient des anticorps préexistants contre le CHIKV.

Le recrutement a été terminé en février 2023 et, en juin 2023, les visites du 6^e^ mois ont été effectuées pour 61 % des participants. 49 % des participants étaient âgés de 12 à 14 ans et 51 % de 15 à 17 ans. Un Data Safety Monitoring Board (DSMB) indépendant a examiné régulièrement les données de tolérance et n’a soulevé aucun signal à ce jour. Dans cette étude, aucun effet indésirable grave lié au vaccin n’a été signalé à ce jour, quel que soit le statut sérologique de départ.

Une analyse plus approfondie des données, dont le suivi à six mois et l’immunogénicité jusqu’à un an, devrait permettre d’étayer les demandes d’homologation en vue d’une indication de VLA1553 chez les adolescents. Ces données fourniront en outre des informations importantes sur le profil de tolérance et d’immunogénicité du vaccin chez les personnes précédemment exposées au virus, en vue de son utilisation dans les régions endémiques pour le chikungunya.

# Research article: N° 42 Exploration virologique de cas suspects d'infection neurologique à virus West Nile survenus durant l'automne 2023 en Tunisie

**Published:** 2024-06-13

**Authors:** Mouna MAALOUL, Saba GARGOURI, Amel CHTOUROU, Yasmine CHAARI, Souha BOUGATF, Fares WASFI, Henda TRIKI, Lamia FKI, Hela KARRAY

**Affiliations:** 1 Unité de virologie, Laboratoire de microbiologie, Centre hospitalier universitaire (CHU) Habib Bourguiba Sfax, Tunisie; 2 Observatoire national des maladies nouvelles et émergentes, Tunisie; 3 Laboratoire de Virologie, Institut Pasteur de Tunis, Tunisie, Unité de virologie, Laboratoire de microbiologie, CHU Habib Bourguiba Sfax, Tunisie

## Introduction

Des atteintes neurologiques groupées ont été signalées en Tunisie durant l’automne 2023, faisant suspecter une infection par le virus West Nile (WNV), dont l’activité a augmenté ces dernières années dans le monde.

## Objectif

Explorer les tableaux neurologiques évoquant l’infection par le WNV par des outils diagnostiques sérologiques et moléculaires.

## Matériel et méthodes

Il s’agit d’une étude menée au laboratoire de microbiologie, unité de virologie, du CHU Habib Bourguiba de Sfax, portant sur huit patients hospitalisés pour un tableau neurologique faisant suspecter une infection par le WNV, durant la période septembre-novembre 2023. La sérologie et la recherche de l’ARN viral ont été réalisées respectivement par test ELISA et RT-PCR en temps réel.

## Résultats

Parmi les patients étudiés, cinq ont été originaires du gouvernorat de Tozeur. Un profil sérologique montrant des IgM positives, en l’absence des IgG, a été retrouvé sur le premier sérum de tous ces patients (8/8). Quant au liquide céphalorachidien (LCR), des IgM positives ont été détectées dans cinq cas parmi les sept LCR collectés. Des sérums de contrôle ont été reçus pour sept patients, dont cinq ont montré une séroconversion des IgG, avec un délai variant par rapport à la date du premier sérum, de 2 à 21 jours. Parmi ces cinq derniers patients, deux cas ont pu être confirmés par la détection de l’ARN viral dans les urines : un cas était originaire de Tozeur et l’autre de Sfax.

## Conclusion

Cette étude illustre bien l’apport du diagnostic sérologique et moléculaire pour le diagnostic de l’infection à WNV, tout en soulignant le caractère inhabituel du délai prolongé de la séroconversion et le faible nombre de PCR positives.

# Research article: N° 44 Particularités épidémiologiques de la rougeole à Brazzaville et facteurs associés à propos de 65 cas au Centre hospitalier universitaire (CHU) de Brazzaville

**Published:** 2024-06-13

**Authors:** Tatia ADOUA DOUKAGA, Bienvenu Rolland OSSIBI IBARA, Martin EKAT, Ella ANGONGA PABOTA, Princesse BENDETT LEBAHO, Prisca DINGA, Frédy DIMI, Dom KANDZA, Aurore MBIKA CARDORELLE

**Affiliations:** 1 Faculté des sciences de la santé, Université Marien Ngouabi Brazzaville, République du Congo; 2 Service des maladies infectieuses, CHU de Brazzaville, République du Congo; 3 Service de pédiatrie grands enfants, CHU de Brazzaville, République du Congo

## Objectif

Décrire les particularités épidémiologiques de la rougeole après des campagnes de vaccination et identifier les facteurs associés à la maladie.

## Patients et méthode

Étude prospective à visée descriptive et analytique des cas de rougeole chez les enfants âgés d’au moins un an, admis dans le Service des maladies infectieuses du CHU de Brazzaville durant la période allant du 1^er^ mars au 15 décembre 2023.

## Résultats

Au total 65 enfants d’âge moyen de 31,6 ± 33,5 [3-168] mois, de sexe masculin (n = 39; 60 %), non scolarisés (n = 53; 81,6 %), résidant en ville (n = 58; 89,2 %), non vaccinés (n = 45; 69,2 %), ayant un poids médian à la naissance de 3 kg. L’âge moyen de diversification était de 4,4 mois [1-6] mois. Les enfants occupaient le 2^e^ rang dans la fratrie dans 19 cas (29,2 %). On notait une notion de contage (n = 40; 63,1 %). L’âge moyen des mères était de 29,6 [18-44] ans. La tranche d’âge la plus représentée était celle comprise entre 20 et 29 ans (n = 28; 43,1 %), de profession ménagère (n = 21; 32,3 %). Elles avaient un niveau de scolarisation au primaire (n = 21; 32,3 %), vivant en union libre (60; 92,3 %). On notait un bas niveau socioéconomique (n = 58; 89,2 %). Les enfants était au moins au nombre de trois dans la fratrie (n = 13; 20 %). L’éruption fébrile était le principal motif d’admission (n = 48; 73,8 %) et l’IMC moyen était de 14,2 [10,4-18,4] kg/m^2^. La radiographie retrouvait l’anomalie dans 17 cas (26,2 %). Le statut VIH n’était pas connu (n = 51; 78,5 %) et la goutte épaisse et la recherche d’hématozoaire (GERh) était positive (n = 22; 33,8 %). La durée moyenne d’hospitalisation était de 6,5[1-15] jours. L’évolution était défavorable dans deux cas (3,1 %). Le niveau d’instruction des parents avait un lien avec la survenu de décès (p = 0,001).

## Conclusion

La rougeole demeure d’actualité à Brazzaville malgré les campagnes de vaccination signifiées dans les médias. Le faible taux de couverture en lien avec soit le refus des parents à faire vacciner les enfants, soit le manque de volonté des vaccinateurs justifie en partie la résurgence des cas de rougeole.

# Research article: N° 45 Séroprévalence de la rubéole chez les femmes enceintes de la région de Rabat, Maroc

**Published:** 2024-06-13

**Authors:** Amal ZOUAKI, Hafsa LAMRANI ALAOUI, Ghizlane EL AMIN, Myriam SEFFAR, Hakima KABBAJ

**Affiliations:** Laboratoire central de virologie, Hôpital des spécialités, Centre hospitalier universitaire Ibn Sina de Rabat, Faculté de médecine et de pharmacie de Rabat, Maroc

## Introduction

La rubéole est une maladie bénigne mais qui peut être grave en cas de primo-infection chez la femme enceinte avant la 18^e^ semaine d’aménorrhée vu le risque de rubéole congénitale. L’objectif de cette étude est de déterminer la séroprévalence de la rubéole chez les femmes enceintes dans la région de Rabat au Maroc.

## Patients et méthodes

C’est une étude prospective menée au niveau du CHU Ibn Sina du Rabat pendant une durée de huit mois. Tous les prélèvements ont fait l’objet d’une analyse micro-particulaire par chimiluminescence sur l’ARCHITECT Abbott pour la détection et la quantification des IgG anti-rubéole. Un titre d’anticorps ≥ 10 UI/ml est considéré protecteur. Nous avons exclu les sérologies douteuses de l’étude.

## Résultats

Sur 502 femmes enceintes incluses dans l’étude, 408 avaient des IgG anti-rubéole positifs avec une séroprévalence globale de 85,9 %. La séroprévalence varie significativement avec l’âge : 89,8 % chez les patientes âgées de moins de 30 ans *versus* 81,0 % chez les patientes âgées de plus de 30 ans (p = 0,006). La médiane des titres des IgG anti-rubéole était de 34,52 [14,95-70,5] UI/ml et nous n’avons pas observé de différence statistiquement significative entre les deux populations (médiane de 35,5 [18,5-72] *vs* 31,4 [10,7-67,1] respectivement, p : 0,158).

## Conclusion

Grâce au programme de vaccination contre la rubéole, la primo-infection maternelle est en baisse. Cependant, nos résultats montrent que 14,1 % des femmes enceintes ne sont pas protégés contre la rubéole. Le dépistage et le renforcement de la vaccination restent essentiels afin de prévenir la survenue la rubéole congénitale.

# Research article: N° 46 Les auto-anticorps et infection au virus de l'hépatite C (VHC) : prévalence et pronostic

**Published:** 2024-06-13

**Authors:** Nabila KALLA, Ouanassa HAMOUDA, Lina NOUI, Amani MELIZI, Souhila AOUIDANE, Zohra MERZOUGUI

**Affiliations:** Faculté de médecine, Université Batna 2, Batna, Algérie

## Introduction

Il a été constaté que certains patients infectés par le VHC avaient des autoanticorps sériques à savoir les anticorps antinucléaires, anti-muscles lisses, anti-LKM1. L’objectif de notre travail est d’évaluer la prévalence de ces auto-anticorps et leur impact pronostic.

## Matériels et méthodes

Il s’agit d’une étude rétrospective menée au Service de médecine interne et des maladies infectieuses de notre établissement, colligeant les patients suivis pour hépatite chronique C entre 2003-2016. Les données épidémiologiques, immunologiques, virologiques et histologiques ont été recueillies. Une analyse univariée et multivariée par régression logistique a été faite.

## Résultats

Parmi les 370 patients qui ont été colligés, 48 ont présenté au moins un des auto-anticorps (12,9 %). Il s’agit de 39 (81,3 %) femmes et de 9 (18,7 %) hommes. L’âge moyen de ces patients est de 56,04 ans (30 - 71). Les anticorps anti-muscles lisses, les anti-nucléaires et les anticorps anti-LKM1 sont retrouvés respectivement chez 19, 20, 2 malades. Sur 291 patients, 19 patients ont présenté des anticorps anti-thyroperoxydases. Aucun patient n’a présenté une maladie auto-immune associée. L’analyse statistique a montré que la présence des auto-anticorps sériques n’est pas significativement associée aux activités des transaminases sériques (P à 0,448), à la charge virale (P à 0,141), au sexe (P à 0,105) ni à la présence d’une fibrose sévère (p à 0,089).

## Conclusion

Les auto-anticorps étaient constatés chez environ 13 % des patients infectés par le VHC. Aucune association significative n’a été objectivée entre la présence des auto-anticorps et la sévérité de l’atteinte hépatique chez eux.

# Research article: N° 47 Séroprévalence du VHE chez les donneurs de sang au Maroc

**Published:** 2024-06-13

**Authors:** Ghizlane EL AMIN, Myriam SEFFAR, Yasser BOUBEL, Jalila ZIRAR, Amal ZOUAKI, Hakima KABBAJ

**Affiliations:** Laboratoire central de virologie - Hôpital des spécialités, Centre hospitalo-universitaire Ibn Sina, Rabat, Maroc

## Introduction

Le virus de l’hépatite E (VHE) sévit en mode épidémique dans les pays en voie de développement. L’objectif de notre étude était de déterminer la séroprévalence de l’infection par le VHE chez les donneurs de sang au Maroc.

## Patients et méthodes

Étude prospective transversale s’étalant sur une période de six mois, où nous avons collecté 100 échantillons de sang total sur tube sec chez des donneurs de sang du centre régional de transfusion sanguine de Rabat. La détection des Ac anti-HEV IgG a été réalisée sur l’automate Mini VIDAS^®^, BioMérieux au niveau du laboratoire central de virologie.

## Résultats

Sur les 100 donneurs inclus dans l’étude, 53 % étaient des hommes et 47 % des femmes avec un sexe ratio H/F de 1,1.

La séroprévalence globale de l’hépatite virale E était de 8 % avec une augmentation liée à l’âge : 0 % [18-24], 1 % (25-34), 2 % (35-44), 5 % (45-65), p 0,03. La consommation de viandes exotiques (lapins, dromadaires.. .etc.) a été rapportée chez 63 des donneurs dont les 8 patients positifs (8 *versus* 0, p 0,024). Nous n’avons pas noté de différence significative concernant les autres paramètres à savoir le sexe, le niveau socio-économique et l’habitat d’origine.

## Conclusion

La séroprévalence du virus de l’hépatite E restait faible dans la population de donneurs marocains sur notre série. Son augmentation avec l’âge serait imputée à l’amélioration de l’hygiène de vie au Maroc, notamment dans les régions urbaines et/ou à la consommation de viande exotique soutenant un éventuel risque zoonotique sporadique.

# Research article: N° 48 Épidémiologie de la leucoencéphalopathie multifocale progressive. Expérience du service des maladies infectieuses de Marrakech

**Published:** 2024-06-13

**Authors:** Rania EL FARGANI

**Affiliations:** Service des maladies infectieuses, Centre hospitalier universitaire (CHU) Mohamed VI Marrakech, Maroc

## Introduction

La leuco-encéphalopathie multifocale progressive (LEMP) est une pathologie démyélinisante rare du sujet immunodéprimé, due à la réactivation du polyomavirus JC dans le système nerveux central.

## Matériels et méthodes

Étude menée au sein du Service des maladies infectieuses entre 2007 et 2023 en étudiant les caractéristiques cliniques, paracliniques et évolutives des LEMP.

## Résultats

Au total, 12 patients. L’âge moyen de nos patients était de 35 ans [22-48 ans] avec une prédominance masculine et un sexe ratio de 3,33. Le délai moyen entre le début des symptômes et la consultation était de 57 jours [17 jours- 150 jours]. Les différents symptômes étaient : des troubles du comportement (31 %), des convulsions (3 %), l’hémiparésie (17 %), l’aphasie (10 %), des troubles d’équilibre (3 %), des troubles sphinctériens (7 %), une dysarthrie (3 %), des hallucinations (10 %), des céphalées (3 %) et une paraparésie dans 3 % des cas avec une paresthésie dans 7 % des cas. La LEMP était confirmée par PCR chez cinq patients (42 %) et le diagnostic était retenu devant les lésions d’IRM chez trois patients (25 %). La LEMP a été retenue devant un faisceau d’arguments chez quatre patients (33 %). L’IRM réalisée chez la plupart de nos patients a révélé un hypersignal T2 en plage intéressant la substance blanche. La valeur moyenne des CD4 était 42. L’évolution était favorable chez huit patients (67 %); quatre patients (42 %) ont cependant gardé des séquelles à type de dysarthrie.

## Conclusion

La leuco-encéphalopathie multifocale progressive (LEMP) est une maladie rare et grave, pour laquelle la seule stratégie actuellement validée est la reconstitution immunitaire.

# Research article: N° 50 Prévalence des infections au virus respiratoire syncitial (VRS) dans les infections respiratoires aiguës sévères (IRAS) au Centre hospitalier universitaire (CHU) Ibn Sina, Rabat-Maroc en 2023

**Published:** 2024-06-13

**Authors:** Nora TOUYAR, Amal ZOUAKI, Ghizlane EL AMINE, Hakima KABBAJ

**Affiliations:** Laboratoire central de virologie, Centre hospitalouniversitaire Ibn Sina de Rabat, Maroc; Faculté de médecine et de pharmacie de Rabat, Université Mohammed V, Rabat, Maroc

## Introduction

Le virus respiratoire syncitial (VRS) est un agent majeur d’infections respiratoires essentiellement chez les nourrissons et qui culmine en hiver. L’ère de la pandémie post Covid-19 a été marquée par une modification de l’épidémiologie des IRAS dont les bronchiolites à VRS.

## Objectif

Préciser l’épidémiologie de l’infection à VRS chez les patients hospitalisés pour IRAS au CHU Ibn Sina de Rabat pour l’année 2023.

## Matériels et méthodes

Il s’agit d’une étude rétrospective portant sur 1 032 prélèvements respiratoires provenant des patients hospitalisés au CHU Ibn Sina de Rabat pour IRAS durant l’année 2023, chez lesquels un test PCR multiplex respiratoire par le kit *FilmArray BioFire^®^ Respiratory Panel 2.1 Plus* a été réalisé.

## Résultats

Sur les 1 032 échantillons, 98 étaient positifs pour le VRS avec une prévalence globale de 9,5 %. La médiane d’âge des positifs était de cinq [1-33] mois et un sexratio M/F de 1,5. La prévalence de l’infection à VRS variait significativement avec l’âge : 20,1 % chez les nourrissons de moins de 6 mois, 13,3 % entre 6 mois à 2 ans, 10,9 % entre 2 à 5 ans et 3,8 % chez les plus de 16 ans tandis qu’aucun cas positif n’a été détecté chez les enfants âgés de 5 à 16 ans (p<0,001). La prévalence variait significativement selon les saisons : 15,6 % en hiver, 3,3 % en printemps, 3,8 % en automne et 5,7 % en été (p<0,001).

## Conclusion

Au cours de l’année 2023, l’infection grave à VRS a culminé en hiver en touchant essentiellement la population des nourrissons.

# Research article: N° 51 Prévention et contrôle de l'épidémie de dengue dans le district sanitaire de Cocody-Bingerville (Abidjan, Côte d’Ivoire)

**Published:** 2024-06-13

**Authors:** Diakaridia FOFANA, Atioumounan Blaise KONE, Jean Michel Vianney BEUGRE, Lambert Kouassi KONAN, Adama DIALLO, Salifou KONE, Kassoum OUATTARA, Daouda COULIBALY, Issaka TIEMBRE, Bi Vroh Joseph BENIE

**Affiliations:** Service de lutte antivectorielle, Institut national d’hygiène publique, Abidjan, BP V 14 Abidjan, Côte d’Ivoire

## Introduction

La Côte d’Ivoire est confrontée depuis 2008 à des épidémies de dengue. En avril 2017, une grande épidémie a été déclarée dans le district sanitaire de Cocody-Bingerville, au début de la grande saison des pluies. En l’absence de traitement spécifique et de vaccin fiable en Côte d’Ivoire, la seule mesure de riposte envisageable restait la lutte contre les moustiques vecteurs. C’est dans ce contexte qu’un plan de gestion intégrée de la lutte antivectorielle a été mis en œuvre afin d’interrompre la chaîne de transmission du virus épidémique.

## Méthodes

La riposte contre l’épidémie a consisté à rechercher activement les cas humains de dengue, à sensibiliser les populations, à gérer les gîtes larvaires et à réaliser la pulvérisation spatiale de produits insecticides.

## Résultats

Au total 315 cas de dengue 3 (DENV-3) ont été notifiés par le système de surveillance épidémiologique. Sur 22 468 ménages recensés, 19 385 ont été visités (soit un taux d’accessibilité de 86,3 %) et des prospectus de sensibilisation sur la dengue ont été distribués. Sur 220 972 gîtes larvaires répertoriés, 216 217 (97,8 %) ont été traités ou éliminés. La proportion de gîtes larvaires non traités et non éliminés a été estimée à 2,2 %. La pulvérisation spatiale a permis de réduire considérablement la densité des populations *dAedes.*

## Conclusion

Quatorze semaines d’activités de lutte contre les moustiques ont permis de mettre fin à l’épidémie de dengue. Aucun nouveau cas n’a été signalé à Abidjan sur le reste de l’année 2017.

# Research article: N° 53 Le paludisme d'importation : expérience d'un hôpital algérien

**Published:** 2024-06-13

**Authors:** Benyoucef CHACHOU, Djaffar BACHA, Maamer BOUHABEL, Abdelmalek MATARI, Faicel HAOUES, Hakim LAFSIHEN, Ilyes AKHAMOUK, Aziza HANDEL, Azzedine BENOHARETS

**Affiliations:** Service des maladies infectieuses, Hôpital universitaire de Blida, Blida, Algérie

## Introduction

La wilaya de Tamanrasset, au sud de l’Algérie, reste le premier foyer de paludisme d’importation, avec une menace permanente d’apparition de foyer autochtone. Notre objectif est de décrire le profil épidémio-clinique, thérapeutique et évolutif d’un nombre très élevé de cas de paludisme d’importation.

## Patients et méthodes

Étude rétrospective et descriptive, réalisée à l’hôpital de Tamanrasset, Algérie, durant une période de douze mois (Janvier-Décembre 2012). Le diagnostic est établi selon les données épidemio-cliniques et les résultats parasitologiques du laboratoire.

## Résultats

Au total 751 cas sont répertoriés, issus des villes frontalières des pays voisins. Le sex-ratio est de 3,34. Après une accalmie au premier semestre, on note une élévation rapide du nombre des cas dès le mois de juillet avec un pic de 350 cas pour le seul mois de septembre; 625 (83,2 %) patients sont de nationalité algérienne; 736 cas (98 %) sont dus à *Plasmodium falciparum.* Seulement 79 cas (10,5 %) sont hospitalisés pour une forme grave. Quatre décès sont enregistrés. Aucun moyen de prophylaxie n’est retrouvé.

## Conclusion

Le dépistage rapide des cas de paludisme d’importation et l’application rigoureuse du plan d’action approprié à la région ont permis une prise en charge efficace des patients et une réduction du nombre de décès. En l’absence de vaccination antipaludique efficace, la prévention repose sur la protection mécanique individuelle et la chimioprophylaxie correcte.

# Research article: N° 54 Paludisme d'importation à l'Hôpital militaire Mohammed V de Rabat, Maroc

**Published:** 2024-06-13

**Authors:** Kholoud MAAFI, Maryem IKEN, Laila BOUMHIL, Badre Eddine LMIMOUNI, Hafida NAOUI

**Affiliations:** Service de parasitologie et de mycologie médicale, Hôpital militaire d’instruction Mohammed V, Rabat, Maroc; Faculté de médecine et de pharmacie, Université Mohamed V, Rabat, Maroc

## Introduction

Le paludisme engendre annuellement des milliers de décès dans le monde, principalement en Afrique. Au Maroc, le dernier cas de paludisme autochtone remonte à 2004. Néanmoins, le pays continue de recenser un nombre de plus en plus important de cas importés.

## Patients et méthodes

Il s’agit d’une étude rétrospective descriptive sur l’année 2023, menée au sein du Laboratoire de parasitologie-mycologie de l’Hôpital militaire Mohammed V de Rabat, Maroc. Tous les patients pour lesquels une demande de frottis mince et de goutte épaisse a été formulée ont été inclus.

## Résultats

Durant l’année 2023, 242 patients ont été retenus, dont 41 cas positifs (une prévalence de 16,94 %) : 35 sont des Marocains et les 6 autres sont des migrants (d’origine congolaise, burundaise, camerounaise et guinéenne). L’analyse de la répartition mensuelle a révélé une augmentation significative pendant l’été.

L’âge moyen des patients impaludés était de 31,5 ans [23 et 71 ans]. Tous les cas présentaient des antécédents de séjour récent en zone d’endémie. Vingt-deux patients (55,66 %) avaient séjourné au Congo, suivis de la République Centrafricaine avec 13 cas (31,70 %). *P. ovale* était l›espèce la plus fréquemment retrouvée (19 cas), suivie de *P. falciparum* (17 cas) et de *P. malariae* (2 cas). Trois patients présentaient une co-infection due au *P. falciparum* et *P. ovale.* La parasitémie variait entre 0,01 % et 9,1 %.

## Conclusion

Tous ces patients ont été mis sous Arthemeter et Luméfantrine plus une dose de Primaquine. Le diagnostic parasitologique réalisé le J1, J3 et J28, a révélé une bonne efficacité thérapeutique avec un seul cas d’échec thérapeutique.

# Research article: N° 55 Immigrés guinéens au Portugal : notion de risque et prévention du paludisme lors d'un voyage vers leur pays d'origine

**Published:** 2024-06-13

**Authors:** Jaime TCHUDA, Ricardo PEREIRA IGREJA, Rosa TEODÓSIO

**Affiliations:** Faculdade de medicina, Universidade federal do Rio de Janeiro, Rio de Janeiro, Brésil

## Introduction

En Europe, la plupart des cas de paludisme sont survenus chez des immigrants/voyageurs qui rendaient visite à leur famille/amis. Parmi les étrangers au Portugal, les Guinéens constituent la dixième nationalité la plus représentée (24 000 individus).

## Objectif

Caractériser les connaissances des immigrés guinéens au Portugal sur le risque et la prévention du paludisme.

## Méthodes

Questionnaire électronique administré aux immigrants guinéens âgés de plus de 18 ans, résidant au Portugal depuis au moins 6 mois. Ils ont été invités à participer à l›étude *via* les réseaux sociaux et par contact direct dans les gares de la région de Lisbonne.

## Résultats

Quatre cent soixante-seize individus ont participé à l’étude, avec un âge moyen de 39,22 ans, et pour 54,8 % des hommes. 40,5 % d’entre eux résident au Portugal depuis plus de 10 ans, 20,4 % depuis 5 à 10 ans. 54,5 % n’ont pas voyagé après avoir immigré. S’ils voyagent, 52,7 % pensent qu’ils pourraient souffrir du paludisme (2/3 à cause de piqûres de vecteurs, 2/5 à cause du manque de prévention, 1/10 à cause du manque d’assainissement dans le pays); 40,5 % pensent qu’ils n’attraperont pas le paludisme (2/5 parce qu’ils sont prudents, 1/6 parce qu’ils ont déjà voyagé et n’ont pas eu la maladie ou parce qu’ils sont nés en Guinée-Bissau). La prévention du paludisme passe pour 83,7 % par des moustiquaires, pour 12 % par la vaccination, pour 12,1 % par la consommation d’eau en bouteille, 0,6 % par l’eau bouillie. Les moyens de prévention sont inconnus pour 3 % d’entre eux.

## Conclusions

Plus de la moitié des participants admettent qu’ils pourraient contracter le paludisme lors d’un voyage dans leur pays d’origine. La majorité reconnaît la moustiquaire comme moyen de prévention, mais environ 15 % ne connaissent pas ou ont des connaissances incorrectes sur la forme de prévention. Une éducation sanitaire est nécessaire dans cette communauté pour réduire les risques lors de futurs voyages.

# Research article: N° 56 Caractérisation des accès palustres à *Plasmodium* non *falciparum* sur 10 ans au Groupe hospitalouniversitaire (GHU) Paris Seine-Saint-Denis, comparaison à des accès palustres à *Plasmodium falciparum*

**Published:** 2024-06-13

**Authors:** Victor FRANCHI, Anthony MARTEAU, Nicolas VIGNIER

**Affiliations:** Service de maladies infectieuses et tropicales, Hôpital Avicenne, AP-HP, Bobigny, France

## Introduction

Le paludisme d’importation à *Plasmodium* non *falciparum (Pnf)* est réputé associé à une moindre sévérité que les cas liés au *Plasmodium falciparum (Pf).* Les recommandations sanitaires pour les voyageurs 2023 du Haut Conseil de la santé publique (HCSP) limitent les indications de chimioprophylaxie dans les régions où *Pf* est absent. Nous nous interrogeons sur les caractéristiques des cas de paludisme d’importation grave à *Pnf* dans notre centre, ainsi que sur les facteurs de risque associés.

## Méthodes

Étude rétrospective cas-témoin 1 :1 sur 10 ans de 2013 à 2022, monocentrique (Groupe hospitalo-universitaire Paris Seine-Saint-Denis). 71 patients admis aux urgences ou hospitalisés pour un paludisme à *Pnf* sont inclus, comparés à 71 patients pris en soins dans les mêmes circonstances pour un paludisme à *Pf,* sans critère d’appariement. Le paludisme grave est défini selon les critères OMS.

## Résultats

Comparément aux paludismes à *Pf,* les paludismes à *Pnf* impliquent plus fréquemment des migrants primo-arrivants, qui présentent un score de comorbidité de Charlson supérieur, une fréquence d’obésité plus faible, une parasitémie plus faible, une fréquence inférieure des cas de paludisme grave *(Pnf* 4/71 *vs Pf* 17/71). Les quatre cas de paludisme grave à *Pnf* sont : une hyperbilirubinémie isolée, une décompensation diabétique avec confusion, deux collapsus cardiovasculaires chez des migrants primoarrivants du sous-continent Indien et présentant un paludisme à *P. vivax*. Parmi les *Pnf, Plasmodium vivax* est significativement associé à la gravité (OR 14,4).

## Conclusion

Les paludismes graves à *Pnf* sont peu fréquents et impliquent des migrants primo-arrivants ou des voyageurs ayant séjourné en zone soumise à indication de chimioprophylaxie.

# Research article: N° 57 Performances diagnostiques de l'I-test^®^ dans la détection des antigènes plasmodiaux. Étude réalisée à l'Institut Pasteur de Tunis

**Published:** 2024-06-13

**Authors:** Emma SIALA, Wafa AISSI, Nada BOULEHMI, Karim AOUN, Aida BOURATBINE

**Affiliations:** 1 Laboratoire de parasitologie-mycologie, Institut Pasteur de Tunis, Tunisie; 2 Centre national pour la promotion de la transplantation d’organes, Tunis, Tunisie

## Introduction

De nombreux tests pour le diagnostic rapide (TDR) du paludisme sont actuellement commercialisés. L’objectif de ce travail était d’évaluer les performances de l’I-test^®^ et de discuter son intérêt dans le diagnostic du paludisme.

## Patients et méthodes

Il s’agit d’une étude prospective réalisée entre janvier 2022 et décembre 2023 au Service de parasitologie de l’Institut Pasteur de Tunis, incluant 106 patients adressés pour suspicion de paludisme et 1 223 sujets asymptomatiques testés dans le cadre du dépistage. Chaque individu a bénéficié d’un prélèvement de sang sur lequel ont été réalisés une goutte épaisse, un frottis sanguin et l’I-test^®^ (Bacterovir laboratoire) détectant l’antigène HRPII spécifique de *Plasmodium falciparum* et l’antigène Aldolase des différents espèces plasmodiales.

## Résultats

Cinquante-six individus se sont révélés infectés par *Plasmodium* sp. (4,21 %). *Plasmodium falciparum* a été l’espèce la plus fréquente (87,5 %). La sensibilité du test était de 80,3 % [69,9-90,8]. Chez les sujets symptomatiques, la sensibilité et la spécificité l’I-test” ont été respectivement de 92,59 % [82,71-100] et de 100 %, et les valeurs prédictives positive et négative de 100 % et de 97,5 % [94,1-100].

Chez les sujets asymptomatiques, le test a détecté 20 cas parmi les 29 infections diagnostiquées par la microscopie soit une sensibilité de 68,9 % [52,1-85,8]. La spécificité était de 99,7 % [99,4-100], la VPP de 86,9 % [73,2-100] et la VPN de 99,2 % [98,8-99,7]. Les faux négatifs du TDR correspondaient tous à des cas d’infections à *P. falciparum* à faibles parasitémies <500 parasites/pl (0,01 %).

## Conclusion

Les performances l’I-test” s’avèrent très limitées dans la détection des faibles parasitémies particulièrement observées chez les porteurs asymptomatiques.

# Research article: N° 58 Consommation de produits à base *d'Artemisia* à visée prophylactique parmi les cas de paludisme d'importation pris en charge au Centre hospitalier universitaire (CHU) de Bordeaux en 2019

**Published:** 2024-06-13

**Authors:** Duc NGUYEN, Pierre NOININ, Anne-Sophie WIET, Éric OUATTARA, Denis MALVY, Alexandre DUVIGNAUD, Thierry PISTONE

**Affiliations:** Unité des maladies tropicales et du voyageur, Service des maladies infectieuses et tropicales, CHU Bordeaux, Bordeaux, France

## Introduction

Le paludisme d’importation demeure un enjeu majeur de santé publique avec environ 5 540 cas déclarés en 2019 en France, dont 396 graves. Des infectiologues ont constaté la consommation de formes non pharmaceutiques *d’Artemisia* en prévention du paludisme. Cette consommation n’est pas dénuée de risque et est formellement déconseillée par les recommandations françaises. L’objectif de ce travail était d’en estimer la prévalence chez les patients pris en charge pour un accès palustre d’importation au CHU de Bordeaux.

## Patients et méthodes

Étude observationnelle rétrospective monocentrique du 1^er^ janvier au 31 décembre 2019 au CHU de Bordeaux.

## Résultats

32 patients avec un accès palustre d’importation ont accepté de participer. L’âge médian était de 39 ans (IIQ = 29-51) dont 18 (56 %) hommes. L’accès palustre survenait après un séjour en Afrique subsaharienne dans tous les cas. L’espèce la plus fréquemment identifiée était *Plasmodium falciparum *(n = 30; 93 %). Neuf cas (28,1 %) présentaient un accès palustre grave. Au total, 28 (87,5 %) ont été initialement traités par pipéraquine/artéminol et 4 (12,5 %) par artésunate. Deux patients (6 %, IC95 % = 2 % - 16 %) ont rapporté avoir consommé de l’A. *annua* à visée prophylactique. Aucun n’est décédé.

## Conclusion

Cette étude pilote suggère que la consommation prophylactique de produit à base *d’Artemisia* chez les voyageurs présentant un accès palustre d’importation n’est pas un phénomène exceptionnel. Une étude de plus grande ampleur serait nécessaire afin d’en estimer plus précisément la prévalence, chez les voyageurs impaludés mais aussi chez tous ceux qui se rendent en zone d’endémie, ainsi que pour en identifier les déterminants.

# Research article: N° 60 Place de la PCR en temps réel dans le diagnostic du paludisme. Étude réalisée à l'Institut Pasteur de Tunis

**Published:** 2024-06-13

**Authors:** Emna SIALA, Hanen CHELBI, H. DRIDI, Nada BOULEHMI, Aida BOURATBINE, Karim AOUN

**Affiliations:** Service de parasitologie, Institut Pasteur de Tunis, Tunisie

## Introduction

L’examen microscopique est la technique de référence pour le diagnostic du paludisme. Cependant, ses performances sont étroitement liées à l’expérience du biologiste et à la parasitémie. L’objectif de ce travail est d’évaluer l’apport d’une technique de PCR en temps réel (RT-PCR) dans le diagnostic du paludisme.

## Patients et méthodes

Cette étude a concerné 114 individus adressés à l’Institut Pasteur de Tunis pour le diagnostic du paludisme entre janvier 2020 et février 2023. Ils correspondaient à des étudiants originaires de zones d’endémie palustre adressés dans le cadre du programme national de dépistage des maladies infectieuses, ainsi qu’à des patients cliniquement suspects. Chaque sujet a eu un prélèvement sanguin qui a fait l’objet d’une goutte épaisse, d’un frottis sanguin et d’une RT-PCR. L’amplification génique a été faite en utilisant deux amorces et une sonde ciblant un fragment de la SSUrRNA 18S spécifique du genre *Plasmodium.*

## Résultats

Trente-cinq cas (30,7 %) de paludisme ont été diagnostiqués par l’examen microscopique. Trente-huit prélèvements étaient positifs à la RT-PCR (33,3 %). Les résultats des deux techniques étaient concordants dans 95,6 % des cas. Quatre prélèvements étaient positifs à la RT-PCR alors que l’examen microscopique était négatif. Ils correspondaient à des étudiants asymptomatiques adressés dans le cadre du dépistage. Un prélèvement positif par la microscopie s’est révélé négatif par la RT-PCR. Ce dernier cas avait de très rares trophozoïtes de *Plasmodium* sp. sur la goutte épaisse et correspondait également à un sujet asymptomatique.

## Conclusion

La RT-PCR se confirme performante dans le diagnostic du paludisme. Elle serait particulièrement contributive en cas des faibles parasitémies.

# Research article: N° 63 Épidémiologie de la leishmaniose cutanée au Maroc

**Published:** 2024-06-13

**Authors:** Mouna RAJI, Souad BOUHOUT, Hafida NAOUI, Maryem IKEN, Laila BOUMHIL, Badre Eddine LMIMOUNI

**Affiliations:** Service de parasitologie et mycologie médicale, Hôpital militaire d’instruction Mohammed V, Rabat, Maroc

## Introduction

La leishmaniose cutanée est une infection parasitaire due à un parasite flagellé du genre *Leishmania.* Elle est le plus souvent une maladie zoonotique transmise par la piqûre de phlébotomes. Ces affections sont de distribution mondiale. Elles évoluent cliniquement chez l’homme sous trois formes : viscérale, cutanée et cutanéomuqueuse.

## Patients et méthodes

Il s’agit d’une étude descriptive rétrospective étalée sur une période de cinq ans (2017 à 2022), menée par le ministère de la Santé du Maroc dans le cadre du programme national de lutte contre les leishmanioses.

## Résultats

La répartition géographique de la maladie n’était pas uniforme à travers le royaume : 60 % des cas au niveau de la région de Draa-Tafilalet avec prédominance de *L. major,* 12 % au niveau de Marrakech-Safi avec prédominance de *L. tropica,* tandis que *L. infantum* était majoritairement retrouvée dans le nord du Maroc. Le sexratio masculin/féminin était de 0,88. La population pédiatrique était la plus touchée. Cliniquement, le nombre de lésions variait d’un patient à l’autre. Par ailleurs, le diamètre des lésions pouvait dépasser les 5 cm. La localisation des lésions était de 51 % au niveau des membres inférieurs et supérieurs tandis que 22 % des lésions étaient localisées au niveau du visage.

## Conclusion

Durant cette période, on a observé une baisse de l’incidence annuelle de la leishmaniose cutanée qui est due en partie à la mise en place d’une stratégie de lutte contre les leishmanioses.

# Research article: N° 64 Aspects cliniques et thérapeutiques de cas suspects et confirmés de leishmaniose cutanée au Mali

**Published:** 2024-06-13

**Authors:** Romain BLAIZOT, Abdoulaye KONE, Daouda SAMAKE, Adama DICKP, Ramata FOFANA, Mohamed LAMINE ALHOUSSEINI, Amadou NIANGALY, Safiatou DOUMBO NIARE, Drissa COULIBALY, Karim TRAORE, Issiaka SAGARA, Abdoulaye DJIMDE, Magalie DEMAR, Alexandre DUVIGNAUD, Ousmane FAYE, Mahamadou Ali THERA

**Affiliations:** Service de dermatologie, Centre hospitalier de Cayenne, Cayenne, Guyane, France

## Introduction

Des études ponctuelles ont été menées sur la leishmaniose cutanée (LC) au Mali mais il existe un défaut de diagnostic pérenne et de disponibilité du traitement. Le but de ce travail était de décrire les aspects cliniques et thérapeutiques de cas confirmés de LC au Mali.

## Patients et méthodes

Une collaboration multicentrique a été mise en place afin de renforcer la formation à la microscopie à Bamako, créer un circuit de PCR du gène Hsp70 sur écouvillons cutanés adressés au Centre hospitalier de Cayenne, et rendre disponible de l’antimoniate de méglumine. Les patients étaient revus entre un et six mois après traitement.

## Résultats

Trente-sept patients présentaient des lésions compatibles. La microscopie était positive chez 22 patients (59,5 %). La PCR était disponible pour 25 patients et positive dans 17/25 (68 %) cas. Le séquençage était possible pour 12 patients et retrouvait *Leishmania major.* Les patients étaient surtout des hommes (34/37, 91,9 %), l’âge moyen de 29 ans, les lésions étaient ulcérées dans 30/37 (81,1 %) des cas. Les patients avaient utilisé des antibiotiques oraux (20/37, 54,1 %), des soins locaux (11/37, 29,7 %) ou des remèdes traditionnels (4/32, 12,5 %), sans succès. De plus, 32/37 recevaient le traitement antimonié, dont 24/32 (75 %) présentaient une guérison clinique. Tandis que 5/37 recevaient des antibiotiques, dont 1/5 (20 %) présentait une guérison. À trois mois, 90 % (n = 35) des patients présentaient une rançon cicatricielle encore visible.

## Conclusion

La PCR sur écouvillon permet une confirmation diagnostique de la LC au Mali. L’antimoniate de méglumine présente un intérêt thérapeutique clair.

# Research article: N° 66 Tendances épidémiologiques et cliniques des leishmanioses viscérales et cutanées au Portugal : analyse rétrospective des cas diagnostiqués dans les hôpitaux publics entre 2010 et 2020

**Published:** 2024-06-13

**Authors:** Rafael ROCHA, Cláudia CONCEIÇÂO, Luzia GONÇALVES, Ana Cláudia CARVALHO, André MAIA, André MARTINS, Antonio CARUJO, Antonio MAIO, Catarina FORRA, Catarina MELITA, Daniela COUTO, Diana FERNANDES, Dulce PEREIRA, Ema LEAL, Helena SARMENTO, Inês SOUSA, Jean-Pierre GONÇALVES, Joana MARINHO, Joana VASCONCELOS, Joâo CUNHA, Joâo RODRIGUES, José Miguel SILVA, Lidia CALEY, Luis MALHEIRO, Luis SANTOS, Margarida GARCIA, Margarida PRATA, Maria CUNHA, Maria LIMA, Maria Margarida ANDRADE, Marta MARQUES, Miguel ALPALHAO, Monica SILVA, Rita FERRAZ, Rui SOARES, Salomâo FERNANDES, Samuel LLOBET, Sofia CRUZ, Teresa GUIMARAES, Tiago BRANCO, Tomás ROBALO-NUNES, Vasco ALMEIDA, Carla MAIA

**Affiliations:** Instituto de higiene e medicina tropical (IHMT), Universidade Nova de Lisboa (UNL), Rua da Junqueira N°100, 1349-008 Lisboa, Portugal

## Introduction

Les leishmanioses sont des parasitoses à protozoaires du genre *Leishmania.* Au Portugal, où *L. infantum* est endémique, seule la leishmaniose viscérale (LV) fait l’objet d’une déclaration obligatoire. Cette étude vise à décrire les cas de leishmaniose diagnostiqués dans les hôpitaux du Service national de santé portugais (SNS) de 2010 à 2020.

## Patients et méthodes

Les cas ont été sélectionnés *via* des codes de diagnostic de décharge hospitaliers. Les données sociodémographiques et cliniques ont été extraites par revue rétrospective. Des modèles descriptifs et tests d’hypothèses ont été réalisés avec IBM^®^ SPSS^®^ Statistics.

## Résultats

Deux cent vingt et un cas de LV, 46 cas de leishmaniose cutanée (LC) et 6 cas de leishmaniose des muqueuses ont été identifiés. Une augmentation de l’incidence nationale estimée a été observée dans les années suivant 2016 (de 0,11 à 0,22 cas pour 100 000 habitants). La LV était principalement diagnostiquée chez les personnes vivant avec le VIH (PVVIH) et les enfants, mais l’évolution clinique était moins favorable en cas d’immunosuppression non liée au VIH. Des présentations atypiques ont concerné 8,5 % des cas de LV. Seuls 49,7 % des cas incidents de LV ont été signalés aux autorités de santé publique. L’incidence de LC était inférieure à celle de LV au niveau national, avec 28,3 % des patients présentant une LV simultanée.

## Conclusion

Au Portugal, les leishmanioses constituent encore un problème de santé important, en particulier pour les PVVIH et les enfants, ainsi qu’une menace croissante pour d’autres groupes immunodéprimés. Les programmes de lutte devraient promouvoir un diagnostic précoce, la réduction de la sous-déclaration, et la surveillance intégrée selon l’approche « One Health ».

# Research article: N° 67 Prévalence de l'infection asymptomatique à *Leishmania* et connaissances, perceptions et pratiques chez les donneurs de sang au Portugal continental

**Published:** 2024-06-13

**Authors:** Rafael ROCHA, Luzia GONÇALVES, Cláudia CONCEIÇÂO, Patricia ANDRADE, José Manuel CRISTÖVÄO, Jorge CONDEÇO, Beatriz DELGADO, Cristina CAEIRO, Tetyana KUZMENKO, Eugénia VASCONCELOS, Maria Antonia ESCOVAL, Carmen REY, Madalina GUZ, Cláudia NORTE, Carlos ALDEIA, Diego CRUZ, Carla MAIA

**Affiliations:** Instituto de higiene e medicina tropical (IHMT), Universidade Nova de Lisboa (UNL), Rua da Junqueira N°100, 1349-008 Lisboa, Portugal

## Introduction

Dans la région méditerranéenne, les études sur l’infection asymptomatique par *Leishmania* utilisent fréquemment des tests sérologiques chez les donneurs de sang. En Espagne, la séroprévalence régionale oscille entre 1 et 8 %, tandis que deux études portugaises suggèrent des valeurs de 0 à 2 %. Notre étude visait à estimer la prévalence de l’infection asymptomatique par *Leishmania* chez les donneurs de sang au Portugal continental et à examiner l’association avec les facteurs sociodémographiques, ainsi que les connaissances, perceptions et pratiques (CPP) relatives à la leishmaniose.

## Patients et méthodes

Une étude transversale a ciblé les donneurs de sang du Portugal continental, entre février et juin 2022, dans 347 points de collecte de sang. Les participants ont rempli un questionnaire sociodémographique et CPP, et la détection des anticorps a été réalisée par ELISA.

## Résultats

201 échantillons positifs sur 3 763 ont été obtenus. La séroprévalence nationale estimée était de 4,8 % (IC à 95 % : 4,1-5,5 %). 72,3 % des participants étaient au courant de la leishmaniose, et dans l’analyse multivariée, un score de connaissance plus élevé était associé à l’âge de 25 à 40 ans, au sexe féminin, à la possession de chiens et à un niveau d’éducation plus élevé. Les modèles montrent une séropositivité plus élevée chez les hommes, les personnes de plus de 25 ans ou vivant dans la région Centro, sans association significative avec la possession de chiens ou des scores CPP plus faibles.

## Conclusion

La séroprévalence estimée (4,8 %) concorde avec des études régionales en Espagne. Des variations régionales pourraient résulter de déplacements géographiques des parasites liés au changement climatique, nécessitant des recherches intégrées selon l’approche « One Health ».

# Research article: N° 68 Leishmaniose cutanée à *L. tropica* chez des élèves des écoles primaires du district sanitaire de Tchiamba-Nzassi à Pointe, République du Congo

**Published:** 2024-06-13

**Authors:** Géril SÉKANGUÉ OBILI, Diane BIDOUNGA, Bienvenu Rolland OSSIBI IBARA, Anicet BOUMBA, Aladin ATANDI, Freddy POUKI, Christy NGANGA, Tatia DOUKAGA, Martin EKAT, Donatien MOUKASSA

**Affiliations:** 1 Faculté des sciences de la santé, Université Marien Ngouabi, 1 Avenue des premiers jeux africains, Diata, Brazzaville, République du Congo; 2 Fondation Marie Madeleine GOMBES(FMMG), 28 rue François Charles, Pointe Noire, République du Congo

## Introduction

La leishmaniose est une maladie tropicale négligée causée par la présence dans l’organisme de *Leishmania* qui peut se manifester par des lésions cutanées. L’objectif de cette étude était de contribuer au diagnostic moléculaire de la leishmaniose cutanée chez des élèves des écoles primaires présentant des lésions suspectes.

## Méthodologie

Il s’est agi d’une étude descriptive transversale qui s’est déroulée de juin à novembre 2022, incluant 23 cas suspects de leishmaniose cutanée chez des élèves des écoles primaires de Tchiamba-Nzassi à Pointe noire. Des frottis colorés au MGG des lésions suspectes et une étude moléculaire par PCR en temps réel à partir d’ADN extrait des produits pathologiques ont été utilisés. Les variables étudiées ont été sociodémographiques, cliniques, parasitologiques et moléculaires.

## Résultats

L’âge moyen des patients était de 11,7 ± 2 ans avec des extrêmes de 8 et 15 ans. Le sex-ratio (H/F) était de 1,87 en faveur des hommes. L’examen parasitologique de tous les frottis a été négatif. Trois patients sur 23 ont été confirmés atteints de leishmaniose cutanée. L’espèce *Leishmania tropica* a été celle qui a été retrouvée après amplification. Deux patients avaient des lésions siégeant au niveau des jambes et un en avait au niveau des orteils.

# Research article: N° 69 Développement d'un test de diagnostic universel des leishmanioses humaines. Potentiel de peptides parasitaires

**Published:** 2024-06-13

**Authors:** Julie PAGNIEZ, Élodie PETITDIDIER, Rachel BRASGONÇALVES

**Affiliations:** UMR 177 – INTERTRYP, Institut de recherche pour le développement (IRD), Montpellier, France

Les leishmanioses sont des infections parasitaires complexes, avec une vingtaine d’espèces pathogènes pour l’homme, et responsables de plusieurs formes cliniques ayant des effets directs sur la santé et le bien-être humain. Plus d’un milliard de personnes sont exposées au risque d’infection dans 98 pays d’endémie, dont 71 présentent plusieurs espèces de *Leishmania* en circulation. Une prise en charge clinique efficace dépend essentiellement d’un diagnostic précoce et précis. Des outils de diagnostic complets aideraient les cliniciens à prendre des décisions éclairées sur le traitement le plus approprié, en particulier dans les pays où circulent plusieurs espèces de leishmanies.

Notre étude a pour objectif d’identifier des biomarqueurs peptidiques parasitaires afin de développer un test de sérodiagnostic à la fois générique pour détecter toutes les infections à *Leishmania* et spécifique à l’espèce pour identifier avec précision l’agent pathogène impliqué.

À partir d’une étude précédente du sécrétome de six espèces de *Leishmania,* une bibliothèque de peptides contenant des épitopes de cellules B linéaires a été prédite *in silico* par des outils bioinformatiques. Au total, 61 peptides communs et 106 peptides spécifiques ont été synthétisés.

Par la suite, des échantillons de sérum issus de patients atteints de différentes formes de leishmaniose seront utilisés pour évaluer le potentiel diagnostique des peptides et sélectionner les plus performants. Dans une vision « One Health » de lutte contre les leishmanioses, ce test pourrait être adapté à d’autres hôtes, tels que les chiens.

# Research article: N° 70 Persistance de réservoirs canins et risque humain de leishmaniose dans les Bouches-du-Rhône et les départements voisins

**Published:** 2024-06-13

**Authors:** Younes LAIDOUDI, Jacques SEVESTRE, Samia BEDJAOUI, Stéphanie WATIER-GRILLOT, Bernard DAVOUST

**Affiliations:** Institut hospitalo-universitaire (IHU) Méditerranée Infection, Marseille, France

## Introduction

La leishmaniose, causée par *Leishmania infantum,* persiste dans le sud de la France, avec une saison de transmission par les phlébotomes prolongée en raison du réchauffement climatique.

## Patients et méthodes

Une femme de 76 ans, habitant à Marseille et ne voyageant pas, a été atteinte de leishmaniose cutanée à *L. infantum,* début 2023. Des phlébotomes infectés sur des chiens réservoirs de parasites sont, potentiellement, à l’origine de cette parasitose. La séroprévalence (ELISA, ID Screen^®^ Leishmaniasis Indirect, Innovative Diagnostics, Grabels, France) de la leishmaniose canine a été évaluée, de mai à octobre 2023, dans cinq départements proches de Marseille.

## Résultats

L’enquête a porté sur 710 chiens vivant dans 17 localités. Elle révèle une séroprévalence moyenne de 4,5 %, variant ainsi : Var 1,5 %, Hérault 2 %, Bouches-du-Rhône 3,3 %, Gard 5,9 % et Vaucluse 11,9 %. Parmi les 11 chiens préalablement diagnostiqués positifs et traités, 73 % sont restés séropositifs. Chez les chiens séropositifs, 50 % sont asymptomatiques. Le taux de prévalence de la leishmaniose canine clinique est de 2,25 %.

## Conclusion

À Marseille (séropositivité 6 %), des chiens réservoirs de *L. infantum* contribuent, potentiellement, à l’émergence de cas humains. La proximité de chiens positifs avec le cas humain décrit et l’identification de phlébotomes vecteurs infectés dans le quartier de la patiente ont confirmé ce risque. Les Cévennes et le Vaucluse restent des foyers actifs. Les enquêtes sérologiques canines sont utiles pour la surveillance épidémiologique de la leishmaniose humaine et animale.

# Research article: N° 71 Dépistage de la bilharziose chez les migrants mineurs non accompagnés au Centre de lutte antituberculeuse des Alpes Maritimes

**Published:** 2024-06-13

**Authors:** Frédéric VANDENBOS, Flore PETIT, Victoria BUNGER, Julie BERNARDESCHI, Christelle PIERRAT, Pascal DELAUNAY, Karine RISSO

**Affiliations:** CLAT 06, 30 voie Romaine, 06001 Nice Cedex 1, France

## Introduction

Le dépistage de la bilharziose n’est pas une mission prioritaire des Centres de lutte antituberculeuse (CLAT) chez les migrants. Cependant, l’épidémie de bilharziose urinaire survenue en Corse en 2013 suscite la question d’un dépistage.

## Patients et méthodes

Le dépistage de la bilharziose par sérologie (Western Blot) est systématiquement proposé aux migrants mineurs non accompagnés (MNA) au CLAT de Nice depuis la fin novembre 2023. Une recherche d’œufs parasitaires dans les selles et dans les urines est secondairement proposée.

## Résultats

Entre le 28 novembre et le 28 décembre 2023, 70 MNA ont consulté au CLAT de Nice et 69 d’entre eux ont accepté le dépistage de la bilharziose. L’âge moyen de la population était de 15,5 ans avec un sexratio H/F de 3,6. Les pays d’origine de ces MNA étaient : la Côte d’Ivoire (27), la Guinée (26), la Tunisie (8) et autres (Mali = 3, Cameroun = 2, Somalie = 1, Soudan = 1, Tchad = 1). La sérologie de la bilharziose était positive dans 43 (62 %) des cas. La fréquence par pays de sérologie bilharziose positive chez ces jeunes était 67 % en Côte d’Ivoire, 77 % en Guinée, 0 % en Tunisie et 62,5 % pour les autres pays. À ce jour, la recherche d’œufs parasitaires a été effectuée pour huit MNA et positive pour sept d’entre eux.

## Conclusion

La séroprévalence de la bilharziose chez les MNA d’Afrique subsaharienne était de 70 %.

# Research article: N° 74 Le taux de la séroconversion toxoplasmique pendant la grossesse dans l'est algérien

**Published:** 2024-06-13

**Authors:** Nabila KALLA, Ouanassa HAMOUDA, Asma ABDESSAMAD, Achouak BELHADI, Amina AYADI, Ibtihal AOUADJ, Zohra MERZOUGUI, Souhila AOUIDANE, Nora RIGHI

**Affiliations:** Faculté de médecine, Université Batna 2, Batna, Algérie

## Introduction

En général, la toxoplasmose n’a pas de conséquences graves, mais sa survenue au cours de la grossesse expose au risque de la toxoplasmose congénitale, mettant en jeu le pronostic fœtal et néonatal. L’objectif principal de cette étude est d’établir l’incidence de la séroconversion toxoplasmique pendant la grossesse.

## Patients et méthodes

Il s’agit d’une étude transversale descriptive menée à la maternité de la ville de Batna auprès des femmes enceintes à terme de leurs grossesses. Cette étude a été réalisée sur une période de trois mois allant de décembre 2022 à février 2023. Toutes les gestantes ont eu une sérologie toxoplasmique (IgM/IgG).

## Résultats

Durant la période d’étude, nous avons recruté 511 gestantes dont l’âge moyen est de 29 ans, avec des extrêmes allant de 18 à 47 ans. La majorité des femmes est multipare (65,9 %). La sérologie de toxoplasmose a été réalisée de façon systématique chez la plupart des gestantes au cours du premier trimestre soit pour 68,2 % d’entre elles. Seulement 1,5 % l’ont fait lors de leur dernier trimestre de grossesse. Sur l’ensemble des sérologies toxoplasmiques qui ont été réalisées, 54,6 % des gestantes avaient une sérologie négative et 45,4 % une sérologie positive. Parmi toutes les femmes enceintes séronégatives, seulement deux (0,72 %) femmes ont fait une séroconversion.

## Conclusion

La prévention de la toxoplasmose congénitale est basée sur le dépistage, la sensibilisation aux risques potentiels et l’éducation, ainsi que la surveillance régulière du taux d’anticorps chez les femmes qui n’ont pas été infectées par la toxoplasmose.

# Research article: N° 77 Kyste hydatique de localisation atypique : à propos d'une série de cas dans l'Unité de soins intensifs de Sfax

**Published:** 2024-06-13

**Authors:** Sana KHARRAT, Karama BOUCHAALA, Sabrine BRADAI, Rihab BOUBTANE, Rania AMMAR, Chokri BEN HAMIDA, Mabrouk BAHLOUL, Mounir BOUAZIZ

**Affiliations:** Réanimation médicale, Centre hospitalier universitaire (CHU) Habib Bourguiba, Sfax, Tunisie

## Introduction

Le kyste hydatique est une affection encore endémique en Tunisie. Il peut toucher tous les organes, mais préférentiellement le foie et le poumon.

## Matériel et méthodes

Nous rapportons une petite série de cas rares de kystes hydatiques de localisation exceptionnelle. Tous les cas sont issus du milieu rural avec une notion de contact avec les chiens. Le premier cas est une femme de 21 ans aux antécédents de kyste hydatique du foie opéré, qui consulte pour paraplégie d’installation progressive avec un niveau sensitif à droite.

## Résultats

L’imagerie par résonance magnétique a montré un aspect d’une échinococcose osseuse d’origine hydatique de la vertèbre D3 avec extension endo-canalaire centrale, compression du cordon médullaire et extension paravertébrale médiastinale gauche Le 2^e^ cas est celui d’une femme de 40 ans avec dyspnée évoluant depuis deux mois, et qui consulte pour détresse respiratoire avec douleur thoracique atypique. Une tomodensitométrie a été réalisée montrant multiples kystes hydatiques pulmonaires avec une découverte d’un kyste hydatique cardiaque au niveau de la paroi libre du ventricule droit. Le 3^e^ cas concerne une fillette de cinq ans qui consulte pour des convulsions avec à l’imagerie des kystes hydatiques intracérébraux. Les trois patients ont eu des interventions chirurgicales avec une évolution favorable.

## Conclusion

Les localisations atypiques demeurent de diagnostic souvent tardif avec des conséquences économiques et sociales lourdes. En pays endémique, une origine hydatique doit être toujours évoquée devant des opacités kystiques dont l’étiologie n’est pas précisée.

# Research article: N° 78 Mucormycose et diabète dans le centre tunisien : à propos de 23 cas

**Published:** 2024-06-13

**Authors:** Alia YAACOUB, Meriem AJMI, Mouna BELAKHDHER, Samar ISMAIL, Imen KHAMMARI, Hamed CHOUAIEB, Mohamed ABDELKÉFI, Akila FATHALLAH

**Affiliations:** 1 Laboratoire de parasitologie-mycologie, Hôpital Farhat Hached, Sousse, Tunisie; 2 Service d’oto-rhino-laryngologie et de chirurgie cervico-faciale, Hôpital Farhat Hached, Sousse, Tunisie

## Introduction

La mucormycose, infection fongique rare et rapidement extensive, touche principalement les diabétiques, notamment en acidocétose. L’objectif de notre étude est de décrire les caractéristiques épidémiologiques, cliniques, mycologiques, histopathologiques, évolutives et thérapeutiques de la mucormycose au cours du diabète dans le centre tunisien.

## Patients et méthodes

Il s’agit d’une étude rétrospective portant sur 23 cas de mucormycose diagnostiqués chez des diabétiques pendant 30 ans (janvier 1992 - août 2022) au service de parasitologie-mycologie du CHU Farhat Hached de Sousse. Le diagnostic a été confirmé par l’examen mycologique et/ou histopathologique des tissus nécrosés.

## Résultats

Il s’agissait de 13 hommes et 10 femmes, d’âge moyen de 48 ans (extrêmes : 16-77 ans). Parmi les patients diabétiques, 10 ont présenté une acidocétose. La mucormycose était rhinocérébrale (17 cas), pulmonaire (4 cas), cutanée (1 cas) et auriculaire (1 cas). Le diagnostic de mucormycose a été confirmé par l’histopathologie chez 20 patients. La culture a isolé des mucorales du genre *Rhizopus* dans 17 cas *(Rhizopus arrhizus* : 13, *Rhizopodiformis* : 1, *Rhizopus* sp. : 3), le genre *Lichtemia* dans trois cas et *Rhizomucor pusillus* dans un cas. Parmi les 21 patients traités, 20 ont reçu un traitement antifongique (combiné à la chirurgie : 12 cas) et le dernier patient a seulement bénéficié d’un débridement chirurgical. L’évolution a été marquée par le décès de 13 patients.

## Conclusion

La mucormycose chez les diabétiques est grevée d’une lourde mortalité. Un diagnostic rapide et un traitement précoce, combinant antifongiques et débridement chirurgical, garantissent un meilleur pronostic.

# Research article: N° 79 La mucormycose rhinocérébrale d'origine dentaire chez les patients diabétiques : à propos de quatre cas

**Published:** 2024-06-13

**Authors:** Meriem AJMI, Alia YAACOUB, Mouna BELLAKHDHAR, Abir MEHERZI, Samar ISMAIL, Hamed CHOUAIB, Imene KHAMMARI, Mohamed ABDELKEFI, Akila FATHALLAH

**Affiliations:** 1 Laboratoire de parasitologie-mycologie, Hôpital Farhat Hached, Sousse, Tunisie; 2 Service d’oto-rhino-laryngologie et de chirurgie cervico-faciale, Hôpital Farhat Hached, Sousse, Tunisie

## Introduction

La mucormycose rhinocérébrale, infection fongique souvent mortelle, touche principalement les immunodéprimés, notamment les diabétiques. D’origine dentaire, elle est décrite essentiellement suite à une extraction dentaire et plus rarement après une infection dentaire. L’objectif de notre étude est de décrire les particularités épidémiologiques, cliniques, mycologiques, thérapeutiques et évolutives de la mucormycose d’origine dentaire dans le centre tunisien.

## Matériels et méthodes

Il s’agit d’une étude rétrospective portant sur quatre cas de mucormycose rhinocérébrale d’origine dentaire colligés pendant 30 ans au service de parasitologie-mycologie du CHU Farhat Hached de Sousse. Le diagnostic de mucormycose a été confirmé par l’examen mycologique et/ou histopathologique des biopsies.

## Résultats

Il s’agissait de trois hommes et d’une femme, d’âge moyen de 43 ans. La mucormycose rhinocérébrale a résulté d’une infection dentaire (abcès ou parodontite) dans deux cas, d’une extraction dentaire dans un cas et de soins dentaires dans un autre cas. L’examen direct, positif dans les quatre cas, a révélé des filaments mycéliens non cloisonnés avec des ramifications à angles droits. La culture, positive dans trois cas, a isolé *Rhizopus arrhizus* (deux cas) *et Rhizomucor pusillus* (un cas). L’examen anatomopathologique a confirmé le diagnostic dans trois cas. L’amphotéricine B prescrite dans tous les cas, a été associée à un débridement chirurgical dans trois cas. L’évolution a été marquée par le décès d’un patient.

## Conclusion

La prise en charge de la mucormycose, particulièrement d’origine dentaire, doit être multidisciplinaire. Le dentiste joue un rôle crucial dans la prévention et le diagnostic précoce de cette affection grave.

# Research article: N° 80 Maladie dermatophytique : à propos de quatre cas tunisiens

**Published:** 2024-06-13

**Authors:** Meriem AJMI, Samar ISMAIL, Douha GHARRABA, Hamed CHOUAIEB, Imen KHAMMARI, Alia YAACOUB, Akila FATHALLAH

**Affiliations:** Service de parasitologie-mycologie, Centre hospitalier universitaire (CHU) Farhat Hached, Sousse, Tunisie

## Introduction

La maladie dermatophytique est une forme rare de dermatophytose cutanéo-viscérale chronique, probablement liée à un déficit de l’immunité cellulaire envers des dermatophytes communs.

## Patients et méthodes

Nous rapportons quatre cas de maladie dermatophytique. Il s’agit respectivement d’un homme de 50 ans, d’un homme de 43 ans, d’un homme de 41 ans et d’une femme de 49 ans. Chaque patient a bénéficié d’un examen clinique minutieux : examen anatomopathologique, examens mycologiques et étude génétique pour étayer le diagnostic.

## Résultats

Ces patients ont partagé des similitudes dans leur présentation clinique : une atteinte cutanée caractérisée par des lésions érythémato-squameuses généralisées et des papulo-nodules; une atteinte unguéale à type de pachyonychie et onychodystrophie. L’examen mycologique des différentes lésions a révélé la présence de *Trichophyton violaceum* et/ou de *Trichophyton rubrum.* L’examen anatomopathologique réalisé dans trois cas, a montré la présence de granulomes épithélioïdes et giganto-cellulaires centrés par des filaments mycéliens PAS et Gomori-Grocott positifs. Une étude génétique approfondie a révélé un déficit autosomique récessif en CARD9 (protéine contenant le domaine de recrutement de caspase 9) dans deux cas. L’évolution était marquée par la survenue de plusieurs poussées de dermatophytie généralisée malgré l’utilisation de différents antifongiques dans trois cas tandis que l’évolution sous traitement antifongique était favorable dans un seul cas.

## Conclusion

Malgré l’apparition de nouvelles molécules antifongiques et immunostimulantes, la dermatophytose reste une maladie grave, engageant le pronostic vital après des années d’évolution. Un diagnostic précoce et une surveillance régulière et prolongée s’imposent.

# Research article: N° 81 Prévalence des teignes du cuir chevelu chez les enfants d'âge scolaire dans trois villages du département du Haut Ntem dans la province du Woleu Ntem, Gabon

**Published:** 2024-06-13

**Authors:** Géril SÉKANGUÉ OBILI, Roger Hadry SIBI MATOTOU, Noé Patrick M’BANDOUKWE, Jacques Mari NDONG NGOMO, Meredith Flore ADA MENGOME, Lucherri NDONG AKOMZOGHE, Charlène MANOMBA, Aesène MOUNOMBY, Denise Patricia MAWIlI MBOUMBA, Solange NZENZE AFENE, Marielle Karine BOUYOU AKOTET

**Affiliations:** 1 Faculté des sciences de la santé, Université Marien Ngouabi, 1 Avenue des premiers jeux africains, Diata, Brazzaville, République du Congo; 2 Université des Sciences de la Santé, Boulevard de l’indépendance, Owendo, Libreville, République du Gabon

## Introduction

Les teignes du cuir chevelu sont des infections fongiques alopéciantes bénignes atteignant principalement les enfants d’âge scolaire avec une tendance à la guérison spontanée à la puberté. Elles sont dues aux dermatophytes. L’objectif était d’étudier la prévalence des teignes du cuir chevelu chez les enfants d’âge scolaire du département du Haut Ntem.

## Méthodologie

Il s’est agi d’une étude transversale descriptive qui s’est déroulée du 11 novembre 2021 au 7 avril 2022 dans trois villages du département du Haut Ntem. Des prélèvements du cuir chevelu ont été réalisés chez des enfants d’âge scolaire à l’aide d’un scalpel et recueillis dans une boîte de Pétri. Chaque échantillon a fait l’objet d’un examen direct et d’une culture sur milieu Sabouraud Chloramphénicol Actidione. Le diagnostic de teigne du cuir chevelu était retenu en cas de positivité de l’examen direct ou de la culture.

## Résultats

Cent vingt-huit enfants ont été inclus. L’âge médian était de neuf ans. Les teignes du cuir chevelu à petite plaque (66,4 %) ont été plus fréquentes que celles à grande plaque (33,6 %). La fréquence des teignes du cuir chevelu a été plus élevée à l’examen direct (23,6 %) qu’à la culture (9,4 %). La tranche d’âge la plus touchée par les teignes, par constat par examen direct ou par culture, était celle des 6-9 ans (p = 0,27). Les garçons (24,3 %) ont été significativement plus touchés que les filles. Après culture, les teignes du cuir chevelu ont été plus fréquentes à Gabon-ville (41,7 %), alors que leur fréquence (25,0 %) était similaire à Akok et Gabon ville à l’examen direct. Le parasitisme endothrix était significativement plus fréquent (p<0,0001). Les teignes du cuir chevelu étaient essentiellement dues au genre *Trichophyton* (100 %, p<0,0001).

## Conclusion

Les teignes du cuir sont un problème de santé publique en zone rurale au Gabon dues aux champignons du genre *Trichophyton.*

# Research article: N° 83 Épidémiologie des infections à *Fusarium* sur 9 ans au Centre hospitalier universitaire (CHU) de Nice

**Published:** 2024-06-13

**Authors:** Hajer CHOURA, Loïc SIMON, Christelle POMARES, Lilia HASSEINE

**Affiliations:** Service de parasitologie mycologie, CHU de Nice, Hôpital Archet de Nice, France, Nice, France

## Introduction

Les fusarioses sont des mycoses émergentes présentant un polymorphisme clinique avec des formes superficielles, localement invasives ou encore disséminées.

## Objectif

Évaluer la fréquence des infections à *Fusarium* au CHU de Nice.

## Méthodes

Il s’agit d’une étude rétrospective analysant les fusarioses diagnostiquées dans notre laboratoire de janvier 2014 à février 2023. L’identification des isolats de *Fusarium* est basée sur la macroscopie et la microscopie combinée au MALDI TOF (base MSI2).

## Résultats

Au total, 489 isolats de *Fusarium* ont été obtenus chez 341 patients. Sur cet ensemble, 314 patients présentaient des mycoses superficielles. Les onychomycoses étaient les plus fréquentes (245 patients; 63,61 %), suivies par les kératomycoses (53 patients; 31,57 %) et les atteintes cutanées (18 patients; 4,82 %). Seize patients ayant une colonisation respiratoire, présentaient une mucoviscidose. Cinq patients avaient une infection localement invasive. Six patients présentaient une fusariose disséminée. Le complexe d’espèces *Fusarium oxysporum* (FOSC) était le plus fréquent (176 souches; 35,99 %), suivi par le complexe d’espèces *Fusarium fujikuroi* (FFSC) (113 souches; 23,10 %). Le complexe d’espèces *Fusarium solani* (FSSC) était représenté par 96 souches (9,63 %) et enfin les complexes d’espèces *Fusarium dimerum* (FDSC) et *Fusarium incarnatum/equiseti* (FIESC) représentaient cinq cas chacun (1,03 %). FOSC était responsable de la plupart des infections superficielles (39,81 %) alors que FFSC était plus incriminé dans les infections profondes locales et disséminées (71,42 %).

## Conclusion

Les onychomycoses et les kératites à *Fusarium* sont les plus fréquentes. Le *Fusarium* est un agent fongique important à prendre en compte dans la pratique clinique.

# Research article: N° 85 Les envenimations ophidiennes graves : étude épidémio-clinique en milieu de réanimation

**Published:** 2024-06-13

**Authors:** Sabrine BRADAI, Rezk GHORBEL, Sana KHARRAT, Karama BOUCHAALA, Najeh BACCOUCHE, Rania AMMAR, Olfa TURKI, Mabrouk BAHLOUL, Chokri BENHAMIDA, Mounir BOUAZIZ

**Affiliations:** Service de réanimation polyvalente du Centre hospitalier universitaire (CHU) Habib Bourguiba, Sfax, Tunisie

## Introduction

L’envenimation ophidienne (EO) est une maladie tropicale négligée avec une morbi-mortalité importante. L’objectif de notre travail est d’étudier les caractéristiques épidémiologiques et cliniques des EO graves en réanimation.

## Patients et méthodes

Étude rétrospective et descriptive, menée dans notre service de réanimation polyvalente au CHU Habib Bourguiba de Sfax, sur une période de 17 ans (2006- 2023). Nous avons inclus tous les patients victimes de morsure par serpent.

## Résultats

Nous avons inclus 22 patients. Le sex-ratio (H/F) était de 1. L’âge moyen était de 29,4 ± 26 ans (1 à 82 ans). Les EO étaient plus fréquentes entre juin et septembre (86,7 %). Les serpents ont été identifiés par 15 patients (68,2 %). Les localisations des morsures étaient plus fréquentes au niveau des membres supérieurs chez 11 patients (50 %). Le score SOFA moyen était de 4,6 ± 3,3. Selon la classification d’Audebert, 14 patients (63,6 %) avaient une envenimation sévère (Grade III) et 8 patients (36,7 %) une envenimation modérée (Grade II). À l’admission, 5 patients étaient en coma (22,7 %), 5 patients étaient en état de choc (22,7 %), 16 patients avaient un œdème régional (72,7 %) et 5 patients avaient un syndrome de loge (22,7 %). La durée moyenne de séjour était de 8,2 ± 10,5 jours (1-43 j). Le taux de létalité était de 22,7 %.

## Conclusion

Notre étude a démontré que l’EO est une maladie accidentelle grave en Tunisie. L’amélioration du pronostic passe par l’éducation sanitaire et la généralisation des sérums antivenimeux au niveau des hôpitaux de première ligne.

# Research article: N° 86 Les facteurs prédictifs de mortalité par envenimations vipérines graves

**Published:** 2024-06-13

**Authors:** Sabrine BRADAI, Rezk GHORBEL, Karama BOUCHAALA, Sana KHARRAT, Najeh BACCOUCHE, Rania AMMAR, Olfa TURKI, Mabrouk BAHLOUL, Chokri BEN HAMIDA, Mounir BOUAZIZ

**Affiliations:** Service de réanimation polyvalente Centre hospitalier universitaire (CHU) Habib Bourguiba, Sfax, Tunisie

## Introduction

L’envenimation vipérine (EV) est une maladie tropicale grave qui tue plus de 100 000 personnes chaque année dans le monde. En Tunisie, elle est particulièrement fréquente dans les régions subsahariennes. L’objectif de notre étude est d’identifier les facteurs prédictifs de mortalité par EV.

## Matériels et méthodes

Il s’agit d’une étude rétrospective, descriptive et analytique, menée dans notre service de réanimation polyvalente au CHU Habib Bourguiba de Sfax, sur une période de 17 ans (janvier 2006- décembre 2023). Nous avons comparé les patients survivants et les non-survivants victimes d’EV.

## Résultats

Durant la période d’étude, 22 patients ont été inclus. Le sex-ratio (H/F) était de 1. L’âge moyen était de 29,4 ± 26 ans (1 à 82 ans). Le taux de létalité était de 22,7 %. Les facteurs prédictifs de mauvais pronostic étaient : le score SOFA (p = 0,013), le choc index (p = 0,021), la présence d’état de choc à l’admission (p = 0,009), le taux de globules blancs (p = 0,018), le taux de CRP (p = 0,02), l’insuffisance rénale aiguë (p = 0,039), le taux d’urée (p = 0,002) et la présence d’ecchymoses au niveau du membre (p = 0,039).

## Conclusion

L’évaluation précoce de la gravité est cruciale dans la prise en charge des EV. Des études multicentriques nationales sont nécessaires pour approuver un score de gravité propre aux EV en Tunisie.

# Research article: N° 87 Hémisuccinate d'hydrocortisone et envenimation scorpionique grave : impact pronostique (Tunisie)

**Published:** 2024-06-13

**Authors:** Karama BOUCHAALA, Sabrine BRADAI, Sana KHARRAT, Mabrouk BAHLOUL, Chokri BEN HAMIDA, Mounir BOUAZIZ

**Affiliations:** Service de réanimation polyvalente, Centre hospitalier universitaire (CHU) Habib Bourguiba, Sfax, Tunisie

## Objectif

Évaluer l’impact pronostique de l’administration de l’hémisuccinate d’hydrocortisone, chez les enfants admis en unité de soins intensifs pour envenimation scorpionique grave.

## Méthode

Nous avons mené une étude castémoins par paires avec appariement 1:1. Centre quatre-vingt-quatre enfants ont été inclus dans l’étude (92 cas et 92 témoins); les cas ont reçu de l’hémisuccinate d’hydrocortisone pendant leur hospitalisation et les témoins n’ont reçu aucun stéroïde. Les patients ont été appariés en fonction de l’âge (+ 2 ans), des facteurs de gravité à l’admission et de l’administration d’antivenin scorpionique.

## Résultats

Pas de différences significatives entre les deux groupes en termes d’âge (4,9 + 5,5 ans contre 6,2 + 3,8 ans; p 0,05), de température moyenne à l’admission (37,2 + 1,2 contre 37,2 + 1,06; p 1/4 0,99) ou de présence de syndrome de réponse inflammatoire (SIRS) (77 *vs* 70; p 1/4 0,198). La proportion de patients présentant un œdème pulmonaire était similaire dans les deux groupes (77 *vs* 71; p 0,05) et dans chaque groupe, 46 patients (50 %) ont reçu un antivenin de scorpion (p 0,05). Le recours à une assistance respiratoire invasive, la durée du séjour en réanimation et la mortalité en réanimation n’étaient pas significativement différentes entre les groupes étudiés.

## Conclusion

Chez les enfants hospitalisés pour envenimation scorpionique grave, l’administration d’une corticothérapie n’est pas associée à une amélioration pronostique.

# Research article: N° 88 Les facteurs associés à la survenue d'un œdème pulmonaire chez les patients victimes d'une envenimation scorpionique

**Published:** 2024-06-13

**Authors:** Karama BOUCHAALA, Sana KHARRAT, Sabrine BRADAI, Mabrouk BAHLOUL, Chokri BEN HAMIDA, Mounir BOUAZIZ

**Affiliations:** Service de réanimation polyvalente, Centre hospitalier universitaire (CHU) Habib Bourguiba, Sfax, Tunisie

## Introduction

Déterminer les facteurs prédictifs de survenue d’un œdème pulmonaire chez les patients victimes d’une envenimation scorpionique.

## Méthodes

Étude rétrospective sur 13 ans. Ont été inclus dans cette étude 428 patients âgés de plus de trois ans admis en réanimation pour envenimation scorpionique. Les patients ont été classés en deux groupes selon la présence ou l’absence d’œdème pulmonaire évaluée par un comité médical.

## Résultats

L’âge moyen (± SD) était de 17,5 ± 17,7 ans, allant de 3 à 76 ans. Le groupe œdème pulmonaire comprenait 294 patients (68,7 %). Une analyse multivariée a retrouvé les facteurs suivants corrélés à un œdème pulmonaire : âge inférieur à cinq ans (p = 0,04), agitation (p = 0,01), leucocytes de 25 000 cellules/mm^3^ ou plus (p = 0,02) et une protidémie de 72 g/l ou plus (p < 0,0001). Une protidémie supérieure ou égale à 72 g/l permettait de prédire la présence d’un œdème pulmonaire avec une sensibilité de 78 %, une spécificité de 88 %, une valeur prédictive positive de 93 % et une valeur prédictive négative de 64 %. L’association d’une fréquence respiratoire supérieure à 30 c/min, d’agitation et de transpiration clinique, indique la présence d’un œdème pulmonaire avec une spécificité de 84,3 % et une valeur prédictive positive de 87,5 %.

## Conclusion

Chez les patients hospitalisés pour envenimation scorpionique âgés de plus de trois ans, l’association d’une fréquence respiratoire de 30 c/min, d’une agitation, d’une transpiration ou hyper protidémie suggère la présence d’un œdème pulmonaire.

# Research article: N° 90 Punaises de lit : infestations des gares routières à Abidjan et risque de dissémination en Côte d'Ivoire

**Published:** 2024-06-13

**Authors:** Diakaridia FOFANA, Moihet Kouassi Sammuel GEDEON, Jean Michel Vianney BEUGRE, Salifou KONE, Lambert Kouassi KONAN, Kassoum OUATTARA, Yapi Yapi GREGOIRE, Mahama TOURE, Atioumounan Blaise KONE., Daouda COULIBALY, Kouadio Daniel EKRA

**Affiliations:** Service de lutte antivectorielle, ministère de la Santé, de l’hygiène publique et de la couverture maladie universelle, Institut national d’hygiène publique, BP V 14 Abidjan, Côte d’Ivoire

## Introduction

La présente étude dont l’objectif est d’évaluer le niveau d’infestation des gares routières par les punaises de lit a été réalisée dans les communes d’Abobo, Adjamé, Koumassi, Treichville et Yopougon du district autonome d’Abidjan.

## Méthodes

La collecte des punaises dans les gares routières a consisté à rechercher par observation à l’œil nu, les signes de la présence des punaises de lit dans tous les endroits susceptibles de les abriter. Des bombes aérosols ont été utilisées pour débusquer les insectes qui ont été par la suite prélevés, puis conservés dans de l’alcool à 70 %. Les échantillons de punaises ont été identifiés à la loupe binoculaire à l’aide de clés dichotomiques.

## Résultats

Dans les 100 gares routières investiguées, 725 punaises de lit composées de *Cimex hemipterus* et *Cimex lectularius,* ont été collectées sur les bancs des passagers, les sièges des cars, les lits de repos, dans les fissures de murs, les nattes de repos et les tables de bureau. Elles ont été prélevées dans les communes abritant plus de 90 % des gares routières (Abobo, Adjamé et Yopougon). Le taux de co-infestation a été de 85,7 % à Abobo, 89,5 % à Adjamé et 100 *%* à Yopougon. Le niveau d’infestation des punaises variait de 36,4 % à 45,3 %.

## Conclusion

Les infestations de punaises de lit constatées dans les gares routières du district autonome d’Abidjan traduisent un risque de dissémination vers d’autres villes, voire hors des limites de la Côte d’Ivoire.

# Research article: N° 91 Les microangiopathies thrombotiques en réanimation : étude rétrospective de 20 ans dans l'Unité de soins intensifs de Sfax

**Published:** 2024-06-13

**Authors:** Sana KHARRAT, Olfa TURKI, Karama BOUCHAALA, Sabrine BRADAI, Abir DAMMAK, Kamilia CHTARA, Chokri BEN HAMIDA, Mabrouk BAHLOUL, Mounir BOUAZIZ

**Affiliations:** Réanimation médicale, Centre hospitalier universitaire (CHU) Habib Bourguiba, Sfax, Tunisie

## Introduction

La microangiopathie thrombotique (MAT) est une affection rare. Cette pathologie est responsable d’une morbi-mortalité importante. Cependant, seuls quelques rapports sur des patients tunisiens atteints de MAT ont été publiés jusqu’à présent.

## But du travail

Notre but est d’identifier les facteurs associés au mauvais pronostic des malades.

## Matériel et méthodes

Notre étude a été rétrospective, descriptive et analytique, menée dans le service de réanimation. La période d’étude s’est étendue sur 20 ans.

## Résultats

Nous avons colligé 37 malades. L’incidence de MAT en réanimation a été de 0,18 % avec une prédominance féminine et une moyenne d’âge de 34,8 ans. L’anémie hémolytique et la thrombopénie étaient présentes chez la totalité des patients. Les manifestations neurologiques prenaient le devant du tableau clinique (81,1 %). Le purpura thrombotique thrombocytopénique (PTT) occupait les % des formes de MAT de notre cohorte. Les MAT associées à la grossesse ont été la cause prédominante (45,9 %). La prise en charge s’appuyait sur les échanges plasmatiques (70,3 %) en première ligne, en association avec les corticoïdes et les immunoglobulines. Le suivi des patients au cours de l’hospitalisation a souligné un taux d’exacerbations de 18,9 % des cas et des complications comme les infections dans 45,9 % des cas. La mortalité a été de 29,7 %. Les facteurs corrélés à un mauvais pronostic dans notre cohorte ont été l’âge (> 36 ans), le score de Glasgow (< 9) et le taux de plaquettes (< 14 000 elts/mm^3^) à l’admission.

## Conclusion

Les MAT constituent une cause de mortalité encore élevée. Une meilleure connaissance de cette entité, la sensibilisation des médecins et le développement des moyens diagnostiques pourraient améliorer le pronostic.

# Research article: N° 92 La thrombophlébite cérébrale post-traumatique : étude épidémioclinique en réanimation

**Published:** 2024-06-13

**Authors:** Sabrine BRADAI, Kamilia CHTARA, Sana KHARRAT, Najeh BACCOUCHE, Karama BOUCHAALA, Rania AMMAR, Olfa TURKI, Hedi CHELLY, Mabrouk BAHLOUL, Chokri BENHAMIDA, Mounir BOUAZIZ

**Affiliations:** Service de réanimation médicale, Centre hospitalier universitaire (CHU) Habib Bourguiba, Sfax, Tunisie

## Introduction

La thrombophlébite cérébrale post-traumatique (TCPT) est une complication vasculaire rare. L’objectif de notre travail est de déterminer les caractéristiques cliniques, paracliniques, thérapeutiques et évolutives des patients ayant une TCPT.

## Patients et méthodes

Notre étude était rétrospective, menée sur deux ans, dans le Service de réanimation polyvalente du CHU Habib Bourguiba de Sfax. Nous avons inclus tout patient ayant une TCPT confirmée par une angioTDM ou angioIRM cérébrale.

## Résultats

L’incidence de la TCPT a été de 5,09 %. La tranche d’âge la plus touchée a été celle inférieure à 20 ans (30,4 %). Le délai moyen de diagnostic de la TCPT a été de 2,22 ± 3,9 jours. Le diagnostic a été fait dès l’admission sur *bodyscanner* chez 15 patients (65,2 %). La localisation la plus fréquente a été le sinus latéral chez 16 patients (69,9 %). Les fractures crâniennes ont été associées aux TCPT chez 21 patients (91,3 %). Le délai moyen de prescription de l’héparinothérapie a été de 3,1 ± 1,8 jours. L’hyperplasie nodulaire focale (HNF) a été prescrite chez 18 patients (78,3 %) avec une dose moyenne de 123,2 ± 82,6 mg/j. La durée de séjour en réanimation a été en moyenne de 17,4 ± 19,8 jours. Le taux de létalité a été de 17,4 %.

## Conclusion

La TCPT est une complication rare et grave. Elle doit être cherchée chez les patients ayant une fracture crânienne surtout en rapport avec les sinus veineux. Le traitement reste un challenge entre une anticoagulation curative nécessaire et la gestion de l’hémostase chez un traumatisé crânien.

# Research article: N° 93 La césarienne chez les femmes migrantes et l'accès aux soins périnataux : revue systématique couvrant la région MENA *(Middle East and North Africa)*

**Published:** 2024-06-13

**Authors:** Anissa OUAHCHI, Stella EVANGELIDOU, Farah SEEDAT, Liv PAMPIRI, Abdedayem KHELIFI, Mohamed DOUAGI, Wejdène MANSOUR, Ali MTIRAOUI, Dominik ZENNER, Bouchra ASSARAG

**Affiliations:** 1 Office national de la famille et de la population, Tunis, Tunisie; 2 Barcelona Institute for Global Health, Rosselló, 132, 7è, 08036 Barcelona, Espagne; 3 St George’s University of London, St George’s Hospital, Cranmer Terrace, London SW17 0RE, Royaume-Uni; 4 The University of Copenhagen, N0rregade 10, DK-1165 Copenhagen, Danemark; 5 Faculté de médecine de Sousse, RJHG+9G4, Av. Mohamed Karoui, Sousse 4002, Tunisie; 6 Queen Mary University London, 327 Mile End Rd, Bethnal Green, London E1 4NS, Royaume-Uni; 7 École nationale de santé publique, Rue Lamfadel Cherkaoui, Madinat Al Irfane, Rabat BP-6329, Maroc

## Introduction

Des disparités dans l’accès aux services de santé maternelle, notamment en matière de césarienne, ont été observées parmi les femmes migrantes dans la région MENA. Nous avons mené une revue systématique pour explorer la proportion de césariennes.

## Patients et méthodes

Nous avons effectué une recherche documentaire primaire dans six bases de données bibliographiques électroniques, dans la littérature grise, y compris les principaux sites Web internationaux et nationaux de la région, de 2000 à 2023. Cette recherche a porté sur les conditions de santé maternelle/néonatale des femmes enceintes/post-partum migrantes. Nous avons procédé à une évaluation de la qualité à l’aide des outils JBI, AACODS et AGREE.

## Résultats

Nous avons inclus 11 études parmi les 2 731 identifiées. Elles portaient sur les femmes migrantes/réfugiées/déplacées. Une des trois études qui ont comparé les migrantes et la population hôte a révélé que le nombre de césariennes était significativement plus élevé chez les Syriennes. Trois études ont montré que les césariennes ont augmenté. Dans l’une d’elles, le taux de césariennes chez les réfugiées palestiniennes en Jordanie, en Cisjordanie, à Gaza, en Syrie et au Liban étaient supérieurs aux niveaux recommandés.

Trois études ont montré que les césariennes étaient dues à des complications résultant de l’accès réduit des Palestiniennes et des Syriennes aux soins prénatals. Deux études qualitatives ont montré que les réfugiées syriennes, irakiennes et palestiniennes préfèrent l’accouchement par voie basse.

## Conclusion

Les femmes migrantes dans la région MENA présentent des taux élevés et parfois inappropriés de césariennes. Une attention est nécessaire pour éliminer les obstacles aux soins rapportés et garantir un accès équitable aux migrantes de la région.

# Research article: N° 94 Expériences en santé sexuelle et reproductive chez des femmes des quartiers « politique ville » des quartiers nord de Marseille

**Published:** 2024-06-13

**Authors:** Oluwafêmi Julie Marie-Josée HESSOU, Patrick THONNEAU

**Affiliations:** Université Senghor, Équipe de recherche EA 3614, Centre hospitalier universitaire (CHU) de Toulouse, France

## Introduction

Il est reconnu que la santé sexuelle et reproductive est largement influencée par les déterminants sociaux. En France, les femmes vivant dans les quartiers dits « prioritaires de la politique de la ville » font face à des problèmes spécifiques à leurs territoires de vie (précarité, environnement, provenance géographique), qui influencent leur santé sexuelle et reproductive.

## Méthodologie

Dans une démarche socioanthropologique qualitative, des entretiens ont été menés auprès de 14 femmes, d’origine maghrébine et africaine, résidant dans le quartier « politique de la ville » de Saint Barthélémy-Bon Secours à Marseille (quartier nord), sur la sexualité, le recours aux soins gynécologiques, les violences de genre.

## Résultats

Pour ces femmes, la sexualité reste un sujet tabou, et leurs expériences reproductives, en particulier le recours à l’IVG, sont normées par les convictions religieuses. L’utilisation de la contraception est influencée par les informations et perceptions propres aux femmes.

Le recours aux services en santé sexuelle et reproductive est conditionné par la situation administrative pour les personnes étrangères, le manque d’informations sur les droits sociaux, et la dépendance socioéconomique vis-à-vis du conjoint.

Ces conditions sont des facteurs de risque impactant négativement la santé sexuelle et reproductive des femmes à l’instar des violences conjugales, de la restriction aux services de soins, et des violences verbales à l’endroit de ces femmes d’origine étrangère.

## Conclusion

Le partage de leurs vécus et de leurs histoires reproductives dans un cadre associatif adapté et encadré pourrait constituer une alternative d’information et de sensibilisation pour ces femmes en situation de précarité.

# Research article: N° 95 Transplantation d'organes en Tunisie : évolution de l'activité de prélèvement à partir de donneurs en état de mort encéphalique

**Published:** 2024-06-13

**Authors:** Wafa AISSI, Sami TALBI, Mohamed Hedi FAKRAOUI, Halima YAHMADI, Boutheina ZANNAD, Mejda CHAOUACHI, Hechmi TEMIMI, Jalel ZIADI

**Affiliations:** Service d’information et de recherches, Centre national pour la promotion de la transplantation d’organes, Tunis, Tunisie

## Introduction

En Tunisie, la transplantation d’organes a connu de grands progrès ces dernières décennies. Cependant, les besoins sont loin d’être satisfaits en raison de la pénurie d’organes. L’objectif de ce travail était de décrire l’évolution de l’activité de prélèvement d’organes à partir d’un donneur décédé sur une période de 13 années.

## Patients et méthodes

Il s’agit d’une étude rétrospective incluant toutes les déclarations de suspicion de mort encéphalique entre le 1^er^ janvier 2010 et le 31 décembre 2022. La collecte de données a été faite au Centre national pour la promotion de la transplantation d’organes par les médecins coordinateurs nationaux moyennant une fiche standardisée. L’analyse des données a été faite par le logiciel SpSs.

## Résultats

Le nombre total de déclarations de donneurs potentiels d’organes était de 1 046 avec des variations entre les années et une moyenne de 80 par an. Au total, 457 morts encéphaliques ont été confirmées avec un chiffre annuel variant entre 18 (en 2021) et 74 (en 2022) et une moyenne de 35 par an. Le nombre total de prélèvements multi-organes réalisés était de 90 et le nombre annuel variait entre 1 (en 2012 et 2020) et 19 (en 2022). Le taux de refus de prélèvement d’organes après l’approche de la famille était maximal en 2012 et 2020 (année de la pandémie de Covid-19).

## Conclusion

L’activité de prélèvement à partir d’un donneur décédé a connu de grandes variations. Elle a été fortement affectée par la pandémie Covid-19 pour reprendre ensuite progressivement et atteindre un pic en 2022.

# Research article: N° 96 Les raisons motivant le refus du don d'organe par les familles des patients en état de mort encéphalique en Tunisie : résultats d'une étude nationale

**Published:** 2024-06-13

**Authors:** Wafa AISSI, Sami TALBI, Mohamed Hedi FAKRAOUI, Halima YAHMADI, Boutheina ZANNAD, Mejda CHAOUACHI, Hechmi TEMIMI, Jalel ZIADI

**Affiliations:** Service d’information et de recherches, Centre national pour la promotion de la transplantation d’organes, Tunis, Tunisie

## Introduction

En Tunisie, le don d’organes à partir d’un donneur décédé rencontre encore de sérieuses réticences auprès de la population. En vue de rechercher la non-opposition au don d’organes d’un patient en état de mort encéphalique, une approche de famille est réalisée. L’objectif de ce travail était d’identifier les causes de refus du don d’organes par les familles.

## Patients et méthodes

Il s’agit d’une étude rétrospective incluant toutes les approches de familles réalisées après confirmation de la mort encéphalique à la recherche de nonopposition au prélèvement d’organes entre le premier janvier 2019 et le 31 décembre 2022. La collecte de données a été faite au Centre national pour la promotion de la transplantation d’organes par les médecins coordinateurs nationaux, moyennant une fiche standardisée à partir des dossiers de déclaration de mort encéphalique.

## Résultats

Cent-quarante approches de famille ont été réalisées après confirmation de la mort encéphalique par angioscanner cérébral dans tous les cas. Le refus du don a été recueilli dans 94 cas (67,1 %). Les taux de refus des années 2019, 2020, 2021 et 2022 étaient respectivement de 59,1 %, 94,4 %, 76,5 % et 62,3 %. Différentes causes de refus ont été observées; la cause la plus fréquente était la crainte du non-respect de l’intégrité du corps (66 %) suivie par le manque de confiance dans le système de santé (6,4 %).

## Conclusion

Le taux de refus de don d’organes à partir d’un donneur décédé était assez élevé. Les raisons de refus étaient surtout liées à la représentation du corps du défunt.

# Research article: N° 97 Le trouble de stress posttraumatique chez les personnes migrantes

**Published:** 2024-06-13

**Authors:** Vladimir ADRIEN, Claire TANTET, Ornella MILLELIRI, Thierry BAUBET, Olivier BOUCHAUD, Nicolas VIGNIER

**Affiliations:** Service de maladies infectieuses et tropicales, Hôpital Avicenne, AP-HP, Bobigny, France

Les personnes migrantes sont souvent confrontées à des événements de vie stressants ou traumatiques dans leur pays d’origine, du fait du déracinement lié à l’exil, des violences subies durant l’expérience migratoire, et enfin à leur arrivée en France, du fait d’un statut de séjour souvent précaire. Les syndromes psychotraumatiques touchent entre 5 et 10 % des personnes migrantes et se compliquent fréquemment d’épisodes dépressifs ou anxieux, de conduites à risques et d’addictions, ainsi que de répercussions physiques invalidantes. Les troubles de la cognition sociale accompagnant également ces troubles entravent les capacités d’orientation et d’intégration dans le pays d’accueil. Les syndromes psychotraumatiques dont souffrent les migrants sont pour la plupart complexes, conséquence d’une exposition répétée au danger vital. La symptomatologie est bruyante, les migrants se trouvant en état de « survie » jusqu’à l’obtention d’un statut administratif et de conditions de vie stables. À ce moment, la mémoire traumatique peut venir réveiller de nouveaux symptômes. La prise en soins des patients est d’autant plus difficile que les ressources sont limitées (isolement social, barrière de la langue, choc culturel, incertitude matérielle et statutaire).

La reconsolidation défectueuse de la mémoire traumatique peut par ailleurs entraver la capacité des patients à produire un récit incarné, compromettant l’obtention du droit d’asile.

Nous présenterons dans un premier temps la physiopathologie de la genèse du trouble de stress post-traumatique en mettant l’accent sur les différentes phases de la vie des personnes migrantes, pour en détailler secondairement les enjeux thérapeutiques d’un point de vue général et spécifique de cette population.

# Research article: N° 98 Mise en place de contrôles de qualité en microbiologie : retour de mission humanitaire Biologie sans Frontières (BSF) à Bangwa (Cameroun)

**Published:** 2024-06-13

**Authors:** Elisabeth ROTH-JARROUX

**Affiliations:** Laboratoire du Centre hospitalier de la Dracénie, Route de Montferrat, 83300 Draguignan, France

Cette mission s’est déroulée sur le site de l’hôpital protestant de Bangwa (Cameroun). Elle faisait suite à un audit réalisé précédemment par une autre équipe ayant permis d’évaluer les besoins et surtout de pouvoir travailler en adéquation avec leurs possibilités. Nous étions deux intervenantes : une en microbiologie et une en hématologie/biochimie et hémostase. Notre mission a consisté à la sensibilisation du personnel à la démarche qualité par l’intermédiaire de formations pratiquées sur site, par la présentation par *power point* d’une sensibilisation à l’utilisation de contrôles de qualité. Pour la partie microbiologie, nous avions en emmené des souches ATCC d’*Enterococcus faecalis, Escherichia coli, Staphylococcus aureus* et *Klebsiella pneumoniae.* Ces souches ont été repiquées et des antibiogrammes ont été réalisés et comparés aux résultats du Comité de l’antibiogramme de la société française de microbiologie (CA-SFM). Nous avons pu former une dizaine de techniciens, tous très impliqués et consciencieux. Nous nous sommes adaptées à leurs moyens. Nous les avons aidés également à améliorer le rendu des résultats en axant sur les pathogènes à étudier. Concernant les identifications bactériennes, nous avons également revu les pratiques. Cette mission a eu lieu en mai 2022. BSF devant réaliser au bout de deux ans un suivi de ces missions, nous retournerons sur site en 2024 pour voir l’évolution des pratiques, échanger à nouveau avec les techniciens et le personnel hospitalier. Notre devise : « Développer pour ne plus assister ».
